# Coverage-Aware Break-Even Conditions for Polymeric Membrane-Based Water Reuse in Arid Urban Systems

**DOI:** 10.3390/polym18212773

**Published:** 2026-11-06

**Authors:** Toqeer Ali Syed, Ali Alatify, Ali Akarma, Muhammad Tayyab Naqash, Abdulaziz Alqurashi

**Affiliations:** 1 AI Center, Faculty of Computer and Information Systems, Islamic University of Madinah, Madinah 42351, Saudi Arabia; toqeer@iu.edu.sa (T.A.S.); 443059463@stu.iu.edu.sa (A.A.); 2 Department of Civil Engineering, Prince Sattam Bin Abdulaziz University, Al-Kharj 11942, Saudi Arabia; 3 AI V&V Lab, King Fahd University of Petroleum and Minerals, Dhahran 31261, Saudi Arabia; 4 Civil Engineering Department, Faculty of Engineering, Islamic University of Madinah, Madinah 42351, Saudi Arabia; mnaqash@iu.edu.sa (M.T.N.); amalqurashi@iu.edu.sa (A.A.)

**Keywords:** polymeric membranes, ultrafiltration, reverse osmosis, membrane-based water reuse, water reclamation, membrane fouling, cost-effectiveness, break-even analysis, global sensitivity analysis, urban water systems

## Abstract

A utility reclaiming effluent with polymeric membranes must choose a configuration and a deployment coverage. A coverage-aware volume and cost model for an illustrative arid zone of $10\;{\mathrm{Mm}}^{3}$/yr compares ultrafiltration (Configuration A) with ultrafiltration plus polyamide thin-film-composite reverse osmosis (Configuration B). Membrane parameters are literature-derived, eligibility and placement assumed, and no experiments were performed. At 50% coverage and 1 USD/m^3^, Configuration A avoids 11.5 percentage points of potable input at 0.48 USD/m^3^ and Configuration B 20.4 points at 0.70 USD/m^3^, or 0.56 and 0.82 USD/m^3^ without real-loss credit. Break-even coverages are 11.7% and 11.5%. Configuration B is preferable above a crossover value of 0.98 USD/m^3^ (1.15 without loss credit) and leads in 46.2% of paired draws at 1 USD/m^3^. Feed salinity, regulation, concentrate cost, and capital and financing costs move the crossover most. Under independent inputs and assumed eligibility, approved demand rather than recovery limits volume in at least 98.8% of draws, and placement dominates net-benefit variance. Adverse feed conditions that couple operating and cost parameters make treatment costs comparably influential for Configuration B. The thresholds are conditional criteria, not city-specific predictions.

## 1. Introduction

Arid cities that depend on desalinated and conveyed supply have a large and predictable secondary-effluent stream that is already inside the urban boundary. Polymeric membranes are the established way to convert that stream into water of a defined and certifiable quality. Ultrafiltration membranes cast from polyvinylidene fluoride (PVDF) or polyethersulfone (PES) remove suspended solids, turbidity, and particle-associated pathogens, and polyamide thin-film-composite reverse-osmosis membranes additionally reject dissolved salts and a large fraction of dissolved organic micropollutants [1,2,3,4]. The materials science of these membranes is mature enough that full-scale municipal reclamation plants are routine.

The decision a utility faces is not whether the membranes work. It is whether a reclamation scheme that reaches only part of a service zone saves enough potable water to repay its cost, and which membrane class to install. These two questions are coupled through water quality. Ultrafiltration alone does not change salinity, so its product is acceptable for restricted landscape irrigation and toilet flushing but not for salinity-sensitive amenity planting or for cooling towers that must run at reasonable cycles of concentration. Adding reverse osmosis widens the set of approved end uses and therefore the volume of potable water that can be displaced, at the price of higher capital cost, higher specific energy, an additional membrane-replacement stream and a concentrate that must be managed inland [5,6]. The material choice is thus an economic choice about how much reuse-eligible demand the product water can legally and technically serve.

Membrane studies usually report performance endpoints: permeability, rejection, flux decline, fouling resistance, energy per cubic meter. Techno-economic studies report levelized cost at an assumed plant scale and utilization. Neither endpoint answers the deployment question, because the reclaimed distribution network is built incrementally, the eligible demand is unevenly distributed across urban sectors, and the fixed costs of monitoring and compliance do not scale down. A scheme that is attractive at full build-out can be uneconomic at the coverage a utility can actually finance in its first phase.

This paper addresses that gap. We develop a coverage-aware volume and cost model in which the recoverable volume is set jointly by membrane water recovery, the available effluent stream, and the reuse-eligible demand that the reclaimed network reaches, and in which the cost carries the membrane-specific terms that the Special Issue’s practical concerns identify: element capital and replacement interval, cleaning, a fouling penalty on energy and chemicals, and concentrate disposal. We derive break-even conditions in closed form or as a one-dimensional root, evaluate them across declared scenario distributions with coverage treated as the utility’s decision, and compute variance-based sensitivity indices conditional on coverage. We then test the structural assumptions on which these results rest, including dependence among membrane operating and cost parameters, feed salinity, membrane aging, network structure and zone size, and express the configuration choice in decision-theoretic terms.

The contribution is threefold. First, we state the conditions under which a partially deployed polymeric membrane reuse scheme breaks even, including a break-even unit treatment cost and a break-even membrane element life that can be compared directly with supplier data. Second, we show that the choice between ultrafiltration alone and ultrafiltration with reverse osmosis is decided by a crossover in the marginal value of water, locate that crossover, and report the expected regret and value of information around it. Third, we identify which parameters govern the decision and under which conditions. Under the declared ranges and independent inputs, whether a scheme pays is governed mainly by the placement of its product water rather than by membrane performance. The choice between the configurations also depends on the cost premium of desalting and on the eligible demand that desalting adds, which are determined by concentrate management, capital and financing costs, element life, energy price, feed salinity and the regulatory regime. We close with a measurement protocol for a candidate pilot. Madinah, whose sector mix, arid climate and dependence on desalinated supply make it a relevant setting, serves as an illustrative decision context rather than a case study.

We did not perform new membrane experiments. Every membrane property used here is either literature-derived or an explicitly declared planning assumption, and the analysis is a decision screen rather than a validated prediction for any particular plant.

Section 2 reviews the relevant membrane and reuse literature and states the gap. Section 3 defines the two configurations, their water-quality roles, and the model. Section 4 reports the results. Section 5 describes the monitoring and governance layer a reclaimed scheme requires, and Section 6 turns the conditions into a measurement protocol. Section 7 discusses implications, the link between polymer membrane properties and the decision variables, and limitations, and Section 8 concludes. Appendix D, Appendix E and Appendix F document the settings of the scenario analyses, the monthly balance, and the pilot power calculations.

## 2. Polymeric Membranes for Water Reuse: Background and Research Gap

### 2.1. Membrane Materials and Their Separation Roles

Pressure-driven polymeric membranes span a continuum of selectivity. Ultrafiltration membranes, typically hollow fibers spun from polyvinylidene fluoride or polyethersulfone with nominal pore sizes near 0.01–0.1 μm, separate by size exclusion and retain suspended solids, colloids, protozoan cysts, and most bacteria, while passing dissolved salts and small organic molecules essentially unchanged [4,7]. Reverse-osmosis membranes are thin-film composites in which an aromatic polyamide layer a few hundred nanometers thick is interfacially polymerized onto a porous polysulfone support. Transport is by solution-diffusion, and rejection of sodium chloride above 99% is standard for current elements [1,2]. The distinction matters here because it determines which urban end uses the product water may serve, and therefore how much potable water a reuse scheme can displace.

### 2.2. Water-Quality Objectives and the Limits of Rejection

Rejection is not purification. For neutral low-molecular-weight organics, rejection by polyamide reverse osmosis depends on molecular size, charge, and hydrophobicity, and falls markedly for small uncharged solutes [8]. N-nitrosodimethylamine is the standard counterexample: it passes reverse-osmosis membranes to a substantial degree and requires downstream ultraviolet treatment in potable-reuse trains [9]. Perfluoroalkyl acids are rejected well by tight nanofiltration and reverse-osmosis membranes, but rejection varies with chain length, flux, and fouling state [10]. Ultrafiltration retains microplastic particles by size exclusion, although polymeric ultrafiltration systems can themselves shed polymer fragments under aggressive backwash and cleaning regimes [11]. Boron and other weakly dissociated species are poorly rejected at neutral pH. The practical consequence for a reuse scheme is that the membrane class fixes a quality class, the quality class fixes the set of approved end uses, and residual constituents may require additional barriers before particular uses are permitted.

This constraint is sharper in the Saudi context, where conventional treatment alone does not reliably remove bacterial contaminants and antibiotic-resistance genes from effluent intended for irrigation [12], and where most of the irrigation water sampled in an oasis that blends groundwater, treated wastewater, and agricultural drainage carried a high or very high salinity hazard [13]. Both findings support a membrane barrier, and the second is the specific reason a desalting stage may be required rather than optional. Irrigation-water guidelines place no restriction on use below about 450 mg/L of total dissolved solids, a slight-to-moderate restriction between 450 and 2000 mg/L, and a severe restriction above 2000 mg/L [14]. Because ultrafiltration does not change salinity, the salinity of the feed largely decides which irrigation uses an ultrafiltered product can serve, and therefore how much eligible demand the desalting stage adds.

### 2.3. Fouling, Element Life and Operating Cost

Fouling is the dominant operational problem in membrane reuse. Organic, colloidal, inorganic, and biological fouling raise transmembrane pressure, reduce permeate flux, increase cleaning frequency, and shorten element life [15,16]. Inorganic fouling, or scaling, of pressure-driven membrane processes has been reviewed critically [17]. It depends on feed composition and recovery, and on the residence time of the concentrate relative to the nucleation induction time of sparingly soluble salts, so operating strategy can itself mitigate it, as batch operation of reverse osmosis does [18]. Fouling is also not static: an irreversible component accumulates between cleanings over an element’s life, so the energy and cleaning burden rises with element age.

Polymer degradation adds a second aging pathway. Polyamide has a low tolerance to oxidants, especially chlorine, whose attack on the amide and aromatic groups reduces salt rejection [16]. Hypochlorite aging at an exposure of 500 g·h/L increased the permeability of a polyethersulfone ultrafiltration membrane 3.8- to 5.8-fold and reduced its humic-acid retention from 59% to 20–37%, whereas hydrogen peroxide aging had little effect [19]. Accelerated aging of a PVDF ultrafiltration membrane fouled with tannic acid and cleaned with sodium hydroxide every 15 days gave a modeled lifetime of 1.8 years for a 10% loss of tensile strength and about 7 years for a 30% loss [20]. Reverse-osmosis element lifespans are often limited to 5 to 10 years [16], and end-of-life elements are themselves a waste stream with recognized recycling and energy-recovery routes [21].

Pretreatment selection is the principal control, and ultrafiltration ahead of reverse osmosis is used precisely to stabilize the downstream feed [22]. Fouling and degradation therefore enter an economic model through three channels: a higher specific energy at a given flux, more frequent cleaning, and more frequent element replacement, together with a fourth for reverse osmosis: a loss of salt rejection that can take the product out of its approved quality class. We represent the first two channels with a fouling penalty multiplier and the third through an explicit element service life. Progressive irreversible fouling, coupled operating parameters, and salt-passage aging linked to the quality class are tested as scenarios in Section 4.10, and Section 7.6 relates these material properties to the decision variables.

### 2.4. Cost and Energy of Municipal Reuse

Process-level techno-economic analysis of municipal reuse reports a levelized cost of about 0.54 USD/m^3^ and an electricity intensity of about 0.84 kWh/m^3^ for a reverse-osmosis-based train, against 0.40 USD/m^3^ and 0.30 kWh/m^3^ for a carbon-based train without desalting [5]. Tertiary ultrafiltration alone consumes on the order of 0.16 kWh/m^3^ on municipal effluent [7]. Unit costs fall with scale over the range relevant to district schemes [23], and reclaimed water delivered for irrigation in Mediterranean settings has been costed at roughly 0.25–0.30 EUR/m^3^ including treatment and conveyance [24]. Inland reverse-osmosis concentrate has no low-cost sink and carries its own management burden [6], and adding zero liquid discharge to a facility that discharges its concentrate to surface water has been estimated to approximately double the levelized cost of water [5]. National assessments of reuse potential emphasize that the binding constraints are frequently distribution, end-use approval, and public acceptance rather than treatment capability [25,26,27].

### 2.5. Deployment Economics of Partially Built Reuse Schemes

Water economics compares conservation instruments on a common cost basis [28], and price-response meta-analysis places residential elasticities well below unity in magnitude [29]. Pressure management is the standard physical-loss comparator and is among the cheaper loss-control measures [30,31], with non-revenue water defined through the International Water Association balance [32] and estimated at about 30% of system input worldwide [33]. When the parameters that decide an investment cannot be measured before deployment, water-planning practice recommends stating the conditions under which each option succeeds, using scenario discovery to locate failure regions [34] and variance-based sensitivity analysis to apportion output variance while separating decision variables from uncertain inputs [35].

### 2.6. Research Gap and Positioning

Three gaps follow.

1. Coverage-aware economics of a membrane scheme. The cited techno-economic studies evaluate treatment trains at an assumed scale and utilization [5,23]. They do not state the deployment coverage at which a partially built reclaimed network begins to repay a membrane plant whose monitoring and compliance costs do not scale down.
2. Membrane class as an economic decision. The cited materials literature characterizes what each membrane class rejects [1,2,8]. It does not express that difference as a decision rule over the marginal value of water and the composition of urban demand.
3. Transparent separation of evidence types. Recovery, specific energy, element life, and cost are literature-derived, whereas reuse eligibility, deployment concentration, and the placeable share of product water are planning assumptions. We keep these categories separate throughout and report which of them the decision actually depends on.

The contribution is the combination of these elements in a decision screen. We do not claim novelty for the membrane processes, the accounting identities, or the sensitivity estimators, each of which is standard.

## 3. Materials and Methods

### 3.1. Why the Analysis Reports Conditions Rather than Site-Specific Predictions

No operational telemetry, effluent characterization, or procurement quotation from the zone of interest was available to this study, and no membrane experiments were performed for it. The analysis therefore uses a transparent volume and cost model with declared scenario distributions rather than fitted site-specific distributions. It derives thresholds for cost recovery and shows how those thresholds move across the declared inputs. The thresholds are candidate decision criteria for later field measurement. They are not estimates of realized savings, plant performance or failure probabilities for Madinah or any other city.

Madinah serves only as an illustrative decision context. The reference service zone, its sector mix and the four further district scenarios are constructed: no sector-level billing, end-use or effluent data from Madinah were available to derive them, and their representativeness for any actual district is unknown. For this reason, the configuration decision is reported across five constructed district mixes, three regulatory eligibility scenarios and a range of zone sizes rather than for a single district.

### 3.2. The Two Polymeric Membrane Configurations

**Figure 1:**
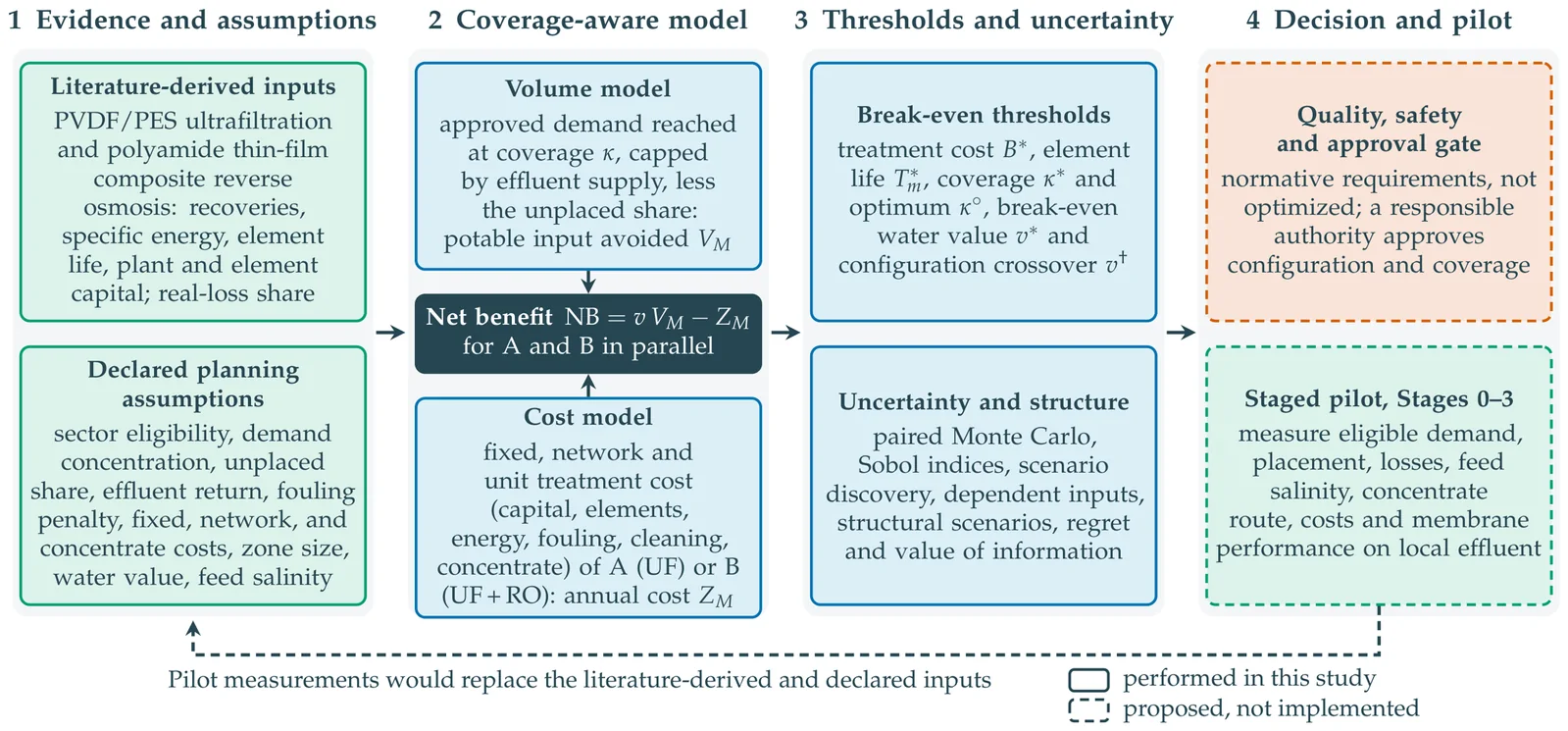
Workflow of the coverage-aware break-even analysis. Literature-derived inputs and declared planning assumptions (1) enter a volume model and a cost model, which are evaluated in parallel for both configurations and combined into the annual net benefit (2). The net benefit yields the break-even thresholds and the uncertainty, structural, and decision analyses (3), which inform a normative quality and safety gate and a staged pilot (4). Dashed elements are proposed and were not implemented in this study. Pilot measurements would replace the literature-derived and declared inputs. Symbols are defined in Section 3.4, Section 3.5 and Section 3.7. PVDF, polyvinylidene fluoride; PES, polyethersulfone; UF, ultrafiltration; RO, reverse osmosis.

Table 1 defines the two configurations evaluated. Both take screened, biologically treated secondary municipal effluent as feed. Configuration A is coagulation followed by polymeric ultrafiltration and disinfection. Configuration B adds a polyamide thin-film-composite reverse-osmosis stage, for which the ultrafiltration stage now also serves as pretreatment, followed by stabilization and disinfection. Figure 1 shows how the treatment chain connects to the deployment decision.

The configurations are not presented as a ranking of materials. They are the two quality classes that a utility in an arid inland city realistically chooses between, and the entire economic difference between them follows from what each membrane rejects.

Two qualifications apply throughout. First, rejection does not imply complete purification: the residual constituents listed in Table 1 may require an additional barrier, such as ultraviolet treatment or an advanced oxidation step, before particular end uses are approved [9]. Second, treatment suitability depends on the feedwater as much as on the membrane. The eligibility weights used below therefore represent an approval decision by a regulator, not a claim about what either membrane can remove.

### 3.3. Reuse-Eligible Demand by Sector

Let $d_{i}$ denote annual baseline potable demand in urban sector *i*, $D=\sum_{i}d_{i}$ and $w_{i}=d_{i}/D$. Table 2 gives, for each sector, the share $e_{i}\in \left[0,1\right]$ of its potable demand that certified reclaimed water of the relevant quality class may replace. Two sets of weights are used, one per configuration, because the approved end uses differ. Scaled by a common factor ε, the demand-weighted reuse-eligible share is given by Equation (1):

(1) $${\bar{e}}_{\varepsilon }=\sum_{i}w_{i}\;{\tilde{e}}_{i},\;{\tilde{e}}_{i}=\min\left\{1,\varepsilon e_{i}\right\}.$$

At ε=1 and the reference sector mix, $\bar{e}=0.1745$ for Configuration A and 0.3085 for Configuration B. Ablution water in mosques is excluded from both sets because it serves a ritual function with a potable requirement. Only cleaning, flushing, and grounds irrigation in those facilities are treated as eligible. Healthcare is eligible only under Configuration B and only for cooling-tower makeup.

These weights are author-declared scenario assumptions that encode a regulatory judgment. No regulatory determination, sector end-use survey, or expert-elicitation record available to this study reports them, and none of them is measured. To make them traceable, each weight is built as the sum of the end uses that the quality class is assumed to serve: irrigation, cooling-tower makeup, and flat-rate non-potable uses such as toilet flushing, cleaning, and washdown (Table A2, Appendix D). For example, the residential weight of 0.10 assumes that communal landscape irrigation, 6% of residential potable demand, and toilet flushing in dual-plumbed buildings, 4%, are approved. Their uncertainty is represented in three complementary ways: the common multiplier ε, which represents a systematic shift in the regulatory position across all sectors; independent sector-specific multipliers $\varepsilon_{i}$, which represent sector-by-sector errors; and three discrete regulatory scenarios, restrictive, reference, and permissive, whose weights are given in Table A3 (Section 4.10).

### 3.4. Recoverable Volume

With real losses at a share $\lambda_{p}$ of potable system input, as defined by the International Water Association balance [32], the status-quo system input is $Q_{0}=D/\left(1-\lambda_{p}\right)$ and the real-loss volume is $L_{0}=\lambda_{p}Q_{0}$. Collected secondary effluent available as membrane feed is ϕD, where ϕ is the effluent return ratio. With overall train water recovery *R*, the annual product water that the plant could deliver at full build-out is RϕD. For Configuration A, $R=R_{\mathrm{UF}}$, the recovery of the ultrafiltration stage including backwash and cleaning losses. For Configuration B, $R=R_{\mathrm{UF}}R_{\mathrm{RO}}$, where $R_{\mathrm{RO}}$ is the recovery of the reverse-osmosis stage. The volume that can actually be placed at full build-out is bounded by both supply and approved demand, as stated in Equation (2):

(2) $$V^{\max}=\min\left\{R\;\phi \;D,\;{\bar{e}}_{\varepsilon }\;D\right\}.$$

The effluent supply term binds if and only if $R\phi <{\bar{e}}_{\varepsilon }$, that is, when the product that the train can make from the collected effluent is smaller than the approved demand. Equivalently, supply binds below the recovery $R^{{}^{\circ}}={\bar{e}}_{\varepsilon }/\phi$ or below the effluent return ratio $\phi^{{}^{\circ}}={\bar{e}}_{\varepsilon }/R$, quantities that a utility can evaluate before procurement. At the nominal recoveries and ϕ=0.60, supply binds only if ${\bar{e}}_{\varepsilon }$ exceeds 0.552 for Configuration A or 0.450 for Configuration B. At the reference mix, this would require ε≥4.47 or ε≥1.89, far outside the declared range, or an effluent return ratio below 0.190 or 0.411.

The reclaimed distribution network is built incrementally. Let κ∈[0,1] denote deployment coverage, defined throughout as the share of reuse-eligible connection equivalents that are physically connected to the reclaimed network and approved to receive its water. This is a single, explicit definition: it is not a share of all connections, not a share of total demand, and not a plant capacity factor. The number of eligible connection equivalents is $M_{e}=\sum_{i}e_{i}d_{i}/{\bar{v}}_{i}$ with ${\bar{v}}_{i}$ the declared annual potable volume per connection in sector *i*. Counting connections in proportion to eligible volume is exact where eligible demand is served through dedicated connections, such as communal irrigation points and landscaping meters. It is a lower bound where a large user, such as a hotel, hospital, or office building with a cooling tower, takes only part of its demand as reclaimed water but still needs a full service connection. Section 4.10 reports the alternative that counts every non-residential user with an eligible use as a whole connection. Plant capacity is sized to the product volume delivered at each coverage (Equation (4)), so treatment capacity and network coverage advance together and are not separate decision variables in the baseline. Section 4.10 relaxes this with discrete treatment trains.

**Figure 2:**
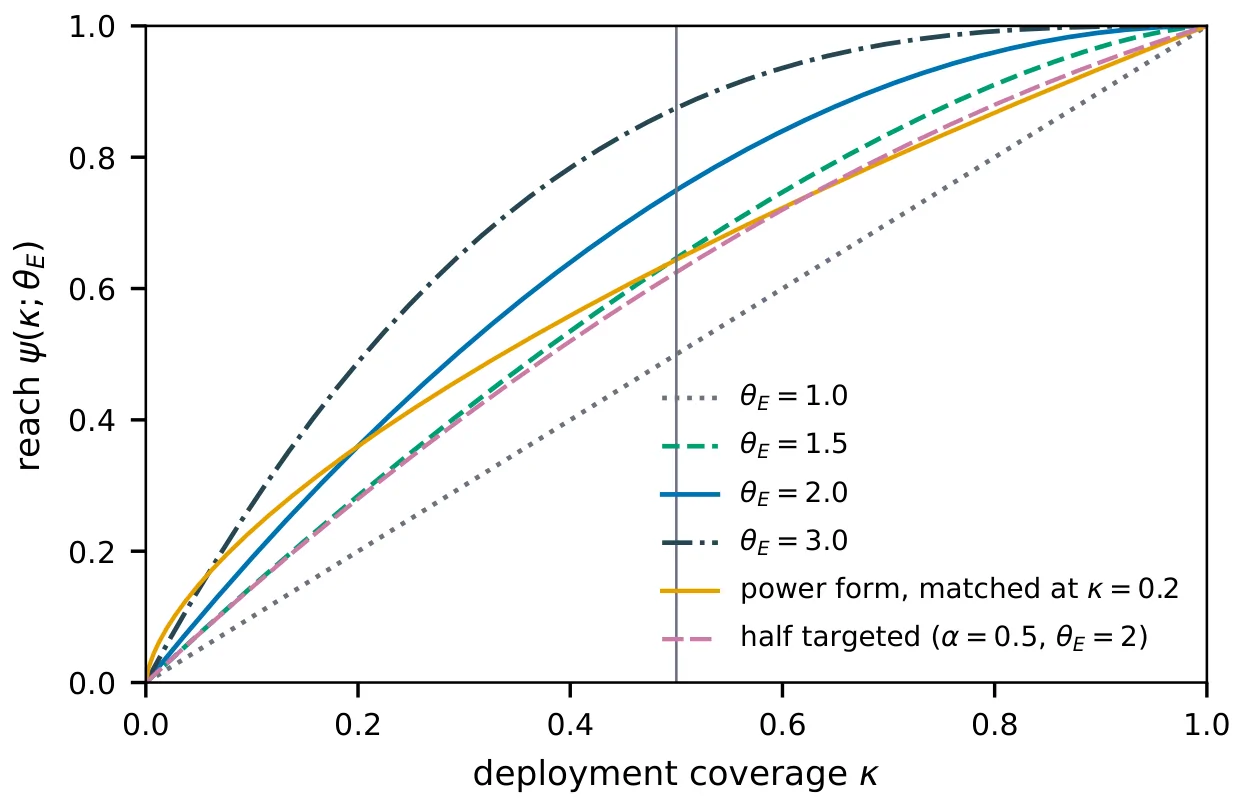
Reach function $\psi (\kappa ;\theta_{E})$ of Equation (3). The dotted line ($\theta_{E}=1$) is the untargeted connection order. The vertical line marks κ=0.50, at which the nominal concentration $\theta_{E}=2$ gives a reach of 0.750. The orange curve is the alternative power form $\kappa^{\tau }$ used in the robustness analysis, matched to $\theta_{E}=2$ at κ=0.2, and the purple dashed curve is partially targeted order with α=0.5 and $\theta_{E}=2$.

When a utility connects large eligible users first, the share of eligible demand reached at coverage κ is represented by the concave reach function of Equation (3),

(3) $$\psi \left(\kappa ;\theta_{E}\right)=1-{\left(1-\kappa \right)}^{\theta_{E}},\;\theta_{E}\geq 1,$$

where $\theta_{E}=1$ is untargeted connection order and larger $\theta_{E}$ represents greater concentration of eligible demand among the connections served first (Figure 2). No empirical concentration curve was available for the zone, so ψ is a stylized assumption rather than a calibrated function, and it does not represent pipe lengths, trunk mains, pumping, storage, or topography. Fully targeted order is also the most favorable case: network topology, equity requirements, and regulation may prevent a utility from connecting the largest eligible users first. Section 4.10 therefore reports partially targeted order, $\psi_{\alpha }\left(\kappa \right)=\alpha \;\psi \left(\kappa ;\theta_{E}\right)+\left(1-\alpha \right)\kappa$, in which only a share α of connections follows the targeted ranking, untargeted order (α=0) as a bound, an alternative power reach form, and alternative network-cost structures. Annual product water produced and distributed then follows from Equation (4):

(4) $$W\left(\kappa \right)=\psi \left(\kappa ;\theta_{E}\right)\;V^{\max}.$$

Equation (4) scales the full-build-out volume $V^{\max}$ by the reach, and it therefore embodies a deliberate assumption of proportional expansion. The plant, including its effluent intake, is built in step with the network, so that at coverage κ the installed capacity is the share $\psi (\kappa ;\theta_{E})$ of the capacity required at full build-out. The assumption is immaterial wherever approved demand binds at full build-out, ${\bar{e}}_{\varepsilon }\leq R\phi$. Then $V^{\max}={\bar{e}}_{\varepsilon }D$, and $W=\psi \;{\bar{e}}_{\varepsilon }D$ is exactly the approved demand reached. This holds for every nominal result at the reference mix, for every draw of Configuration A and for 98.8% of the draws of Configuration B (Section 4.4). Where the effluent supply binds at full build-out, a first phase that already has access to the whole collected effluent could deliver $W^{\mathrm{full}}\left(\kappa \right)=\min\left\{R\phi D,\;\psi \left(\kappa ;\theta_{E}\right)\;{\bar{e}}_{\varepsilon }D\right\}$, which is at least $\psi \left(\kappa ;\theta_{E}\right)\;V^{\max}$. In that case, Equation (4) understates the deliverable volume. We retain Equation (4) as the baseline because it describes a modular plant whose capacity and effluent intake are staged with the network (Section 7.4), and we report the full-access variant wherever the two forms differ (Section 4.4 and Section 4.8). Under full access, the net benefit keeps the form derived in Section 3.7, with $V^{\max}$ replaced by ${\bar{e}}_{\varepsilon }D$, up to the coverage at which the reach exhausts the effluent supply. Beyond that coverage, further connections add network cost without volume (Appendix A).

A share $\rho_{u}$ of that product cannot be placed because of seasonal mismatch between irrigation and cooling demand and a continuous effluent stream, losses in the reclaimed network, and end uses that are connected but not approved in every period. In the baseline, $\rho_{u}$ is a constant annual share common to both configurations. Appendix E derives configuration-specific values from a monthly balance with storage, and Section 4.10 reports their effect. Each cubic meter actually placed displaces one cubic meter of potable demand at the point of use. If real losses scale with system input, it also avoids the losses incurred in supplying that demand, $\lambda_{p}/\left(1-\lambda_{p}\right)$ cubic meters. If leakage is driven by pressure and is largely independent of throughput, it avoids none of them. With $\eta_{L}\in \left[0,1\right]$ the share of real losses credited as avoided, the annual avoided potable system input is given by Equation (5):

(5) $$V_{M}\left(\kappa \right)=\left(1-\rho_{u}\right)\;m_{L}\;W\left(\kappa \right),\;m_{L}=1+\frac{\eta_{L}\lambda_{p}}{1-\lambda_{p}},$$

which we also express as $\Delta_{M}=100\;V_{M}/Q_{0}$ in percentage points (pp) of system input. The loss multiplier $m_{L}$ carries the loss credit into every expression that follows. The full credit $\eta_{L}=1$, which gives $m_{L}=1/\left(1-\lambda_{p}\right)$, is appropriate for the marginal production and conveyance accounting of a supply-constrained system, but it overstates loss avoidance where leakage is largely independent of throughput. Because $\lambda_{p}$ is inferred indirectly from non-revenue-water information rather than from a local water balance (Appendix C), and because the credit is consequential, results are reported both with full credit, $\eta_{L}=1$, and with no credit, $\eta_{L}=0$ and $m_{L}=1$, throughout Section 4.

### 3.5. Cost Model

The annualized cost in Equation (6) has a fixed part, a part proportional to the product water treated, and a part proportional to the connections served:

(6) $$Z_{M}\left(\kappa \right)=F+B\;W\left(\kappa \right)+c_{\mathrm{net}}\;M_{e}\;\kappa .$$

Here *F* is the annual cost that does not scale with throughput, comprising the compliance and quality-assurance program, online monitoring, permitting, and core operating staff. The coefficient $c_{\mathrm{net}}$ is the annualized cost per connected eligible connection equivalent for the reclaimed distribution network, service connection and metering. The unit treatment cost *B*, in USD per cubic meter of product water, is given by Equation (7):

(7) $$B=\underset{\mathrm{plant}\;\mathrm{and}\;\mathrm{membrane}\;\mathrm{capital}}{\underset{︸}{\frac{\mathrm{CRF}\left(i,T_{p}\right)\;c_{\mathrm{cap}}+\mathrm{CRF}\left(i,T_{m}\right)\;c_{\mathrm{mem}}}{u}}}+\underset{\mathrm{operating}}{\underset{︸}{\pi_{E}\;\Phi \;\mathrm{SEC}+\Phi \;c_{\mathrm{cip}}+c_{\mathrm{om}}+c_{b}\;\left(\frac{1}{R_{\mathrm{RO}}}-1\right)}}.$$

The capital term annualizes plant capital $c_{\mathrm{cap}}$, expressed in USD per cubic meter per year of installed product capacity, over the plant life $T_{p}$, and membrane element capital $c_{\mathrm{mem}}$, on the same capacity basis, over the element service life $T_{m}$, where $\mathrm{CRF}\left(i,T\right)=i{\left(1+i\right)}^{T}/\left[{\left(1+i\right)}^{T}-1\right]$ is the capital recovery factor at real discount rate *i* and *u* is the annual utilization of installed capacity. Annualizing the element cost over $T_{m}$ is exactly equivalent to a replacement sinking fund, so membrane replacement is represented by the element life rather than by a separate line. In the baseline, $c_{\mathrm{mem}}$ and $T_{m}$ of Configuration B are single effective values for the ultrafiltration and reverse-osmosis elements together, and Section 4.10 annualizes the two element types separately. In the operating term, $\pi_{E}$ is the electricity price, SEC the specific electrical energy per cubic meter of product, $c_{\mathrm{cip}}$ the cleaning chemical cost, $c_{\mathrm{om}}$ other operation and maintenance, and $c_{b}$ the cost per cubic meter of reverse-osmosis concentrate, of which $\left(1/R_{\mathrm{RO}}-1\right)$ cubic meters arise per cubic meter of product. Ultrafiltration backwash and cleaning water return to the head of the works in both configurations and carry no concentrate charge, so the concentrate term is zero for Configuration A and depends only on the reverse-osmosis stage recovery for Configuration B.

The fouling penalty Φ≥1 multiplies energy and cleaning, representing the elevated transmembrane pressure and cleaning frequency that fouling imposes [15]. Its reference operating state must be stated to avoid double counting. The nominal specific energies and cleaning costs are taken from operating or modeled full-scale trains (Appendix C) and therefore already include routine fouling and cleaning at the conditions of those trains. We define that state as Φ=1. Values Φ>1 represent fouling in excess of the reference state on the local effluent, so the nominal Φ=1.2 is a declared margin for a less favorable feed rather than the total effect of fouling, and the case Φ=1 is the no-double-counting bound reported in Section 4.10. Progressive irreversible fouling within an element’s life is examined there as a scenario. Units are consistent: CRF has dimension yr^−1^ and $c_{\mathrm{cap}}$ dimension USD per (m^3^ yr^−1^), so their product is USD/m^3^, as is every operating term, and BW and $c_{\mathrm{net}}M_{e}\kappa$ are both USD/yr. The fixed cost *F* is a declared constant for the reference zone and does not scale with zone size, so break-even coverages are properties of the zone as well as of the membranes. Section 4.10 evaluates zone sizes from 2.5 to 40 Mm^3^ per year.

With a marginal value *v* of supplied potable water, the annual net benefit and the cost per cubic meter of potable input avoided are given by Equation (8):

(8) $$\mathrm{NB}\left(\kappa \right)=v\;V_{M}\left(\kappa \right)-Z_{M}\left(\kappa \right),\;u_{M}\left(\kappa \right)=\frac{Z_{M}\left(\kappa \right)}{V_{M}\left(\kappa \right)}.$$

Three unit costs answer different questions, and we keep them distinct. The cost per cubic meter of product water is $u_{W}=Z_{M}/W$, the cost per cubic meter placed at the point of use is $u_{P}=Z_{M}/\left[\left(1-\rho_{u}\right)W\right]$, and the cost per cubic meter of potable system input avoided is $u_{M}=Z_{M}/V_{M}$. Only $u_{M}$ is on the same basis as *v*, which values a cubic meter of potable supply avoided, so $u_{M}$ is the unit cost used in the break-even conditions. The costs $u_{W}$ and $u_{P}$ are reported for comparison with supplier and tariff figures.

### 3.6. Decision Frames

Frame 1: incremental cost-effectiveness. A utility does not abolish its tariffs or its pressure-management program when it builds a reclamation plant, so the primary question is whether the scheme added to the status quo repays its cost: NB(κ)≥0, equivalently $u_{M}\left(\kappa \right)\leq v$.

Frame 2: sequencing. The scheme competes for capital and institutional capacity with other programs. Pressure-management expansion, Program P, reduces real losses by a share $\beta_{r}$ and saves $V_{P}=\beta_{r}L_{0}$. A tariff or fixture program, Program T, reduces baseline demand by a net share *r* and saves $V_{T}=rD$. We report both as volumes only and do not rank them against the reuse scheme on a unit-cost axis. The programs deliver different functional outputs: membrane reuse supplies an additional water source of a defined quality, pressure management avoids losses in the existing potable network, and a tariff program reduces demand. Their cost boundaries are also not matched. The pressure-management cost data available to this study exclude network access, valve procurement, calibration, and institutional costs, and the institutional, distributional, and political costs of a tariff program [28] are not monetized. A matched incremental cost-effectiveness comparison would require those costs for the same zone, and none is attempted here.

Because prior expansion changes what remains for the reuse scheme, we evaluate the scheme added after Programs T and P, for which the eligible demand and the effluent stream both scale by (1−r) and the loss multiplier uses the remaining loss share $\lambda_{p}\left(1-\beta_{r}\right)$, as given by Equation (9):

(9) $$V_{M|U}\left(\kappa \right)=\left(1-\rho_{u}\right)\;m_{L|U}\;\psi \left(\kappa ;\theta_{E}\right)\;\left(1-r\right)\;V^{\max},\;m_{L|U}=1+\frac{\eta_{L}\lambda_{p}\left(1-\beta_{r}\right)}{1-\lambda_{p}\left(1-\beta_{r}\right)}.$$

Double counting is avoided by construction: the reuse scheme is credited only with displaced potable demand and the losses avoided in supplying it, and pressure management is credited only with losses avoided in the potable network that remains. This is a conditional sequencing scenario, not a recommendation that one program precede another.

### 3.7. Break-Even Conditions

Substituting Equations (4)–(6) into Equation (8) and collecting terms gives the net benefit in Equation (10):

(10) $$\mathrm{NB}\left(\kappa \right)=K\;\psi \left(\kappa ;\theta_{E}\right)-F-c_{\mathrm{net}}M_{e}\kappa ,\;K=\left[v\left(1-\rho_{u}\right)\;m_{L}-B\right]V^{\max}.$$

Because the loss credit enters only through the multiplier $m_{L}$ of Equation (5), Equation (10) and the thresholds derived from it hold for every $\eta_{L}\in \left[0,1\right]$, consistently with Equation (5). Full credit gives $m_{L}=1/\left(1-\lambda_{p}\right)$, and no credit gives $m_{L}=1$, which is how the results without loss credit in Section 4 are computed.

Four thresholds follow, each answering a separate procurement question. First, the break-even unit treatment cost at a given coverage, the highest *B* the scheme can bear, is obtained by setting NB=0 in Equation (10), which gives Equation (11):

(11) $$B^{*}\left(\kappa \right)=v\left(1-\rho_{u}\right)\;m_{L}-\frac{F+c_{\mathrm{net}}M_{e}\kappa }{\psi \left(\kappa ;\theta_{E}\right)\;V^{\max}}.$$

This is directly comparable with a supplier’s quoted cost per cubic meter of product. Second, the break-even membrane element life $T_{m}^{*}$ solves $B=B^{*}$ for $T_{m}$ with the other inputs fixed, which by Equation (7) reduces to the capital recovery factor condition of Equation (12):

(12) $$\mathrm{CRF}\left(i,T_{m}^{*}\right)=\mathrm{CRF}\left(i,T_{m}\right)+\frac{u\;[B^{*}\left(\kappa \right)-B]}{c_{\mathrm{mem}}},\;T_{m}^{*}=\frac{\ln\;\left[\mathrm{CRF}\left(i,T_{m}^{*}\right)/\left(\mathrm{CRF}\left(i,T_{m}^{*}\right)-i\right)\right]}{\ln(1+i)},$$

which is defined whenever the right-hand side of the first equality exceeds *i*. Otherwise, no element life makes the scheme economic at that coverage.

Third, for untargeted connection order, ψ=κ and the net benefit of Equation (10) is affine in κ, so the break-even coverage has the closed form given in Equation (13):

(13) $$\kappa^{*}=\frac{F}{K-c_{\mathrm{net}}M_{e}},$$

which lies in (0,1] only if $K-c_{\mathrm{net}}M_{e}\geq F$. Otherwise, no coverage is cost-effective. For targeted order with $\theta_{E}>1$ and K>0, NB is strictly concave, has at most two roots in [0,1] and a unique maximizer (Appendix A). We locate a sign change on a grid of step 0.0005 and refine the lower root with Brent’s method. Fourth, the coverage $\kappa^{{}^{\circ}}$ that maximizes net benefit solves the first-order condition of Equation (14),

(14) $$K\;\theta_{E}{\left(1-\kappa \right)}^{\theta_{E}-1}=c_{\mathrm{net}}M_{e}$$

when an interior solution exists, and equals 1 otherwise.

Two further thresholds concern the water value. Because NB is affine in *v* while $V_{M}$ and $Z_{M}$ do not depend on *v*, the configuration crossover at coverage κ is given in closed form by Equation (15):

(15) $$v^{†}\left(\kappa \right)=\frac{Z_{M,B}\left(\kappa \right)-Z_{M,A}\left(\kappa \right)}{V_{M,B}\left(\kappa \right)-V_{M,A}\left(\kappa \right)},$$

defined when Configuration B avoids more potable input than Configuration A. Above $v^{†}$, Configuration B has the higher net benefit. The break-even water value of a configuration, the lowest *v* at which some coverage is cost-effective, is $v^{*}=\min_{\kappa }u_{M}\left(\kappa \right)$, because NB(κ)≥0 exactly when $u_{M}\left(\kappa \right)\leq v$ (Appendix A). Both are thresholds on the water value, to be compared with whichever value concept the decision-maker uses (Section 3.10).

The limiting cases behave as required. At κ=0, NB=−F<0: a scheme that serves nobody carries its fixed compliance cost and no benefit. At B=0 and $F=c_{\mathrm{net}}=0$, $\mathrm{NB}=v\left(1-\rho_{u}\right)\;m_{L}V^{\max}\psi \geq 0$. As *R* falls below $R^{{}^{\circ}}={\bar{e}}_{\varepsilon }/\phi$, the supply term binds and $V^{\max}$ falls linearly in *R*. Above $R^{{}^{\circ}}$, the volume is independent of *R*, and the recovery of Configuration B acts only through the reverse-osmosis concentrate term of Equation (7). As $T_{m}\to \infty$, $\mathrm{CRF}(i,T_{m})\to i$ and the element charge tends to $i\;c_{\mathrm{mem}}/u$ rather than to zero, as a perpetual asset should. Derivations are given in Appendix A.

### 3.8. Declared Parameters and Distributions

Table 3 lists the inputs and classifies each one. No distribution is fitted to data from the zone of interest, and no formal expert-elicitation protocol was used. Membrane recovery, specific energy, element life, and unit capital costs are literature-derived, with the translation from source to model quantity documented in Appendix C. The effluent return ratio, the eligibility scale, the unplaced-product share, the deployment concentration, the fouling penalty, and the network and fixed costs are author-declared scenario assumptions or planning ranges. Nominal deterministic values are the means of the declared distributions. Because these distributions are not calibrated beliefs, ensemble fractions and sensitivity indices describe the selected scenario design rather than uncertainty for a city. Coverage κ is the utility’s decision and is evaluated ona grid, and the water value *v* takes planning values of 0.5, 1.0, and 2.0 USD/m^3^.

None of the cost ranges are based on Saudi procurement or local quotations. Plant and element capital are generic literature calibrations: they are set so that the nominal unit treatment cost of Configuration B is close to the levelized cost of 0.54 USD/m^3^ reported for a reverse-osmosis-based reuse train, and that of Configuration A lies below the 0.40 USD/m^3^ reported for a carbon-based train without desalting [5], consistent with a simpler ultrafiltration-only train. The fixed cost *F*, network cost $c_{\mathrm{net}}$, cleaning and other operating costs, and the concentrate cost $c_{b}$ are planning assumptions. Table 4 decomposes the nominal unit treatment cost into its components, and Section 4.10 reports the fixed and concentrate costs at which the scheme stops breaking even. The reverse-osmosis stage recovery is declared so that the product $R_{\mathrm{UF}}R_{\mathrm{RO}}$ spans the overall train recovery of 0.60–0.88 used for Configuration B in the literature translation of Appendix C, with an upper bound of 0.90. Scaling by sparingly soluble salts is typically the key barrier to operating reverse osmosis at high recovery: concentrate concentrations rise four- to tenfold at recoveries of 75–90%, and calcium phosphate, which is encountered mainly in water reuse and in the reverse-osmosis treatment of groundwater, limited recovery near 85% in the groundwater system studied by Mangal et al. [36].

The reference service zone has annual baseline potable demand $D=10\;{\mathrm{Mm}}^{3}$. Its sector mix is declared, not measured. Declared annual volumes per connection of 650 m^3^ for residential, 6000 m^3^ for hospitality, 3000 m^3^ for mosques, 20,000 m^3^ for healthcare, 2500 m^3^ for commerce and institutions and 5000 m^3^ for landscaping imply $M_{e}=928$ eligible connection equivalents under Configuration A and 1248 under Configuration B. Volumes and throughput-proportional costs scale with *D*, whereas the fixed cost *F* does not. Four further constructed district scenarios vary sector shares and real-loss levels together (Table 5). These scenarios test sensitivity to bundled district characteristics rather than isolating a sector-mix effect.

### 3.9. Uncertainty Analysis Conditional on Coverage

Monte Carlo scenario ensemble. We draw 200,000 realizations. In each realization, the inputs shared by the two configurations, the first block of Table 3 including the recovery of the common ultrafiltration stage, take a single value that both configurations use, and only the configuration-specific membrane inputs are drawn separately (common random numbers). Every comparison between the configurations, such as the share of realizations in which Configuration B has the higher net benefit, is therefore a paired comparison under the same scenario rather than a comparison between different hypothetical systems. In the baseline ensemble, the inputs are independent. Independence is a computational scenario-design assumption, not a claim about physical or economic dependence, and its consequences are examined below. For each coverage on the grid κ=0.1,…,0.9, each configuration and each water value, we report the fraction of draws with NB<0, and for every draw we compute break-even coverage on a grid of step 0.001. These summaries describe the chosen ensemble. They are not probabilities for any city.

Sobol indices. We compute first-order and total-effect Sobol indices [37] of the net benefit over the k=13 uncertain inputs of Configuration A and the k=14 inputs of Configuration B, conditional on coverage, following established practice for environmental models [35]. A scrambled Sobol sequence of dimension 2k with $N=2^{15}$ rows is split into matrices *A* and *B*, each column is mapped through the inverse distribution function of Table 3, and $A_{B}^{\left(j\right)}$ replaces column *j* of *A* with column *j* of *B*. Each design requires (k+2)N model evaluations, 491,520 for Configuration A and 524,288 for Configuration B. With *f* the net benefit and *m* indexing rows, the Saltelli first-order and Jansen total-effect estimators [38] of Equation (16) are applied at each coverage separately:

(16) $$S_{j}=\frac{\frac{1}{N}\sum_{m=1}^{N}f{\left(B\right)}_{m}\left[f{\left(A_{B}^{\left(j\right)}\right)}_{m}-f{\left(A\right)}_{m}\right]}{\mathrm{Var}(f)},\;S_{Tj}=\frac{\frac{1}{2N}\sum_{m=1}^{N}{\left[f{\left(A\right)}_{m}-f{\left(A_{B}^{\left(j\right)}\right)}_{m}\right]}^{2}}{\mathrm{Var}(f)}.$$

Reported 2.5th to 97.5th percentile ranges come from 1000 row bootstraps. Because the design is a scrambled low-discrepancy sequence, these are descriptive stability diagnostics rather than confidence intervals with repeated-sampling coverage. Stabilization is assessed from the sum of first-order indices and from estimates on successive prefixes $2^{10},\ldots ,2^{15}$ of the same scramble (Appendix B).

Descriptive scenario discovery. Following the scenario-discovery approach [34], we search the same ensemble at κ=0.5 for the two-input box with one-sided thresholds having the highest density of negative-net-benefit draws among boxes containing at least half of them. Thresholds span the 5th to 95th percentiles in five-percentile steps over eight candidate inputs. The search is run at v=0.5 USD/m^3^, the regime in which negative net benefit is common. At 1 USD/m^3^, the negative share is too small for a descriptive box to be informative. The box is selected and summarized on the same ensemble and is therefore an exploratory description rather than a validated classification rule.

Dependent inputs. An unfavorable effluent can simultaneously lower recovery, raise the fouling penalty and the specific energy, and shorten element life, and it may also raise plant and element capital through more pretreatment and more membrane area. We represent this coupling by a declared rank-correlation structure along a single adverse-feed direction. In the operating variant, it links $R_{\mathrm{UF}}$, $R_{\mathrm{RO}}$, Φ and the SEC and $T_{m}$ of both configurations, and the operating-and-cost variant adds $c_{\mathrm{cap}}$ and $c_{\mathrm{mem}}$ of both configurations. Recovery and element life correlate negatively with the other members, and each pair has Spearman rank correlation $\pm \rho_{S}$, with $\rho_{S}=0.3$, 0.6 and 0.9. The last is close to the comonotone bound for this direction of coupling. In the Monte Carlo ensemble, the rank correlations are induced with the method of Iman and Conover [39], which preserves every marginal distribution exactly. Variance-based indices of individual inputs lose their usual interpretation when inputs are dependent, so for the coupled set we report multidimensional (group) indices, which remain defined because the coupled set is independent of all other inputs [40]. They are estimated with the estimators of Equation (16), replacing the whole group at once and sampling it through a Gaussian copula with normal-score correlation $2\sin(\pi \rho_{S}/6)$, which gives Spearman correlation $\rho_{S}$. For comparison, we report the group index of the three placement inputs ε, $\rho_{u}$, and $\theta_{E}$ estimated in the same way.

Decision metrics. To interpret the configuration choice under uncertainty, we report, for the paired draws at κ=0.5 and for water values from 0.4 to 2.5 USD/m^3^, the share of draws in which each configuration has positive net benefit, the share in which Configuration B outperforms Configuration A, the expected regret of each choice relative to the best of no scheme, Configuration A and Configuration B in each draw, and the expected value of perfect information (EVPI). The expected value of partial perfect information (EVPPI) for groups of inputs is estimated with the nonparametric regression approach of Strong et al. [41], using tensor-product cubic regression splines on rank-transformed group inputs. We also report the probability that some coverage is cost-effective, $P(v^{*}\leq v)$, and the distribution of the crossover value of Equation (15) over the paired draws.

Structural and scenario analyses. Further analyses vary assumptions that the baseline distributions do not represent. Their declared settings are listed in Appendix D. They cover reuse eligibility (discrete regulatory scenarios and sector-specific multipliers), connection order (an alternative power reach form $\psi =\kappa^{\tau }$ matched to Equation (3) at 20% coverage, and partially targeted order), the counting of eligible connections, network-cost structure, zone size, discrete treatment trains, effluent access of a partially built scheme, the loss credit $\eta_{L}$, concentrate management up to zero-liquid-discharge-level costs, progressive fouling with shortened element life, separate ultrafiltration and reverse-osmosis element terms, feed salinity, salt-passage aging linked to the quality class, seasonal placement with storage, compliance failures and reclaimed-network pumping energy.

### 3.10. Water Value and Avoided Energy

The water value *v* is not a market price, and three value concepts must be distinguished. The economic shadow value of potable supply is the long-run marginal cost of the supply that reuse displaces, such as additional desalination and conveyance. It is the value relevant to a planner allocating national resources. The social opportunity cost adds costs not borne by the utility, such as energy-related emissions, and is at least as large. The utility’s financial avoided cost is the cost that the utility’s own accounts would save, which can be much lower than either when production or bulk transfer is subsidized or charged below cost, and which is the value that governs a procurement decision under financial cost-recovery constraints. The planning values of 0.5, 1.0 and 2.0 USD/m^3^ are scenario values motivated by production costs reported for large desalination plants [42] and by long-distance conveyance. Production cost is not necessarily equal to the marginal value of avoided supply, so the crossover $v^{†}$ and the break-even value $v^{*}$ are reported as scenario thresholds. A decision-maker compares them with the value concept relevant to that decision-maker. No local marginal supply cost was available for this study.

The maximum affordable annual cost of the scheme is $vV_{M}$. Specific energy of the displaced supply of 3.0, 4.5 and 6.0 kWh/m^3^ spans modern seawater reverse osmosis [43] to desalination plus inland conveyance. A static lift of *H* meters at pump efficiency $\eta_{p}$ adds $2.725\times 10^{-3}H/\eta_{p}$ kWh/m^3^, or 2.18 kWh/m^3^ for a 600 m lift at 75% efficiency, comparable to the elevation of the Madinah plains above the Red Sea coast [44]. Net avoided energy subtracts the reclamation plant’s own consumption ΦSEC per cubic meter of product and the reclaimed-network pumping energy $e_{d}$ per cubic meter of product distributed. By the same relation, a distribution head of 30 to 80 m at 70% pump efficiency gives $e_{d}$ of 0.12 to 0.31 kWh/m^3^. We report $e_{d}$ from 0 to 0.6 kWh/m^3^, together with the values at which net avoided energy vanishes and at which the energy ranking of the two configurations reverses. Energy links every displaced cubic meter to emissions and operating cost in a desalination-dependent system [45].

### 3.11. Reproducibility and Use of Generative AI

The analysis is a single Python program (Supplementary File S1, analysis.py) that regenerates every number, table entry, and figure of Section 4 and Appendix D, Appendix E and Appendix F. It uses a fixed seed of 20260928 and seeded scrambled Sobol sequences, and writes its numerical output, including the software versions found at runtime, to Supplementary File S2 (results.json). The reported results were produced with CPython 3.10.11, NumPy 1.26.2, SciPy 1.11.4, and Matplotlib 3.8.2, and the complete environment, including transitive dependencies, is pinned in requirements-lock.txt (Supplementary File S4). Supplementary File S3 (verify_results.py) re-derives the closed-form results from the nominal inputs stored in results.json without importing the analysis code: the unit treatment cost, volumes, net benefit, break-even treatment cost and element life, the closed-form untargeted break-even coverage, the crossover values, the break-even water values, the energy balance and the stepped-wedge minimum detectable effects. It also re-checks the stored Monte Carlo ensemble statistics independently. It draws its own 200,000 paired realizations from the distributions of Table 3 with a different seed and requires each stored statistic at 50% coverage to lie within four Monte Carlo standard errors of its own estimate. These statistics are the shares of draws with negative net benefit, with a binding supply term and with Configuration B ahead of Configuration A, and the median saving, unit treatment cost, and cost per cubic meter avoided. All 64 checks pass for the submitted results.json. Because every random stream of analysis.py is seeded, re-running it in the pinned environment reproduces the Monte Carlo and Sobol statistics of results.json exactly. Figure 1 is drawn in TikZ within the manuscript source.

Generative AI (Claude Opus 5, Anthropic) was used in preparing the original submission to draft and review analysis code, check internal numerical consistency, and edit language, and the authors reviewed that code and manuscript. During revision, Claude Opus 5.5 (Anthropic) was used to implement the additional analyses in analysis.py, to write verify_results.py and the code that draws Figure 1, to revise the text, and to draft the response to the reviewers. The authors checked every equation of Section 3 and Appendix A by hand, reviewed analysis.py and verify_results.py line by line, and reproduced all reported numbers by re-running both scripts in the pinned environment. The authors remain responsible for all reported results.

## 4. Results

Status of reported values. Every number in this section is an output of the model of Section 3 evaluated at the declared inputs of Table 3. None is a measurement made in this study. Unless stated otherwise, results correspond to the reference district mix, targeted connection order, 50% coverage of eligible connections, full loss credit ($\eta_{L}=1$) and v=1 USD/m^3^, and ensemble statistics refer to the 200,000 paired realizations.

### 4.1. Break-Even Conditions at the Nominal Parameter Set

Table 6 reports the nominal outputs. At ε=1, the reuse-eligible demand is $1.745\;{\mathrm{Mm}}^{3}$/yr under Configuration A and $3.085\;{\mathrm{Mm}}^{3}$/yr under Configuration B, against an available product stream RϕD of 5.52 and $4.50\;{\mathrm{Mm}}^{3}$/yr. Approved demand therefore binds in both cases, and the recovery at which the effluent supply would instead bind is $R^{{}^{\circ}}=29.1\%$ for A and 51.4% for B, well below the nominal overall recoveries of 92% and 75%.

With half of the eligible connections served, the reach is ψ=0.750. Configuration A produces $1.309\;{\mathrm{Mm}}^{3}$/yr of product water and avoids $1.355\;{\mathrm{Mm}}^{3}$/yr of potable system input, 11.52 percentage points, at an annualized cost of 648,600 USD, that is, 0.48 USD per cubic meter of potable input avoided. Configuration B produces $2.314\;{\mathrm{Mm}}^{3}$/yr and avoids $2.395\;{\mathrm{Mm}}^{3}$/yr, 20.36 points, at 1,669,100 USD and 0.70 USD/m^3^. At 1 USD/m^3^, both are economic, with net benefits of 706,000 and 726,000 USD per year. Per cubic meter of product water, the costs are 0.50 and 0.72 USD, and per cubic meter placed at the point of use, 0.56 and 0.82 USD (Table 6). These figures, not $u_{M}$, are the ones to compare with supplier prices and tariffs.

The threshold calculations answer separate procurement questions. The break-even unit treatment cost of Equation (11) is 0.76 USD/m^3^ for Configuration A against a modeled 0.22, and 0.87 against a modeled 0.56 for Configuration B. A supplier quotation above those values would fail the screen at this coverage. The break-even element life of Equation (12) is 0.38 years for Configuration A and 1.23 years for Configuration B, far below any reported service life, so element replacement is not the binding constraint in either configuration at the nominal set. Section 4.10 tests this under progressive fouling and salt-passage aging. Break-even coverage is 11.7% and 11.5%, and modeled net benefit is maximized near full deployment at 96.1% and 94.9%.

Charging concentrate on the reverse-osmosis stage alone matters. Charging it on the overall train recovery instead would count ultrafiltration backwash as concentrate: 1/R−1=0.334 m^3^ would arise per cubic meter of product, against $1/R_{\mathrm{RO}}-1=0.227$ m^3^ on the reverse-osmosis stage. The stage-specific charge lowers the unit treatment cost of Configuration B from 0.591 to 0.559 USD/m^3^ and raises its net benefit from 652,000 to 726,000 USD per year. Configuration A is unaffected.

Connection order, water value, and loss credit all change the verdict. Under untargeted order, the same coverage reaches only half of the eligible demand, and break-even coverage rises to 23.0% and 22.8%, values that the closed form Equation (13) reproduces exactly. At 0.5 USD/m^3^, Configuration B is uneconomic at every coverage, because its break-even treatment cost of 0.356 USD/m^3^ falls below its modeled 0.559, whereas Configuration A still breaks even above 43.4% coverage. At 2 USD/m^3^, both configurations break even below 5% coverage. Without loss credit, the avoided input falls by 15%, the cost per cubic meter avoided rises to 0.56 and 0.82 USD/m^3^, and break-even coverage rises to 14.9% and 18.2%. Configuration B is affected more because its cost premium must be recovered from a smaller credited volume.

Table 4 decomposes the nominal unit treatment cost. Plant capital is the largest component in both configurations, followed by the membrane elements, other operation and maintenance, and, for Configuration B, concentrate management. Energy is 6.5% of the unit treatment cost of Configuration A and 11.0% of that of Configuration B, and concentrate management is 12.2% of that of Configuration B. Because net benefit is linear in *F* and in $c_{b}$, the scheme at 50% coverage and 1 USD/m^3^ breaks even up to a fixed cost of F+NB, 1006 and 1026 kUSD per year for Configurations A and B, and Configuration B breaks even up to a concentrate cost of 1.69 USD per cubic meter of concentrate (Section 4.10).

**Figure 3:**
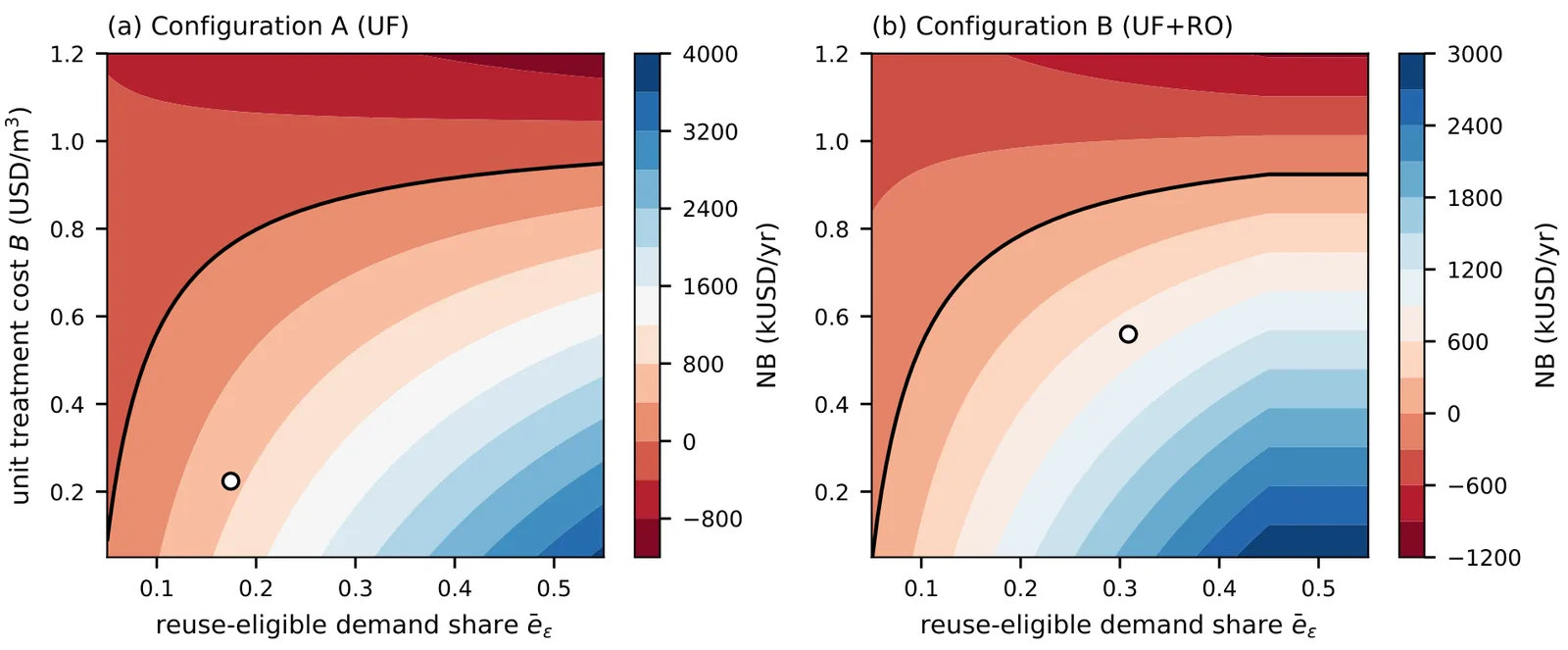
Annual net benefit at κ=0.50 and v=1 USD/m^3^ across the reuse-eligible demand share ${\bar{e}}_{\varepsilon }$ and the unit treatment cost *B*, for (**a**) Configuration A and (**b**) Configuration B. The solid black contour is break-even (NB=0), and the open circle is the nominal operating point of each configuration.

**Figure 4:**
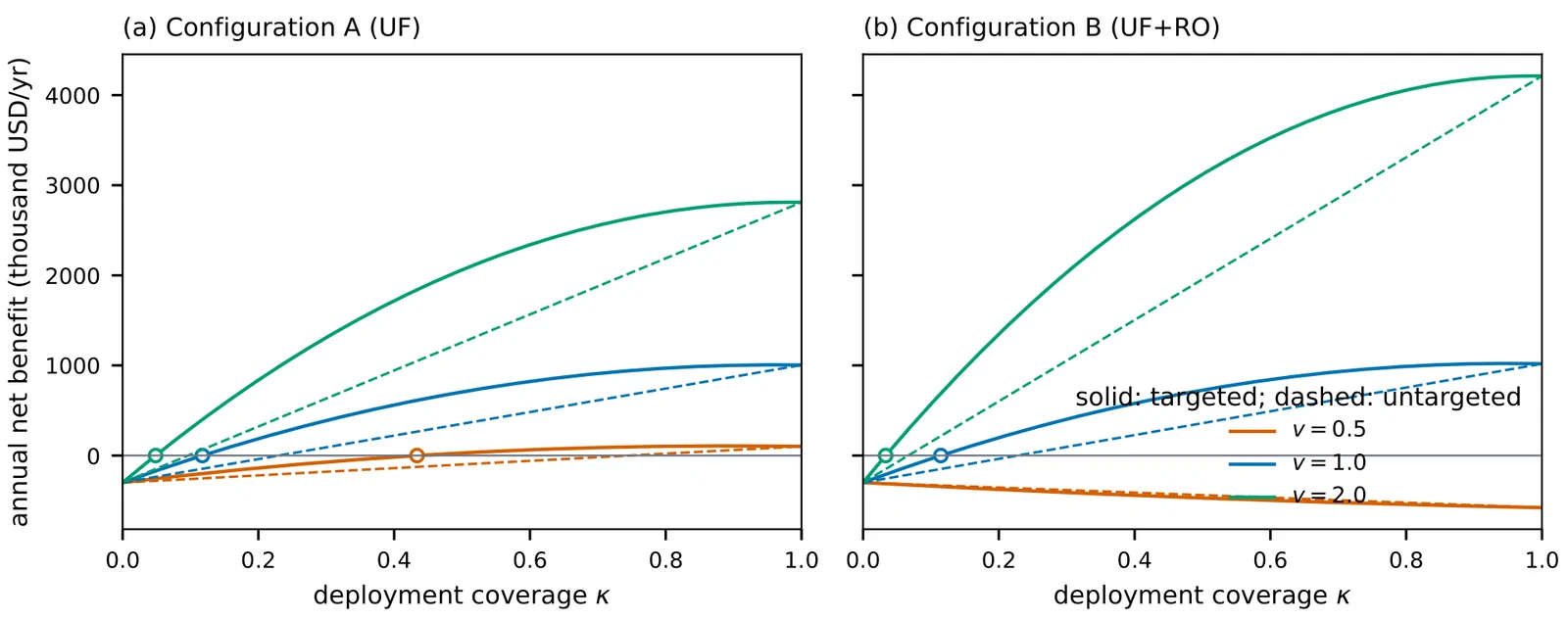
Annual net benefit against deployment coverage for (**a**) Configuration A and (**b**) Configuration B, at water values of 0.5, 1, and 2 USD/m^3^ and the nominal parameter set. Solid curves are targeted connection order and dashed curves untargeted order. Open circles mark the break-even coverage $\kappa^{*}$ under targeted order.

Figure 3 shows net benefit over the reuse-eligible demand share and the unit treatment cost. The break-even contour is a straight line because the net benefit is affine in both quantities, and the nominal operating point sits well inside the economic region for both configurations. Figure 4 shows net benefit against coverage: it is concave under targeted order, with a break-even coverage, an interior maximum, and a range near full deployment over which additional connections cost more than the water they place, and linear under untargeted order.

### 4.2. Which Membrane Configuration, and When

**Figure 5:**
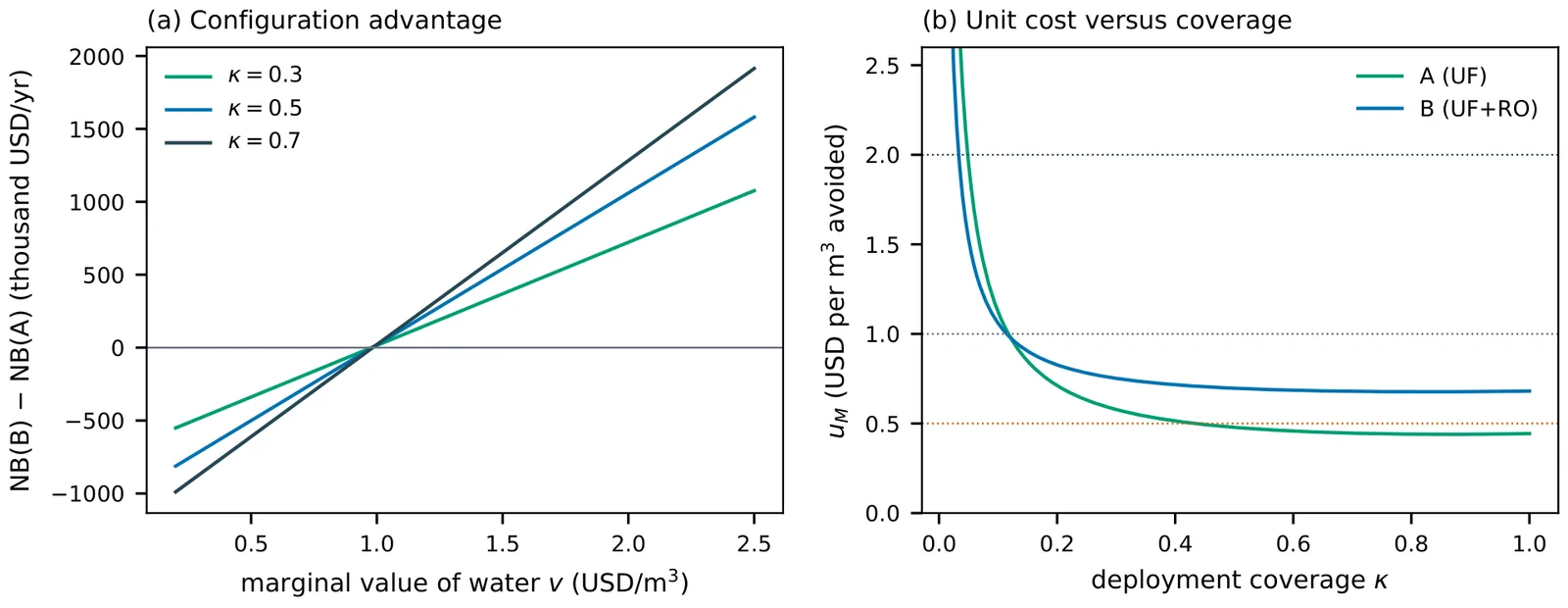
(**a**) Difference in annual net benefit between Configuration B and Configuration A against the marginal value of water, at three coverage levels and the nominal parameter set. The zero crossing is the crossover value $v^{†}$. (**b**) Cost per cubic meter of potable input avoided, $u_{M}$, against coverage for both configurations, with dotted lines at water values of 0.5, 1 and 2 USD/m^3^. The minimum of each curve is the break-even water value $v^{*}$.

Configuration B avoids 1.77 times the potable input of Configuration A but costs 2.57 times as much, so the two are separated by a crossover in the marginal value of water. By Equation (15), the crossover at the nominal parameter set is $v^{†}=0.981$ USD/m^3^ at 50% coverage. It is almost invariant to coverage, at 0.979 and 0.984 USD/m^3^ for 30% and 70% coverage, because both configurations scale their volume and their throughput-proportional cost through the same reach function. What differs between them is the ratio of value placed to cost incurred per cubic meter of product, which does not depend on κ. Below $v^{†}$, the ultrafiltration-only scheme is the better investment despite avoiding less potable input. Above it, the additional eligible demand that desalting unlocks repays the reverse-osmosis stage. Without loss credit, the crossover rises to 1.154 USD/m^3^, because each cubic meter of product then avoids less potable input while the cost premium of desalting is unchanged. Charging concentrate on the overall train recovery would raise the nominal crossover to 1.052 USD/m^3^. Figure 5 shows the advantage curve and the cost per cubic meter avoided against coverage. The crossover is a scenario threshold: whether a utility lies above or below it depends on which value concept applies to it (Section 3.10) and on local marginal supply costs, which were not available to this study.

Interpreting the crossover under uncertainty. The crossover is a threshold at the nominal point, whereas the share of draws in which Configuration B has the higher net benefit is a property of the whole ensemble, and the two answer different questions. Over the paired draws, Configuration B has the higher net benefit in 0.0% of draws at 0.5 USD/m^3^, 46.2% at 1 USD/m^3^ and 99.99% at 2 USD/m^3^. Applied to each paired draw, Equation (15) gives a distribution of the crossover with median 1.015 USD/m^3^ and 5th to 95th percentiles of 0.778 to 1.304 USD/m^3^. A share near one half at 1 USD/m^3^ therefore means that this water value lies inside the distribution of the crossover, not that the configurations are equivalent. Pairing matters for this comparison. Drawing the two configurations from independent random streams would change the share at 1 USD/m^3^ only slightly, to 47.5%, but would lower the share at 2 USD/m^3^ to 88.3%, because independent streams compare different scenarios rather than the two technologies under the same scenario.

**Figure 6:**
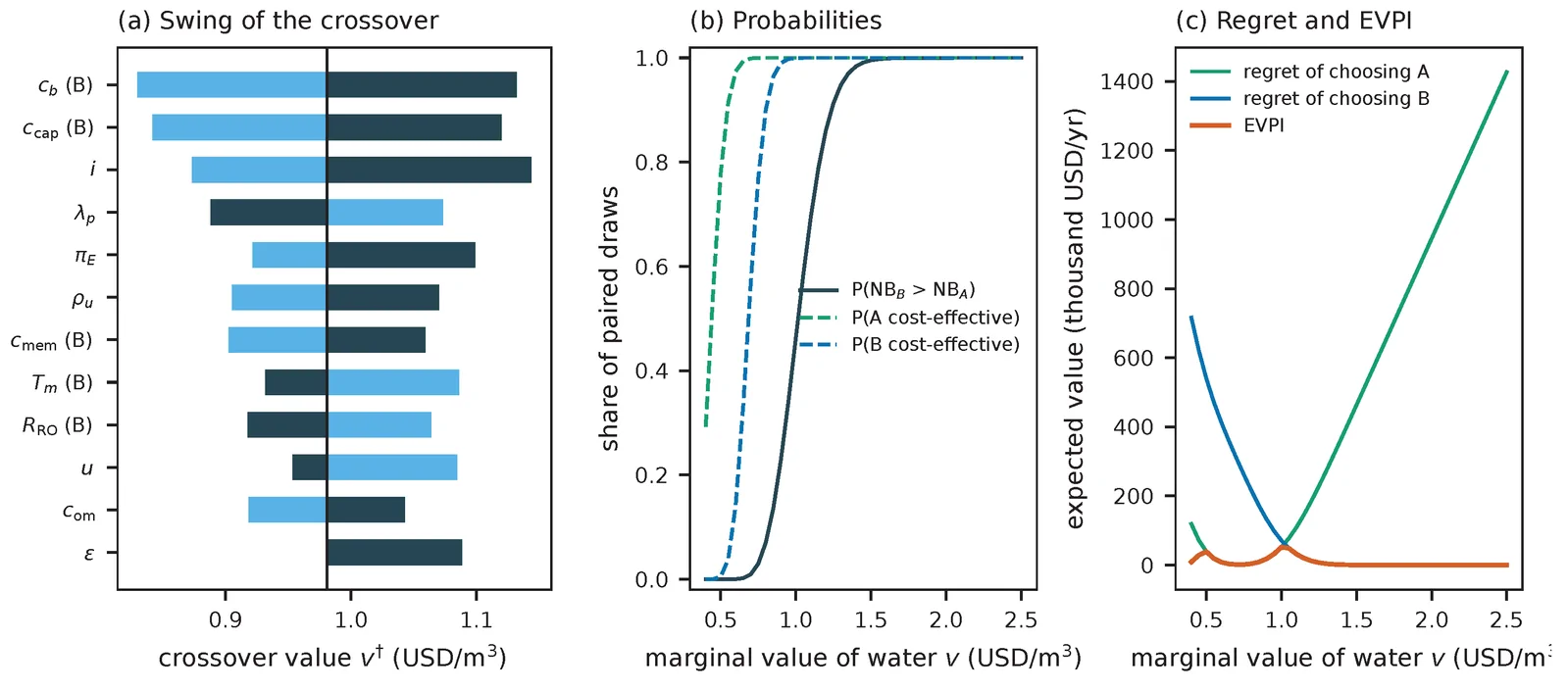
(**a**) One-at-a-time swing of the crossover value $v^{†}$ at κ=0.5 for the twelve inputs that move it most, with light bars for the low setting and dark bars for the high setting (Table A5). An input of one configuration only is marked (A) or (B). (**b**) Share of paired draws in which Configuration B has the higher net benefit at κ=0.5, and probability that some coverage is cost-effective for each configuration, against the water value. (**c**) Expected regret of choosing each configuration and the expected value of perfect information (EVPI) at κ=0.5.

Table 7 and Figure 6b,c express the choice in decision-theoretic terms. At 1 USD/m^3^, the expected net benefits of the two configurations are almost equal, 693,000 USD per year for Configuration A and 674,000 USD for Configuration B, so the choice that maximizes expected net benefit is Configuration A by a small margin. The expected regret of choosing Configuration A is 52,000 USD per year and that of choosing Configuration B is 71,000 USD. The expected value of perfect information has two peaks: 52,000 USD per year at 1 USD/m^3^, near the crossover, about 8% of the expected net benefit, and 39,000 USD at 0.5 USD/m^3^, near the break-even water value of Configuration A, where the open question is whether to build at all. Between and beyond these peaks, it is small, about 1800 and 7000 USD at 0.75 and 1.25 USD/m^3^. The partial values show what information is worth acquiring for the configuration choice: at 1 USD/m^3^, perfect information on the unit treatment costs of the two trains would be worth 43,000 USD per year, most of the EVPI. Perfect information on plant capital alone would be worth 31,000 USD and on the membrane-specific costs alone 27,000 USD (partial values are not additive), against 15,000 USD for placement, 14,000 USD for real losses and almost nothing for the fixed and network costs, which affect both configurations alike. For the configuration choice near the crossover, a firm quotation for both trains is therefore worth more than refined placement data, whereas whether to build at all depends mainly on placement, real losses, and, near the break-even water value, the fixed cost (Section 4.6).

The crossover also varies with district composition, from 0.852 USD/m^3^ in the healthcare and institutional scenario, where little demand is eligible without desalting, but cooling-tower makeup is large, to 1.386 USD/m^3^ in the landscaped scenario, where restricted-access irrigation can accept ultrafiltered water directly (Section 4.8).

### 4.3. Conditions That Decide Feasibility and the Configuration Choice

Figure 6a and Table A5 (Appendix D) move each input one at a time from the 5th to the 95th percentile of its declared distribution, and each deterministic constant over a declared range, with all other inputs at their nominal values. Two different sets of conditions emerge.

Whether a scheme pays at all is decided mainly by placement and scale. Break-even coverage of Configuration A moves from 25.4% to 5.7% as zone demand rises from 5 to 20 Mm^3^ per year, from 6.3% to 17.6% across the fixed-cost range, from 18.4% to 8.6% with the concentration of eligible demand, from 15.6% to 9.4% with the eligibility scale, and from 13.4% to 10.2% with the real-loss share. Configuration B responds similarly. The membrane inputs of Configuration A each move it by less than one percentage point. Those of Configuration B move it more, by up to 3.8 points for concentrate cost and 3.5 points for plant capital, and by about 2 points for element life, element capital, and reverse-osmosis recovery. The financing conditions matter for both: a discount rate of 3% or 10% instead of 6% moves the break-even coverage of Configuration B between 9.9% and 14.9%.

Which configuration to choose is decided by a different set of conditions that fix the cost premium of desalting. The crossover $v^{†}$ responds most to the concentrate cost, from 0.83 to 1.13 USD/m^3^ as $c_{b}$ rises from 0 to 0.60 USD/m^3^, to the plant capital of Configuration B (0.84 to 1.12), the discount rate (0.87 to 1.14 for 3% to 10%), the real-loss share (1.07 to 0.89), the electricity price (0.92 to 1.10 for 0.03 to 0.12 USD/kWh), the unplaced share (0.91 to 1.07), and the element capital, element life and recovery of the reverse-osmosis train (0.90 to 1.06, 1.09 to 0.93 and 1.06 to 0.92). It does not respond to zone size, the fixed cost, the effluent return ratio, or the ultrafiltration-stage recovery, which affect both configurations alike, and it responds only weakly to the concentration of eligible demand. The two conditions that act through the eligible demand that desalting adds move it further still (Section 4.10): feed salinity, which lowers the crossover to 0.80–0.84 USD/m^3^ at 2500–3500 mg/L and removes any advantage of reverse osmosis below 450 mg/L, and the regulatory regime, which moves it from 0.85 to 1.16 USD/m^3^ between the restrictive and permissive eligibility scenarios.

The break-even water value, the lowest value at which some coverage is cost-effective, is 0.44 USD/m^3^ for Configuration A and 0.68 USD/m^3^ for Configuration B at the nominal set, and 0.52 and 0.80 USD/m^3^ without loss credit. Across the paired ensemble, its median is 0.44 and 0.69 USD/m^3^, with 5th to 95th percentiles of 0.34–0.57 and 0.56–0.83 USD/m^3^. These thresholds answer the financial-accounting question directly. At the nominal set, a utility whose financial avoided cost is below about 0.44 USD/m^3^ cannot repay either scheme from its own accounts. A utility whose avoided cost lies between 0.44 and 0.68 USD/m^3^ can repay only the ultrafiltration scheme, and the reverse-osmosis scheme becomes the better financial investment only above the crossover of 0.98 USD/m^3^. Where the planner’s shadow value exceeds these thresholds but the utility’s financial avoided cost does not, the scheme is economically justified but needs a transfer to be financially viable, and a financially constrained utility would choose Configuration A over a wider range of conditions than the economic analysis alone suggests.

### 4.4. What Limits the Recoverable Volume

In 98.8% of declared draws for Configuration B, and in every draw for Configuration A, the recoverable volume is limited by approved reuse demand rather than by the membrane supply term RϕD. Membrane water recovery therefore enters the economics of Configuration B almost entirely through the concentrate term of Equation (7) rather than through the volume placed. This result is conditional, not general. It holds because, under the declared eligibility weights and district mix, approved demand is a minority of potable demand, so the binding condition of Section 3.4, $R\phi <{\bar{e}}_{\varepsilon }$, is rarely met. At the nominal recoveries and effluent return ratio, that condition requires approved demand above 55.2% of potable demand for Configuration A and above 45.0% for Configuration B. Among the constructed cases, it is met in the landscaped district under Configuration B, where eligible demand reaches 58.7% of potable demand, in 12.2% of draws under the permissive eligibility scenario, and in 0.01% under the restrictive one. Product-water placement rather than recovery governs the recoverable volume under these conditions. In a zone where approved demand exceeds these shares, or where the effluent return ratio falls below about 0.41 for Configuration B, the supply term binds and recovery buys volume.

Where the supply term binds, the proportional expansion of Equation (4) understates the deliverable volume relative to a plant that draws on the whole collected effluent from the first phase (Section 3.4). At the reference mix, this full-access variant leaves every nominal result unchanged. It raises the product water of Configuration B at 50% coverage in 1.2% of the paired draws, by a median of 4.2% in those draws, leaves the share of draws with negative net benefit at 0.29% and raises the share in which Configuration B outperforms Configuration A at 1 USD/m^3^ from 46.2% to 46.4%. Under the permissive eligibility scenario, the nominal results are again unchanged, and that share rises from 12.9% to 13.4%. The assumption matters only where supply binds at the nominal inputs, which among the constructed cases is the landscaped district under Configuration B (Section 4.8).

The fixed cost is what makes small deployments expensive per cubic meter and is the reason no coverage below about 11.5% is cost-effective at 1 USD/m^3^ in the reference zone. Table 4 gives the components of the nominal annualized cost.

### 4.5. Negative-Net-Benefit Share and Break-Even Coverage in the Declared Ensemble

**Figure 7:**
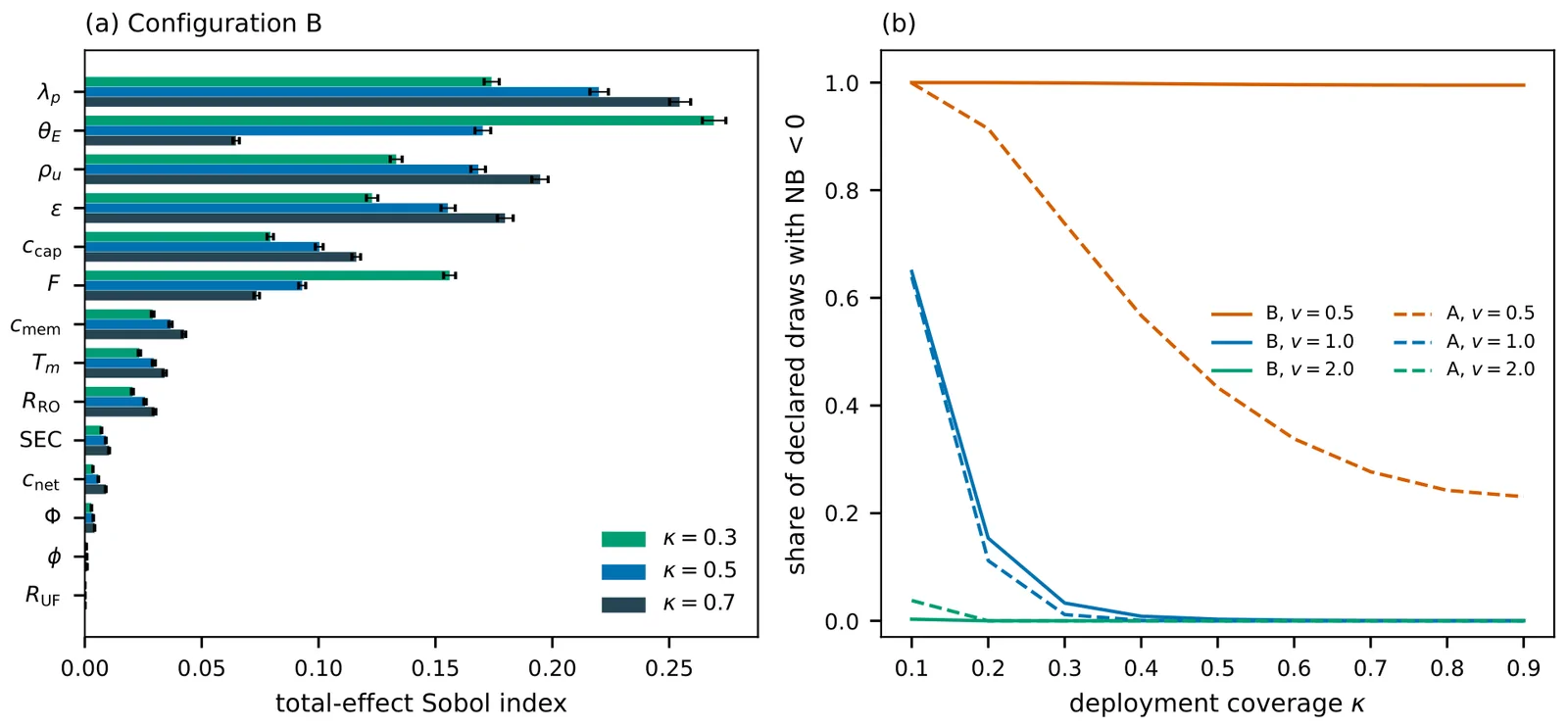
(**a**) Total-effect Sobol indices of annual net benefit for Configuration B at κ=0.3, 0.5 and 0.7 within the declared independent scenario design (v=1 USD/m^3^, targeted order). Bars are row-bootstrap stability ranges, not inferential confidence intervals. (**b**) Share of the 200,000 paired draws with negative net benefit, conditional on coverage, for Configuration B (solid) and Configuration A (dashed) at three water values.

Figure 7b reports the share of the paired draws with negative net benefit conditional on coverage. At 1 USD/m^3^ and targeted order, the share for Configuration B falls from 64.9% at 10% coverage to 15.4% at 20%, 3.3% at 30%, 0.29% at 50% and 0.08% at 70%. For Configuration A, it falls from 63.9% at 10% coverage to 11.2% at 20% and 1.2% at 30%, and is below 0.01% from 50% upward. At 0.5 USD/m^3^, the picture reverses: at least 99.5% of draws are negative for Configuration B at every evaluated coverage, while for Configuration A the share falls from 99.9% at 10% coverage to 43.3% at 50% and 23.1% at 90%. At 2 USD/m^3^, no draw is negative for either configuration from 30% coverage upward.

Break-even coverage varies correspondingly. At 1 USD/m^3^, some coverage is cost-effective in 100% of Configuration A draws, with median break-even coverage 11.9% and a 5th to 95th percentile range of 5.6% to 23.8%, and in 99.95% of Configuration B draws, with median 12.2% and range 5.5% to 27.2%. At 0.5 USD/m^3^, some coverage is cost-effective in 78.0% of Configuration A draws, with median 37.3% and range 16.6% to 74.2%, but in only 0.5% of Configuration B draws.

At 50% coverage, the median modeled saving is 11.28 percentage points of system input for Configuration A, with a 5th to 95th percentile range of 8.10 to 14.78, and 19.59 points for Configuration B, with a range of 14.29 to 24.46. The median cost per cubic meter of potable input avoided is 0.48 USD/m^3^, range 0.36 to 0.67, for Configuration A and 0.71 USD/m^3^, range 0.57 to 0.87, for Configuration B. Median unit treatment cost is 0.225 and 0.564 USD/m^3^ against median break-even treatment costs of 0.757 and 0.864 USD/m^3^ at 1 USD/m^3^.

### 4.6. Global Sensitivity Conditional on Coverage

Table 8 and Figure 7a report Sobol indices within the declared independent scenario design. For Configuration B at 50% coverage, the largest total-effect indices belong to the real-loss share $\lambda_{p}$ (0.220), the eligible-demand concentration $\theta_{E}$ (0.170), the unplaced-product share $\rho_{u}$ (0.168) and the eligibility scale ε (0.155), followed by plant capital $c_{\mathrm{cap}}$ (0.100) and fixed cost *F* (0.093). The membrane-specific inputs follow: element capital $c_{\mathrm{mem}}$ (0.037), element life $T_{m}$ (0.030), reverse-osmosis recovery $R_{\mathrm{RO}}$ (0.026), specific energy SEC (0.009) and the fouling penalty Φ (0.004), together about 0.10 of the total effect. The ultrafiltration-stage recovery and the effluent return ratio ϕ have total effects of 0.000 and 0.001, because supply almost never binds. The reverse-osmosis recovery acts only through the concentrate term, which depends on the stage recovery rather than on the overall train recovery.

The ranking shifts with coverage in an interpretable way. At 30% coverage, the concentration $\theta_{E}$ leads (0.269) and fixed cost rises to 0.156, because the cost is spread over fewer connections and the order in which they are connected matters most early. At 70% coverage, the concentration falls to 0.065 while the eligibility scale rises to 0.180 and the unplaced share to 0.195, because most eligible demand is already reached and the remaining question is how much of it exists and can be placed. First-order indices sum to 0.979–0.986, so interactions are modest within this design.

For Configuration A, the picture is simpler: at 50% coverage and 1 USD/m^3^, the eligibility scale (0.380) and concentration (0.260) dominate, ahead of fixed cost (0.146), real losses (0.117) and the unplaced share (0.090), while every membrane-specific input has a total effect below 0.002 and the ultrafiltration recovery has an index indistinguishable from zero. At 0.5 USD/m^3^, the regime in which Configuration A can fail, fixed cost dominates (0.488) ahead of the eligibility scale (0.169) and concentration (0.115).

Dependent inputs. Table 9 reports how these conclusions change when membrane operating and cost parameters are coupled along an adverse-feed direction. For Configuration A, nothing material changes. The coupled group’s total-effect index stays at or below 0.004 under operating coupling, and 0.040 when costs are coupled as well, against 0.70–0.72 for the placement group, and the scenario-discovery box at 0.5 USD/m^3^ is unchanged (Section 4.7). For Configuration B, the decision statistics move moderately. At 50% coverage and 1 USD/m^3^, the share of draws with negative net benefit rises from 0.29% to 0.76% under operating coupling at $\rho_{S}=0.9$ and to 3.1% when costs are coupled as well. At 30% coverage, it rises from 3.3% to 4.4% and 8.7%. The share in which Configuration B outperforms Configuration A rises from 46.2% to 47.5% and 49.0%, and median break-even coverage stays at 12.2%. The variance attribution moves more. Under operating coupling, the group index of the five coupled membrane inputs rises from 0.068 to 0.113, 0.154, and 0.190 at $\rho_{S}=0.3$, 0.6, and 0.9, while that of the placement group falls from 0.488 to 0.426, so placement still dominates. When costs are coupled as well, the treatment group rises from 0.203 to 0.357, 0.461 and 0.535 and overtakes the placement group, which falls to 0.397, 0.336 and 0.293, at a rank correlation between 0.3 and 0.6. The conclusion that membrane-specific parameters are secondary therefore holds for Configuration A. For Configuration B, it holds under independence and under coupling of the operating parameters alone, but not if an adverse feed also raises plant and element capital. Which regime applies is an empirical question that only joint measurements on local effluent can answer.

### 4.7. Where Negative-Net-Benefit Draws Concentrate

At 50% coverage and 0.5 USD/m^3^, 43.3% of Configuration A draws have negative net benefit. The descriptive search selects the box ε<1.055 and F>329,700 USD/yr. It contains 26.0% of all draws and 52.2% of the negative draws, and 87.0% of draws inside it are negative. The combination is a low eligibility scale with a high fixed compliance and monitoring cost: a district where relatively little demand can legally accept reclaimed water and where the standing cost of running a certified scheme is high. Both can be measured in Stage 0, which is a favorable property for a screening gate. The same box is selected when the membrane operating parameters are coupled at rank correlations up to 0.9, with its density and coverage changing by less than 0.1 percentage points. These density and coverage values are in-sample summaries of a selected box, not out-of-sample performance. For Configuration B at 1 USD/m^3^, negative draws remain too rare for a descriptive box even under coupling.

**Figure 8:**
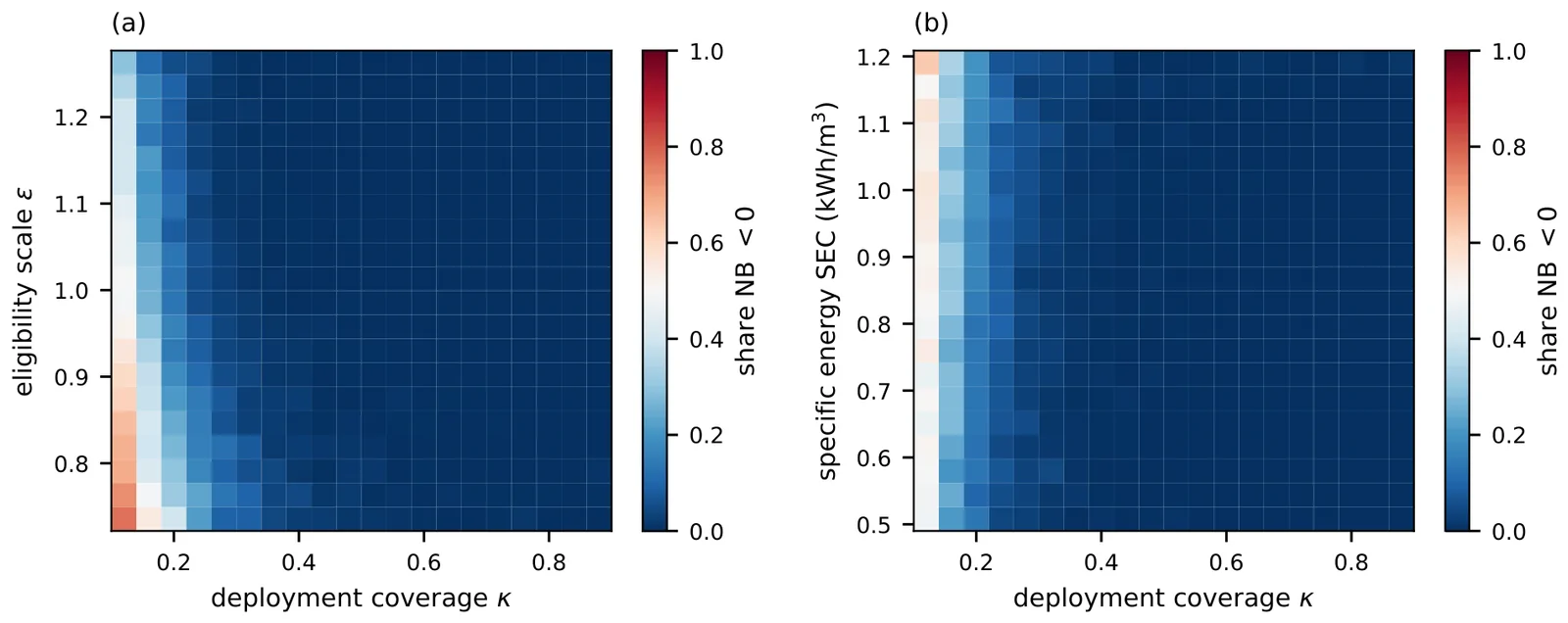
Share of declared Configuration B draws with negative net benefit (v=1 USD/m^3^, targeted order), conditional on coverage and (**a**) the reuse-eligibility scale ε or (**b**) the specific energy of the membrane train. The exploration design uses coverage uniform on [0.1,0.9] and 200,000 independent parameter draws. Bins with fewer than 30 draws are blank.

Figure 8 maps the conditional share of negative draws over coverage against the eligibility scale and against specific energy. The eligibility scale separates outcomes strongly at every coverage, whereas specific energy does not separate them at all over its declared range, which is the same conclusion the Sobol indices reach by a different route.

### 4.8. Sensitivity Across Constructed District Scenarios

Table 10 repeats the analysis for the five constructed scenarios. Because sector shares and real-loss levels change together in these scenarios, the bundled differences cannot be attributed to sector mix alone, so the table also reports the crossover with $\lambda_{p}$ held at 0.15 in every scenario. The landscaped scenario is the most favorable for both configurations, with break-even coverage of 5.5% and 6.7% and costs of 0.34 and 0.62 USD per cubic meter avoided. It is also the only scenario in which the effluent supply binds under Configuration B, so its Configuration B results depend on how the effluent stream is made available to a partial scheme. Table 10 uses the proportional expansion of Equation (4). If the whole collected effluent is available from the first phase (Section 3.4), Configuration B delivers 4.40 rather than 3.37 Mm^3^/yr of product water at 50% coverage. Its saving then rises from 29.69 to 38.71 percentage points, its net benefit from 1426 to 1979 kUSD/yr, and its cost per cubic meter avoided falls from 0.62 to 0.59 USD/m^3^. Its break-even coverage falls from 6.7% to 5.0%, and its optimal coverage from 95.9% to 51.7%, the coverage at which the reach exhausts the effluent supply and beyond which further connections add network cost without volume. The crossover falls from 1.386 to 0.918 USD/m^3^, and Configuration B outperforms Configuration A in 48.0% rather than 18.2% of the draws at 1 USD/m^3^. For this district, the configuration choice therefore depends on whether the treatment plant draws on the whole effluent stream from the start, a design decision that should be fixed before the two configurations are compared. The nominal results of Configuration A are unaffected, because its eligible demand stays below its supply. The healthcare and institutional scenario is the least favorable for Configuration A, whose eligible demand falls to 11.3% of potable demand, and it is the scenario in which Configuration B is the better investment at 1 USD/m^3^ by the clearest margin, with a crossover of 0.852 USD/m^3^. The crossover lies below 1 USD/m^3^ in the reference, central, and healthcare scenarios and above it in the residential and landscaped scenarios, or below it in the landscaped scenario under full effluent access, so the configuration decision is district-specific rather than city-wide.

A one-factor comparison separates the two drivers. With $\lambda_{p}$ fixed at 0.15, composition alone moves the crossover from 0.805 USD/m^3^ in the healthcare scenario to 1.473 USD/m^3^ in the landscaped scenario. With composition fixed at the reference mix, $\lambda_{p}$ alone moves it from 1.096 to 0.865 USD/m^3^ as $\lambda_{p}$ rises from 0.05 to 0.25, because a larger loss credit raises the value of the additional volume that Configuration B places. Composition is therefore the larger driver. The scenario-specific loss levels narrow the bundled range, since the healthcare scenario was given a low and the landscaped scenario a high $\lambda_{p}$. Without loss credit, $\lambda_{p}$ drops out of the comparison, and the crossover at the reference mix is 1.154 USD/m^3^ for every $\lambda_{p}$.

### 4.9. Sequencing and Avoided Energy

Table 11 reports the volumes of the sequencing frame. Pressure-management expansion saves $0.388\;{\mathrm{Mm}}^{3}$ per year, 3.30 percentage points of system input, and a tariff or fixture program $0.600\;{\mathrm{Mm}}^{3}$, 5.10 points, at the nominal responses. Neither is compared with the reuse scheme on a unit-cost axis, for the reasons given in Section 3.6. Their volumes show only that loss control and demand management, which are bounded by the losses and the demand that exist, operate at a different scale from the 1.36 to $2.40\;{\mathrm{Mm}}^{3}$ per year that the reuse configurations avoid at 50% coverage.

**Figure 9:**
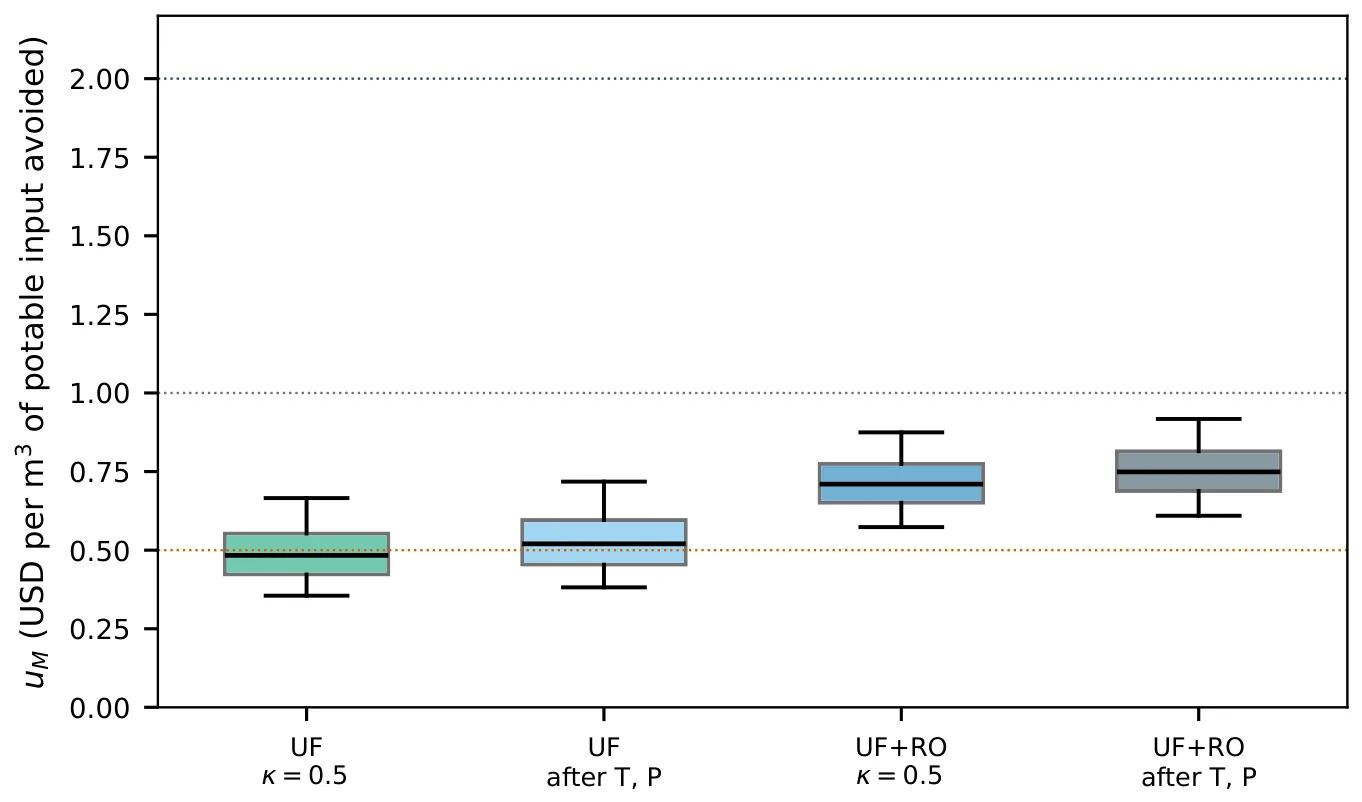
Cost per cubic meter of potable input avoided, $u_{M}$, of the two reuse configurations over the paired draws at κ=0.5, alone and after Programs T and P have expanded. Boxes span the 25th to 75th percentiles, whiskers the 5th to 95th percentiles, and the thick line is the median. Pressure management and the tariff program are not shown because their cost boundaries are not matched with those of the reuse scheme (Section 3.6). Dotted horizontal lines mark water values of 0.5, 1, and 2 USD/m^3^.

In the stipulated sequence where Programs T and P expand first, Configuration A avoids $1.226\;{\mathrm{Mm}}^{3}$ rather than $1.355\;{\mathrm{Mm}}^{3}$, its cost per cubic meter avoided rises from 0.48 to 0.51 USD/m^3^ and its break-even coverage from 11.7% to 13.3%. Configuration B avoids $2.168\;{\mathrm{Mm}}^{3}$ rather than $2.395\;{\mathrm{Mm}}^{3}$, at 0.73 USD/m^3^ and 13.5% break-even coverage. Prior expansion therefore erodes the reuse case modestly, mainly because reducing potable demand reduces both the effluent stream and the eligible demand. Figure 9 shows the distribution of $u_{M}$ over the paired draws for both configurations, alone and after Programs T and P.

Table 12 converts the volumes into the maximum affordable annual cost and into avoided energy. At 1 USD/m^3^, Configuration A can cost up to 1,355,000 USD per year before it stops repaying itself, against a nominal cost of 648,600 USD, and Configuration B up to 2,395,000 against 1,669,100. Net avoided energy is the energy of the displaced potable supply less the reclamation plant’s own consumption, 0.24 kWh/m^3^ of product for Configuration A and 1.02 kWh/m^3^ for Configuration B at the nominal fouling penalty, and less the reclaimed-network pumping energy $e_{d}$. Without pumping, at a displaced specific energy of 4.5 kWh/m^3^, the net avoided energy is 5.78 GWh per year for Configuration A and 8.41 GWh for Configuration B. At 3.0 kWh/m^3^, the figures are 3.75 and 4.82 GWh, because the reverse-osmosis stage consumes a larger share of what it saves. Pumping lowers both: at $e_{d}=0.3$ kWh/m^3^, within the 0.12–0.31 kWh/m^3^ of a 30–80 m distribution head, the net avoided energy at 4.5 kWh/m^3^ is 5.39 and 7.72 GWh per year.

The direction of both conclusions survives reclaimed-network pumping. At a displaced specific energy of 3.0 kWh/m^3^, net avoided energy stays positive unless $e_{d}$ exceeds 2.86 kWh/m^3^ for Configuration A and 2.08 kWh/m^3^ for Configuration B, and Configuration B saves more energy than Configuration A unless $e_{d}$ exceeds 1.07 kWh/m^3^ (2.62 and 4.17 kWh/m^3^ at displaced specific energies of 4.5 and 6.0 kWh/m^3^). Both thresholds lie far above plausible distribution pumping, so the energy ranking of the configurations and the sign of the emissions effect are robust, although their magnitudes shrink. Charging pumping at the electricity price changes the economics little: at $e_{d}=0.4$ kWh/m^3^, the net benefits at 50% coverage fall to 675,000 and 671,000 USD per year and the crossover rises to 1.004 USD/m^3^. We do not claim that any procurement will cost less than the affordability ceilings. We state them so that a utility can compare them with a quotation.

### 4.10. Robustness to Structural Assumptions

Table 13 and Table 14 report the structural and scenario analyses of Section 3.9 for both configurations, with settings in Appendix D. Table 13 covers placement, connection order, network, and scale, and Table 14 covers the membrane process.

Loss credit. Removing the loss credit of Equation (5) remains the most consequential single assumption. At the nominal set, net benefit falls from 706,000 to 503,000 USD per year for Configuration A and from 726,000 to 367,000 USD for Configuration B, and break-even coverage rises to 14.9% and 18.2%. Across the paired draws, the share with negative net benefit at 50% coverage rises to 0.09% for Configuration A and 5.8% for Configuration B, and Configuration B outperforms Configuration A in only 11.9% of draws at 1 USD/m^3^. Half credit, $\eta_{L}=0.5$, lies between the two. A utility that cannot demonstrate that reduced throughput reduces real losses should use the conservative variant, which is why Table 6 reports it beside the baseline.

Eligibility. The regulatory regime shifts both feasibility and the configuration choice. Under the restrictive scenario, in which only outdoor and cooling uses are approved, break-even coverage rises to 18.3% and 14.5% and the crossover falls to 0.85 USD/m^3^, because Configuration A loses more of its eligible demand than Configuration B. Under the permissive scenario, break-even coverage falls to 8.4% and 9.5%, and the crossover rises to 1.16 USD/m^3^, because approval of ultrafiltered water for more uses removes much of the volume advantage of desalting. Replacing the common multiplier ε with independent sector-specific multipliers leaves the ensemble statistics almost unchanged, with negative-net-benefit shares at 50% coverage of <0.01% and 0.20%, median break-even coverages of 11.9% and 12.1%, and Configuration B outperforming Configuration A in 45.8% of draws at 1 USD/m^3^. The six sector multipliers together have a total-effect index of 0.159 for Configuration A and 0.044 for Configuration B, against 0.380 and 0.155 for the common multiplier, and the landscaping multiplier carries most of it for Configuration A (0.115). A common multiplier is therefore the more conservative representation, because it lets errors in every sector compound, whereas sector-by-sector errors partly cancel.

Connection order, connection counting and network structure. If only half of the connections can follow the targeted ranking, break-even coverage rises to 15.8% and 15.5%, and under untargeted order to 23.0% and 22.8%. The targeted result is thus the most favorable case, and a utility constrained by topology, equity, or regulation should plan with the partially targeted or untargeted values. Counting every non-residential user with an eligible use as a whole connection more than doubles the eligible connections of Configuration A, from 928 to 1995, and raises those of Configuration B from 1248 to 2020, but raises break-even coverage only to 12.4% and 11.9%, because the network cost per connection is small relative to the fixed cost. Counting every residential household as a whole connection as well, which would require dual plumbing in every home, raises it to 17.9% and 16.8%. The alternative network-cost structures move break-even coverage within 11.0–14.1%: a trunk main costing half of the full-coverage network and incurred at the first connection raises it to 13.8% and 14.1%, while convex and concave cost growth in coverage moves it by less than one percentage point.

**Figure 10:**
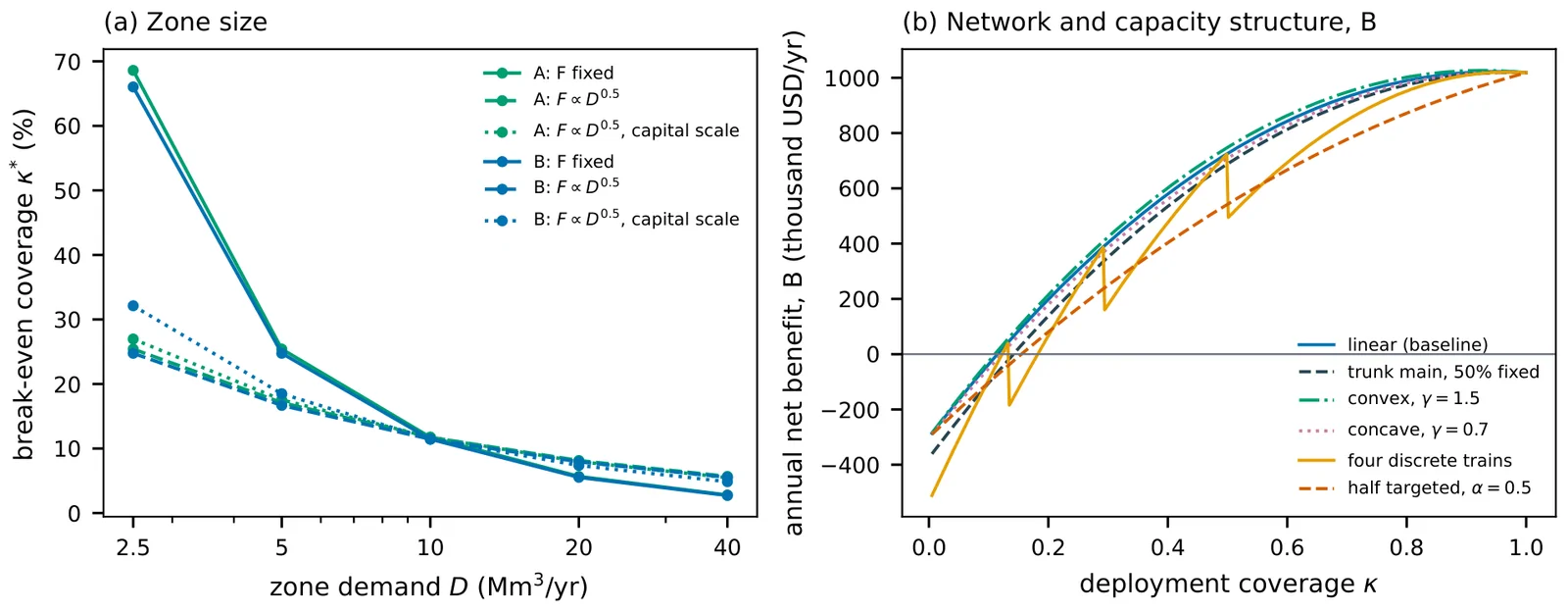
(**a**) Break-even coverage against zone demand *D* with the fixed cost held constant, scaled with $D^{0.5}$, and scaled with $D^{0.5}$ together with economies of scale in plant capital, v=1 USD/m^3^. (**b**) Net benefit of Configuration B against coverage under alternative network-cost, capacity, and connection-order structures, v=1 USD/m^3^, nominal parameter set.

Capacity and scale. Building the plant for full coverage at the outset, rather than adding capacity with demand, raises break-even coverage to 18.1% for Configuration A and 31.7% for Configuration B, because idle capacity is charged against a small initial demand and the capital share of Configuration B is larger. With two trains, the values are 14.0% and 18.2%, and with four trains they are within one percentage point of the baseline. Zone size matters more than any membrane input (Figure 10a). With the fixed cost held at 300,000 USD per year, break-even coverage is 68.6% and 66.0% in a zone of 2.5 Mm^3^ per year, 25.4% and 24.8% at 5 Mm^3^, and 2.8% and 2.7% at 40 Mm^3^. If the fixed cost scales with the square root of zone size, the dependence weakens to 25.4% and 24.8% at 2.5 Mm^3^ and 5.7% and 5.5% at 40 Mm^3^. The break-even coverages near 12% are therefore properties of a 10 Mm^3^ zone with the declared fixed cost, not of the membrane configurations.

Seasonal placement and compliance. The monthly balance of Appendix E gives a seasonal unplaced share of 10.5% for Configuration A and 11.6% for Configuration B without storage, so with a non-seasonal component of 4% the configuration-specific $\rho_{u}$ are 14.1% and 15.1%. Contrary to the expectation that a desalted product serves steadier demand, Configuration B is slightly more seasonal under the declared end uses, because its additional cooling-tower demand is itself seasonal and it serves all landscaping. With these values, break-even coverage is 12.2% and 12.6% and the crossover 1.03 USD/m^3^. One month of storage reduces the seasonal share to 2.2% and 3.2% and the crossover to 0.94 USD/m^3^, and two months remove it. Over an ensemble of seasonal amplitudes, pilgrimage timing, storage, and non-seasonal losses, the configuration-specific unplaced shares have medians of 10.6% and 11.6%, median break-even coverage is 11.6% and 12.0%, and Configuration B outperforms Configuration A in 43.4% of draws at 1 USD/m^3^, with a median crossover of 1.03 USD/m^3^. Out-of-class product is costly for both configurations. Configuration A breaks even until 52% of its annual product is diverted; Configuration B until 30%. Six excursions a year of three days each, with a response cost of 50,000 USD per event, cut the net benefit at 50% coverage by more than half under diversion, to 340,000 and 308,000 USD. Twelve week-long excursions make both configurations uneconomic under diversion, whereas retreating the out-of-class product instead restores a small positive net benefit for Configuration A only.

Concentrate management. Concentrate cost is the most consequential membrane-process assumption for Configuration B and the one that most distinguishes an inland from a coastal scheme. At 50% coverage, Configuration B produces 0.524 Mm^3^ of reverse-osmosis concentrate per year, about 1440 m^3^ per day, and every 0.1 USD/m^3^ of concentrate cost changes its net benefit by 52,000 USD per year. Eliminating the cost lowers the cost per cubic meter avoided to 0.63 USD/m^3^, break-even coverage to 9.9% and the crossover to 0.83 USD/m^3^. At 0.60 USD/m^3^, these become 0.76 USD/m^3^, 13.7% and 1.13 USD/m^3^, and at 1.0 USD/m^3^ they become 0.85 USD/m^3^, 18.4% and 1.33 USD/m^3^. Configuration B stops breaking even at 50% coverage above 1.69 USD per cubic meter of concentrate, and at 1 USD/m^3^ its advantage over Configuration A disappears above 0.34 USD/m^3^, just above the nominal value. A concentrate cost of 2.17 USD/m^3^ doubles the unit treatment cost of Configuration B, which corresponds to the doubling of the levelized cost that Giammar et al. estimate for adding zero liquid discharge [5]. At that level, Configuration B is uneconomic at 50% coverage and 1 USD/m^3^, and Configuration A would remain the better investment unless the water value exceeded about 1.9 USD/m^3^. Brine valorization acts in the opposite direction. With no net revenue, its best case is $c_{b}=0$, and any net revenue would improve Configuration B further at the same rate of 52,000 USD per year per 0.1 USD/m^3^. Across the paired draws, the share with negative net benefit at 50% coverage rises from 0.29% at the nominal 0.30 USD/m^3^ to 2.2% at 0.60, 13.7% at 1.0 and 68.9% at 2.0 USD/m^3^, and the share in which Configuration B outperforms Configuration A at 1 USD/m^3^ falls from 82.2% without concentrate cost to 16.3% at 0.60 and 2.8% at 1.0 USD/m^3^.

Fouling, element life and aging. Defining the reference operating state removes the risk of double counting, and its effect is small: with Φ=1, the no-double-counting bound, net benefit rises to 715,000 and 765,000 USD per year and break-even coverage falls to 11.6% and 11.0%. Progressive irreversible fouling matters more for Configuration B than for Configuration A. If the fouling penalty grows by 20% of its initial value per year of element age and the element life is halved, net benefit falls from 706,000 to 651,000 USD per year for Configuration A and from 726,000 to 473,000 USD for Configuration B, break-even coverage rises to 12.5% and 15.5%, and the share of paired draws with negative net benefit for Configuration B at 50% coverage rises from 0.29% to 6.3%, against 0.01% for Configuration A. The break-even element life stays between 1.23 and 1.45 years in every fouling combination for Configuration B, below the halved life of 3.0 years. Annualizing ultrafiltration and reverse-osmosis elements separately lowers the unit treatment cost of Configuration B from 0.559 to 0.548 USD/m^3^, because the ultrafiltration elements last longer, and gives break-even lives of 0.60 years for the reverse-osmosis elements and 0.74 years for the ultrafiltration elements of Configuration B, each with the other held at its nominal life. The aggregation therefore does not hide a binding replacement constraint, although it slightly understates the case for Configuration B: separate annualization raises the share of draws in which Configuration B outperforms Configuration A at 1 USD/m^3^ from 46.2% to 53.3%, while the total-effect index of each of the four separate element inputs stays at or below 0.015.

Salt-passage aging links element age to the quality class. Over 54 combinations of feed salinity, initial salt passage, salt-passage growth, and class limit, permeate reaches the class limit within the declared element life in the most adverse case: 3500 mg/L feed, 3% initial passage, 30% annual growth and a 200 mg/L limit, after 2.46 years. Replacing elements at that point raises the cost of Configuration B to 0.81 USD/m^3^ and lowers its net benefit to 455,000 USD per year. Running the elements to their declared life instead leaves the product out of class for 60% of the time, restricted to the uses of the non-desalted class, with a similar net benefit of 453,000 USD. In no combination does the effective element life fall below the break-even life of 1.23 years, and in none does net benefit become negative. The conclusion that element replacement is not the binding constraint survives aging, although aging can remove about 37% of the net benefit of Configuration B.

Feed salinity is a first-order driver of the configuration choice because it decides what the ultrafiltered product can serve. Below 450 mg/L, the ultrafiltered product carries no salinity restriction, so desalting adds no eligible demand and Configuration A dominates at every water value, with a net benefit of 1,503,000 USD per year against 744,000 USD. Between 450 and 2000 mg/L, the reference weights apply, and the higher osmotic pressure raises the specific energy of Configuration B only modestly, by 0.09 kWh/m^3^ at 1500 mg/L. Above 2000 mg/L, where irrigation with non-desalted water faces a severe restriction [14], the irrigation uses of Configuration A are halved, its break-even coverage rises to 19.3%, and the crossover falls to 0.80 USD/m^3^ at 2500 mg/L. At 3500 mg/L, it rises again to 0.84 USD/m^3^ because the specific energy of Configuration B increases to 1.19 kWh/m^3^ and its recovery falls to 0.745.

### 4.11. Scope of the Results

The model yields conditional break-even thresholds for the stated equations, parameter values, and cost boundaries. Four findings hold within that scope, each under stated conditions. First, under the declared eligibility weights and district mix, the recoverable volume is set by approved reuse demand rather than by membrane recovery in almost all draws, so recovery matters through concentrate cost rather than through yield. The reverse holds only where $R\phi <{\bar{e}}_{\varepsilon }$. Second, the choice between the two polymeric configurations turns on a crossover in the marginal value of water near 1 USD/m^3^ at the reference mix, 0.98 USD/m^3^ with full loss credit and 1.15 USD/m^3^ without. The crossover varies across the constructed districts from 0.85 to 1.39 USD/m^3^, or from 0.85 to 1.10 USD/m^3^ if the plant in the landscaped district draws on the whole effluent stream from the first phase, and moves with concentrate cost, capital and financing costs, feed salinity and the regulatory regime, and Configuration B outperforms Configuration A in 46.2% of paired draws at 1 USD/m^3^, so the choice near that value should rest on local measurement and quotations. Third, with independent inputs and the declared ranges, whether a scheme pays is governed by placement parameters, real losses, fixed cost, and zone size rather than by membrane performance. For Configuration B, however, a strong coupling of adverse-feed operating and cost parameters makes treatment-cost uncertainty as influential as placement (Section 4.6). Fourth, the break-even element life of 1.23 years for Configuration B is far below any reported service life and remains below the effective life under progressive fouling and salt-passage aging.

None of these findings is evidence about a specific plant. The cost ranges are planning ranges rather than local quotations, the eligibility weights encode an assumed regulatory position, and the fouling penalty is a declared scalar standing in for a process that depends on feedwater chemistry. Field use requires local measurement of effluent quantity and quality, approved end uses, network reach and procurement cost.

## 5. Deployment, Monitoring and Governance

A reclaimed-water scheme differs from a conventional treatment plant in that its product leaves the fence and enters a parallel distribution network serving many owners. The safeguards that follow are not optional additions to the economics: the fixed cost *F* of Equation (6) is largely the cost of running them, and it carries a total-effect Sobol index of 0.093 for Configuration B and 0.488 for Configuration A in the regime where that configuration can fail. Their failure also has a cost that the baseline omits: out-of-class product must be diverted or retreated, and Section 4.10 shows that six three-day excursions a year can halve the net benefit. Table 15 lists the monitoring and control functions assumed in *F*, with the authority level at which each action is taken.

The design follows risk-based water safety planning [46] and least-privilege access-control principles [47], and the allocation of authority follows established practice on levels of automation [48]. Analytics may recommend, a responsible human authority approves consequential actions, and no software component may override an independent quality interlock. Cross-connection prevention between the potable and reclaimed networks, and continuous verification of product quality against the class assumed in Table 2, are normative requirements rather than performance targets. Some monitoring can be automated within a pre-approved envelope, for example adjusting irrigation set-points on reclaimed supply, but only after a verified record at the approval-required level. This layer is a proposed specification. It has not been implemented, deployed, or evaluated, and no claim is made here about its operational performance.

## 6. Pilot Measurement Protocol: Madinah as an Illustrative Setting

### 6.1. Why Madinah Is Used as an Illustrative Setting

Madinah is a relevant setting for future measurement because its districts combine residents, accommodation, mosques, healthcare, commerce, and landscaping, which is precisely the heterogeneity that makes reuse eligibility uneven. The city had about 1.28 million residents in 2019 and receives more than eight million religious visitors per year [49]. Its arid climate, annual rainfall of about 40 mm and elevation of about 600–620 m [44] make desalination and conveyance energy relevant to the value of water [45], but no local marginal supply cost or financial avoided cost was available to place the city relative to the thresholds of Section 4.2 and Section 4.3. National policy addresses demand management, reuse and institutional coordination [50], while Saudi studies document both the quality constraints on reusing conventionally treated effluent [12,13] and the behavioral, institutional and acceptance constraints on reuse programs [26,27,51,52]. This paper analyzes no local utility telemetry and no local effluent characterization. The reference zone and the central-district entry in Table 5 are declared scenarios, not a Madinah case study, and nothing in this paper is a prediction for Madinah. The protocol below specifies what a pilot there, or in any comparable city, would need to measure.

### 6.2. What a Pilot Must Measure

Table 16 specifies how a pilot would measure each parameter, ordered by the total-effect Sobol index of Configuration B at 50% coverage under independent inputs. For the question of whether a scheme pays, the measurements that matter most under that ordering are of the effluent stream, the approved demand, and the fixed cost of compliance, not of membrane performance. Two qualifications follow from Section 4.2 and Section 4.6. If adverse feed conditions couple membrane operating and cost parameters, treatment-cost measurements gain priority for Configuration B. Near the crossover, where the configuration choice is at stake, the value of information is concentrated in the unit treatment costs of the two trains. Membrane recovery, specific energy, element life, salt rejection and fouling behavior therefore require measurement on local effluent, and a firm quotation for both trains should precede the configuration decision. Feed salinity, the permitted concentrate route, and the frequency of quality excursions, which the baseline does not treat as uncertain inputs, are added to the protocol because Section 4.10 shows that each can change the configuration choice.

### 6.3. Pilot Design and Statistical Power

The demand-side effect of a reuse scheme is large and directly metered, which distinguishes it from behavioral conservation programs. At the nominal parameter set, serving half of the eligible connections in a cluster reduces that cluster’s potable consumption by 20.4% under Configuration B and 11.5% under Configuration A. Both effects are model outputs, not measurements, and a partial uptake of reclaimed water would produce a smaller effect. We compute the minimum detectable effect (MDE) of a stepped-wedge design with *I* clusters over *T* periods from the variance of the treatment effect [53], as given in Equation (17):

(17) $$\mathrm{Var}\left(\hat{\delta }\right)=\frac{I\sigma^{2}\left(\sigma^{2}+T\tau^{2}\right)}{\left(IU-W\right)\sigma^{2}+\left(U^{2}+ITU-TW-IV\right)\tau^{2}},\;\mathrm{MDE}=\left(z_{0.975}+z_{0.8}\right)\sqrt{\mathrm{Var}(\hat{\delta })},$$

where $X_{ij}\in \left\{0,1\right\}$ indicates treatment of cluster *i* in period *j*, $U=\sum_{ij}X_{ij}$, $W=\sum_{j}{\left(\sum_{i}X_{ij}\right)}^{2}$, $V=\sum_{i}{\left(\sum_{j}X_{ij}\right)}^{2}$, σ is the residual standard deviation of a cluster-period mean of normalized log consumption and τ the between-cluster standard deviation [54]. With declared planning values σ=5% and τ=10%, a four-step stepped wedge over eight clusters detects an effect of 7.96%, and twelve clusters detect 6.50%. Under these values, four clusters would suffice for the nominal 20.4% substitution effect, for the 11.5% effect of Configuration A, and for the 16.7% reduction that would be needed for the avoided volume of Configuration B alone to offset its annualized cost at 1 USD/m^3^.

These planning values are optimistic, because σ, τ and the substitution effect are all assumed rather than measured, and seasonal demand, unequal cluster sizes, visitor loads and temporal autocorrelation can each inflate the variance of the estimated effect. Appendix F therefore computes the minimum detectable effect from the generalized least-squares variance of the treatment effect for a model with period fixed effects and a cluster-period random effect whose correlation decays as $r^{\left|t-s\right|}$ between periods [55]. The model also allows unequal cluster sizes with a coefficient of variation up to 1 and an additional cluster-period variance for uneven visitor loads. With r=1, equal sizes and no additional variance, it reproduces Equation (17) exactly. Over σ from 2.5% to 15%, τ from 5% to 20%, and these structures, the number of clusters needed rises from 4 to 28 for the nominal effect of Configuration B and from 4 to 80 for that of Configuration A. For a 10% effect, it exceeds 100 in the least favorable structures (Table A6). The residual variation σ dominates: at σ=15%, an eight-cluster design detects only 22% and twelve clusters are needed for the nominal effect of Configuration B. Decaying temporal correlation and visitor-load variance come next, while the between-cluster variance and unequal cluster sizes matter little in a stepped-wedge design, which compares each cluster with itself. The earlier statement that four clusters suffice therefore holds only if residual variation is as low as the planning value and the realized substitution is close to its nominal size. A pilot should be sized from a variance pre-study in Stage 0 that measures σ, τ and the temporal correlation of cluster-period consumption.

Statistical power is not the only constraint. Product-water quality must be demonstrated against the assumed class before any supply is approved, which is a regulatory rather than a statistical question. The unplaced share $\rho_{u}$ requires a full irrigation cycle to observe, because it is driven by seasonal mismatch, and pilgrimage peaks move through the seasons from year to year. Fouling behavior and element life require a pilot run long enough to observe at least one full cleaning cycle and a stable flux-decline trend on local effluent, which cannot be compressed. A pilot should therefore be designed around these observation windows as well as around cluster counts.

### 6.4. Adoption Roadmap

Table 17 summarizes the evidence gates. Stage 0 can reject further evaluation when measured eligible demand, effluent availability, fixed cost, or network cost make break-even implausible, using Equations (11) and (13) directly. Passing Stage 0 does not authorize construction, because the unit treatment cost that must be compared with $B^{*}$ is not known until a pilot train has run on local effluent.

## 7. Discussion

### 7.1. Treatment Performance Versus Economic Viability

The clearest implication of Section 4.6 is that, under the declared ranges and with independent inputs, membrane performance is not what decides whether a reuse scheme pays. Element capital, element life, recovery, specific energy, and the fouling penalty together carry about 0.10 of the total-effect variance for Configuration B and less than 0.02 for Configuration A. What decides the case is how much demand may legally and technically accept the product, how concentrated that demand is among connections, how much of the product can actually be placed through the year, how large the standing compliance cost is, and how large the zone is. Two qualifications keep this from becoming a general rule. First, it is conditional on how the membrane parameters co-vary: if an adverse feed raises energy, cleaning and capital together while lowering recovery and element life, the treatment-cost group of Configuration B becomes as influential as the placement group (Table 9). Second, treatment costs decide which configuration to choose even where they do not decide whether to build: near the crossover, most of the value of information lies in the unit treatment costs of the two trains (Table 7). This does not mean membrane research is irrelevant to deployment. It means that the marginal decision value of further improvement in these particular properties is small relative to the value of resolving the placement question, given the ranges that current materials already achieve. Improvements that widen the set of approved end uses, for instance, better rejection of the small neutral organics that currently require a downstream barrier [8,9], would act on $\bar{e}$ and would therefore matter a great deal more than an incremental gain in flux.

The break-even element life of 1.23 years for Configuration B makes the same point quantitatively. Reverse-osmosis element lifespans are often 5 to 10 years [16], several times that value, so a scheme that fails does not fail because its membranes wore out, and this remains true under progressive fouling and salt-passage aging (Section 4.10). It fails because too little of its product could be placed, because the standing cost of certifying it was too high, or because the zone was too small for that standing cost.

### 7.2. Membrane Recovery and Water Yield

The finding that approved demand rather than membrane recovery binds the volume in 98.8% of draws for Configuration B has a direct design consequence. Pushing recovery higher in an urban reuse scheme of this type buys concentrate reduction, not extra product. The concentrate term $c_{b}\left(1/R_{\mathrm{RO}}-1\right)$ contributes 0.068 USD/m^3^ at the nominal reverse-osmosis recovery of 81.5%, and concentrate cost is the most consequential membrane-process assumption in Table 14, so recovery remains worth optimizing inland, but for a different reason than the usual one. In a coastal scheme with a low-cost concentrate sink, the case for high recovery in this configuration is correspondingly weaker. The exception is the landscaped district scenario, where eligible demand exceeds the available product stream and recovery does buy volume. There, whether the first phase can draw on the whole effluent stream also decides whether the crossover lies above or below 1 USD/m^3^ (Section 4.8). The general rule, stated precisely in Section 3.4, is that recovery matters for yield only where $R\phi <{\bar{e}}_{\varepsilon }$.

### 7.3. Fouling, Cleaning and Replacement

Fouling enters the baseline model through a penalty multiplier on energy and cleaning, defined relative to the reference operating state of the cited trains, and through element life. Both are modest contributors under the declared ranges, with total-effect indices of 0.004 and 0.030 for Configuration B. Treating fouling as progressive changes this only in degree: a penalty that grows by 20% of its initial value per year, combined with a halved element life, removes about a third of the net benefit of Configuration B and raises its break-even coverage to 15.5%, but leaves its break-even element life far below the effective life. The stronger effect comes from coupling. If one adverse feed raises fouling, energy and capital together while lowering recovery and element life, the share of variance carried by the treatment-cost group of Configuration B rises several-fold (Table 9). Wastewater characteristics and operating conditions shape this coupling: scaling by sparingly soluble salts depends on feed composition and recovery [36] and can be mitigated by operating strategy [18], while organic and biological fouling depend on pretreatment [15,22]. The practical reading is that pretreatment adequacy and feed chemistry are gates rather than optimization variables. Configuration B uses the ultrafiltration stage as pretreatment precisely to keep the fouling penalty inside the declared range, and a pilot that fails to demonstrate a stable flux-decline trend on local effluent invalidates the cost model rather than merely perturbing it.

### 7.4. Process Integration and Practical Implementation

Three integration questions follow from the model. First, the reclaimed network is the binding physical asset, not the plant. Coverage is defined over eligible connections, and $\kappa^{{}^{\circ}}$ near 95% indicates that the network should be planned to near-complete coverage of eligible demand even though the first phase can be much smaller. Second, plant capacity and network coverage advance together in the baseline, which is the correct assumption for a modular membrane plant. A plant built for full coverage at the outset raises break-even coverage to 18.1% for Configuration A and 31.7% for Configuration B, whereas staging the plant in four trains keeps it within one percentage point of the baseline. Staged construction therefore matters most for the capital-intensive Configuration B. Third, seasonal mismatch between a continuous effluent stream and seasonal irrigation and cooling loads is carried by $\rho_{u}$, whose total-effect index for Configuration B, 0.168, is almost equal to the second largest. In the monthly balance of Appendix E, one month of storage removes most of the seasonal unplaced share and lowers break-even coverage from 12.2% to 10.7% for Configuration A and from 12.6% to 10.1% for Configuration B. Storage, aquifer recharge, or a seasonal industrial offtake are therefore among the highest-value integration measures available, more so than any improvement in the membranes themselves, provided that their own cost is below the value of the volume they place.

### 7.5. Reuse and Other Programs

This paper does not rank membrane reuse against pressure management or a tariff program on a unit-cost axis. The programs deliver different functional outputs: pressure management avoids losses in the existing potable network and is bounded by the losses that exist, a tariff program reduces demand, and a reuse scheme creates a second source of supply of a defined quality that is bounded by approved demand. Their cost boundaries are also not matched. The reuse figures include distribution to the end user, whereas the pressure-management cost data available to this study exclude network access, valve procurement, calibration, and institutional costs. A genuinely matched incremental cost-effectiveness comparison would need those costs for the same zone, together with the institutional, distributional and political costs of a tariff program [28], and is left to studies with such data. What the analysis does show is that the programs differ in scale, and that prior expansion of loss control and demand management erodes the reuse case only modestly (Section 4.9). Double counting of shared benefits is avoided by construction in Equation (9).

### 7.6. Polymer Membrane Properties and the Decision Variables

The decision variables of this paper respond to polymer membrane properties through a small number of model parameters, and Table 18 makes that link explicit with the material evidence cited in Section 2. Three properties act on the costs. The oxidant and alkaline tolerance of the ultrafiltration polymer sets how cleaning ages the fibers: hypochlorite aging of polyethersulfone raised permeability several-fold and cut retention [19], and alkaline cleaning of PVDF gave modeled lifetimes of 1.8 to 7 years [20]. In the model, this acts through the ultrafiltration element life and, through loss of integrity, through the risk of out-of-class product. The fouling resistance of the membrane surface acts through the fouling penalty and its growth over the element life. The scaling propensity of the reverse-osmosis stage at high recovery acts through the recovery and therefore the concentrate volume. A fourth property acts on the volume rather than the cost: the salt rejection of the polyamide layer and its stability under oxidant exposure [16] decide whether the product stays in the desalted quality class, and therefore how much demand it may serve.

The computed effects separate these properties by the decision they affect. Properties that change element life or the fouling penalty mainly move the configuration crossover and, for Configuration B, the break-even coverage. An ultrafiltration life of 1.8 years lowers the crossover from 0.98 to 0.86 USD/m^3^, because it raises the cost of the cheaper configuration, and a reverse-osmosis life of 3 or 10 years moves it to 1.16 or 0.91 USD/m^3^. They do not change whether a well-placed scheme pays. Rejection and its stability act on the eligible demand, which is the larger lever: an ultrafiltered product below 450 mg/L of dissolved solids makes the reverse-osmosis stage unnecessary, and permeate that leaves its class removes up to 37% of the net benefit of Configuration B. For a materials program aimed at reuse deployment, the most decision-relevant targets are therefore stable salt and micropollutant rejection over the element life and oxidant-tolerant ultrafiltration polymers, rather than incremental gains in permeability.

### 7.7. Conditions to Check When Transferring the Screen

The screen transfers to other urban reuse systems only if the inputs that drive it are re-established for the new system. The analysis identifies a short list, ordered by the decision each one affects. For whether to build, the conditions are the reuse-eligible demand and its regulatory basis; the zone size relative to the fixed compliance and monitoring cost; the concentration of eligible demand among candidate connections and whether the utility can connect in that order; the share of real losses that reduced throughput would actually avoid; and the seasonal profile of eligible demand together with the storage available. For which configuration to build, they are the feed salinity relative to the salinity limits of the intended uses, which can make desalting unnecessary or indispensable; the permitted concentrate route and its cost, the most consequential membrane-process term for an inland scheme; the capital cost and financing terms of the reverse-osmosis train; the electricity price; and the expected element lives of both trains on the local feed. A coastal scheme with a low-cost concentrate sink, a low-salinity effluent, a small zone, or a utility facing only its financial avoided cost will therefore reach different thresholds with identical membranes.

### 7.8. Limitations

No new membrane experiments, characterization, or pilot measurements were performed for this study. All membrane properties are literature-derived or declared, and no local Madinah effluent characterization, membrane test, or procurement quotation was available. The reported values are model outputs under declared assumptions and are not evidence about any plant.

The declared distributions are scenario choices informed to varying degrees by the literature in Appendix C, not calibrated beliefs. The baseline ensemble assumes independent inputs. The dependence analysis of Section 4.6 bounds the consequences for one adverse-feed direction of coupling, but it cannot establish the actual dependence structure, which requires joint measurements of recovery, fouling, element life, energy and cost on the same feed. Sensitivity indices reflect both the model and the declared input ranges [35]. Cost ranges, in particular *F*, $c_{\mathrm{cap}}$ and $c_{b}$, are generic literature calibrations and planning ranges that do not represent Saudi procurement. No local quotation, regional cost study, or local concentrate permit was available to this study.

The fouling penalty remains a reduced representation of a process that depends on feedwater chemistry, flux, temperature, and cleaning protocol. Progressive fouling and salt-passage aging are represented by declared scenarios with simple time profiles rather than by a mechanistic fouling model. The feed-salinity scenarios use a single-stage osmotic-pressure bound and declared adjustments of recovery and eligibility rather than a process model of the reverse-osmosis train. Treated-water quality is a fixed class in the baseline, and the compliance-failure scenario represents excursions only through their frequency, duration, and response cost.

The eligibility weights of Table 2 are declared judgments about what a regulator would approve. No regulatory determination, end-use survey, or expert elicitation was available, and the common multiplier, the sector-specific multipliers, and the discrete regulatory scenarios bound their effect but do not replace such a determination. The eligibility scale ε carries the largest total-effect index for Configuration A. The reach function is a one-parameter concave form without empirical calibration, and network topology, trunk sizing, pumping, storage, and the clustering of large users are represented only through the alternative network-cost structures of Section 4.10. The alternative power form changes nominal net benefit by about a fifth.

The accounting is annual. The monthly balance of Appendix E uses declared seasonal profiles rather than local demand data, and storage or aquifer recharge is represented through its effect on the unplaced share, not through its cost. Reclaimed-network pumping is included as a range rather than from a network design. The directional effect of each remaining simplification, and where it is bounded, is given in Table 19. Finally, the monitoring and governance layer of Section 5 is a specification. It has not been implemented or evaluated, and its cost is represented only through *F*.

### 7.9. Implications for Research

The conditional sensitivity ranking suggests where research effort is most decision-relevant for deployment. The placement parameters $\rho_{u}$, $\bar{e}$ and $\theta_{E}$ have the weakest evidence base of all inputs used here and, together with the real-loss share, the largest influence on the result, yet none is a membrane property. The dependence analysis adds a second need: joint measurements of recovery, fouling, element life, energy and cost on the same effluent, without which the coupling that matters most for Configuration B cannot be quantified. On the materials side, the most decision-relevant open problems are those that widen the approved-use set or keep the product in its class: rejection of small neutral organics and of species such as boron that currently require a separate barrier [8,9], salt rejection that remains stable under oxidant exposure over the element life [16], ultrafiltration polymers that tolerate cleaning chemicals [19,20], and fouling-resistant chemistries that hold the fouling penalty inside a declarable range on real secondary effluent [4,15]. Concentrate valorization matters for the same economic reason that recovery does inland [6]. Finally, the release of polymer fragments from ultrafiltration systems under aggressive cleaning [11] is a quality question that the present quality-class framing cannot represent and that a reuse scheme supplying irrigation water should address directly.

## 8. Conclusions

A utility in an arid city deciding whether to build a polymeric membrane reuse scheme faces two coupled questions: which membrane class, and how far to build the reclaimed network. This paper derives conditional break-even equations from a transparent volume and cost model of two polymeric configurations, evaluates them across declared scenario distributions with coverage treated as the decision, and proposes a staged measurement process.

For an illustrative reference zone of $10\;{\mathrm{Mm}}^{3}$ per year at a marginal water value of 1 USD/m^3^, a polymeric ultrafiltration scheme serving half of the eligible connections avoids 11.5 percentage points of potable system input at 0.48 USD per cubic meter avoided and breaks even above 11.7% coverage. Adding polyamide reverse osmosis raises the avoided input to 20.4 points at 0.70 USD/m^3^, with break-even above 11.5% coverage. Without credit for avoided real losses, these costs rise to 0.56 and 0.82 USD/m^3^ and the break-even coverages to 14.9% and 18.2%. Reverse osmosis is the better investment above a crossover water value of 0.98 USD/m^3^ at the reference mix, or 1.15 USD/m^3^ without loss credit. The crossover ranges from 0.85 to 1.39 USD/m^3^ across the constructed district scenarios, or from 0.85 to 1.10 USD/m^3^ if the landscaped district’s plant can draw on the whole effluent stream from the first phase, and Configuration B outperforms Configuration A in 46.2% of paired draws at 1 USD/m^3^. Untargeted connection order roughly doubles the break-even coverage, and in a zone a quarter of the size with the same fixed cost, it exceeds 60%.

Four conclusions follow for practice, each conditional on the stated assumptions. First, with the declared eligibility weights and district mix, the recoverable volume is set by approved reuse demand, not by membrane recovery, in 98.8% of draws for Configuration B, so recovery should be optimized for concentrate reduction rather than for yield. The reverse holds only where $R\phi <{\bar{e}}_{\varepsilon }$. Second, the break-even unit treatment cost, 0.76 and 0.87 USD/m^3^ for the two configurations at 50% coverage, is the quantity a utility should compare with a supplier quotation, and the break-even element life of 1.23 years shows that replacement is not the binding constraint, even under progressive fouling and salt-passage aging. Third, whether to build is governed by real losses, the unplaced-product share, the concentration and extent of reuse-eligible demand, the standing compliance cost, and the zone size. With independent inputs, all membrane-specific inputs together contribute about 0.10 of the total-effect variance for Configuration B, but if adverse feed conditions couple operating and cost parameters, treatment-cost uncertainty becomes as influential as placement for that configuration. Fourth, which configuration to build is governed by the cost premium of desalting and by what desalting adds to the eligible demand. Concentrate management, capital and financing costs, electricity price, element life, and the regulatory regime each move the crossover by 0.15 to 0.31 USD/m^3^ over their declared ranges. Feed salinity lowers it to 0.80–0.84 USD/m^3^ above 2000 mg/L and removes any advantage of reverse osmosis below 450 mg/L. Near the crossover, a firm quotation for both trains is worth more than refined placement data.

What this paper does not establish is equally clear. It performed no membrane experiments and used no local effluent characterization, procurement data, regulatory determination of eligible uses, or marginal supply cost, and its distributions are declared scenario choices rather than empirical probabilities. Madinah is an illustrative decision context, not a case study. The next step is not construction. It is a Stage 0 characterization in a candidate district of effluent quantity, quality and salinity, sector end uses, the permitted concentrate route and compliance cost, with a variance pre-study for the pilot design. A pilot membrane train should then run on local effluent long enough to measure a unit treatment cost that can be compared with $B^{*}$.

## Figures and Tables

**Table 1:** The two polymeric membrane configurations, their separation mechanisms, the water-quality objectives they are intended to support, and their principal residual limitations. Rejection characteristics are literature-derived and are not measurements made in this study.

Attribute	Configuration A: Ultrafiltration	Configuration B: Ultrafiltration with Reverse Osmosis
Membrane material	Hollow-fiber polyvinylidene fluoride or polyethersulfone, nominal pore size 0.01–0.1 μm [4,7]	The same ultrafiltration stage as pretreatment, followed by spiral-wound aromatic polyamide thin-film composite on a polysulfone support [1,2]
Separation mechanism	Size exclusion and cake filtration	Size exclusion at the first stage; solution-diffusion with charge and hydrophobicity effects at the second [8]
Constituents controlled	Suspended solids, turbidity, colloids, protozoa, most bacteria, microplastic particles [7,11]	The above, plus dissolved salts, a large fraction of dissolved organic micropollutants and perfluoroalkyl acids [8,10]
Constituents not controlled	Dissolved salts, boron, dissolved organic micropollutants, most dissolved nutrients	Small neutral organics such as N-nitrosodimethylamine; boron at neutral pH [9]
Resulting quality class	Non-desalted reclaimed water for restricted irrigation and toilet flushing	Desalted reclaimed water suitable additionally for salinity-sensitive planting and cooling-tower makeup
Principal operational burden	Fouling and backwash management; polymer fragment release under aggressive cleaning [11]	Organic, colloidal and biological fouling of the polyamide layer; cleaning; element replacement; concentrate management [6,15]

**Table 2:** Sector reuse-eligibility weights $e_{i}$ by product-water quality class, with the end uses assumed and the constraints that limit eligibility. Weights are author-declared scenario assumptions, not measured shares. Their decomposition into end uses is given in Table A2.

Sector	Assumed Reclaimed End Uses	Limiting Constraint	$e_{i}$ (A)	$e_{i}$ (B)
Residential	Communal landscape irrigation; toilet flushing where dual plumbing exists	Retrofit of internal plumbing is rarely feasible	0.10	0.10
Hospitality	Grounds irrigation, cooling-tower makeup, external washdown	Cooling towers require low salinity to run at useful cycles of concentration	0.10	0.25
Mosques and ablution	Grounds irrigation, cleaning, toilet flushing; ablution excluded	Ritual function requires potable supply	0.25	0.30
Healthcare	Cooling-tower makeup only	Clinical and sanitary uses require potable supply	0.00	0.05
Commerce and institutions	Cooling towers, toilet flushing, washdown	Cooling-tower makeup needs desalted water	0.10	0.35
Landscaping	Amenity and street planting	Salinity-sensitive species need desalted water; restricted-access irrigation does not	0.55	1.00

**Table 3:** Declared model inputs. TN(μ,s) is a normal distribution with mean μ and scale *s* truncated to the stated support, and U is uniform. Nominal values are distribution means. Classification is: L, literature-derived; D, derived from other parameters or from a literature range; S, author-declared scenario assumption; P, declared planning range. The evidence and its translation are given in Appendix C. Inputs in the first block take one common value for both configurations in every Monte Carlo realization. The concentrate cost $c_{b}$ is per cubic meter of reverse-osmosis concentrate. UF, ultrafiltration; RO, reverse osmosis.

Symbol	Meaning	Distribution and Support	Nominal	Units	Class
Shared between configurations					
$\lambda_{p}$	Real losses, share of potable system input	TN(0.15, 0.05), [0.02, 0.28]	0.15	–	L
ϕ	Effluent return ratio	TN(0.60, 0.08), [0.40, 0.80]	0.60	–	S
ε	Scale on reuse-eligibility weights	TN(1.00, 0.15), [0.70, 1.30]	1.00	–	S
$\rho_{u}$	Unplaced share of product water	TN(0.12, 0.05), [0.02, 0.22]	0.12	–	S
$\theta_{E}$	Eligible-demand concentration	TN(2.0, 0.5), [1.0, 3.0]	2.0	–	S
Φ	Fouling penalty on energy and cleaning	TN(1.20, 0.10), [1.00, 1.50]	1.20	–	S
$c_{\mathrm{net}}$	Reclaimed network cost per connection	U[60, 180]	120	USD/yr	P
*F*	Fixed annual cost	U[150, 450]	300	kUSD/yr	P
$R_{\mathrm{UF}}$	UF-stage recovery (overall for A)	TN(0.92, 0.02), [0.86, 0.97]	0.92	–	L
Configuration A: ultrafiltration					
SEC	Specific electrical energy	TN(0.20, 0.05), [0.10, 0.35]	0.20	kWh/m^3^	L
$T_{m}$	Membrane element service life	TN(8.0, 1.5), [5.0, 12.0]	8.0	yr	L
$c_{\mathrm{cap}}$	Plant capital, excluding elements	U[0.70, 1.40]	1.05	USD per m^3^/yr	L
$c_{\mathrm{mem}}$	Membrane element capital	U[0.12, 0.26]	0.19	USD per m^3^/yr	L
Configuration B: ultrafiltration with reverse osmosis					
$R_{\mathrm{RO}}$	RO-stage recovery	TN(0.82, 0.05), [0.70, 0.90]	0.815	–	D
SEC	Specific electrical energy	TN(0.85, 0.18), [0.45, 1.25]	0.85	kWh/m^3^	L
$T_{m}$	Membrane element service life	TN(6.0, 1.5), [3.0, 10.0]	6.0	yr	L
$c_{\mathrm{cap}}$	Plant capital, excluding elements	U[1.60, 3.20]	2.40	USD per m^3^/yr	L
$c_{\mathrm{mem}}$	Membrane element capital	U[0.25, 0.60]	0.425	USD per m^3^/yr	L
Sequencing programs (Frame 2 only)					
*r*	Demand response to tariff or fixture program	Beta(3, 7) on [0, 0.20]	0.060	–	L
$\beta_{r}$	Loss reduction by pressure-management expansion	TN(0.22, 0.05), [0.07, 0.37]	0.22	–	L
Declared deterministic constants					
*i*	Real discount rate	–	0.06	yr^−1^	P
$T_{p}$	Plant economic life	–	25	yr	P
*u*	Utilization of installed capacity	–	0.90	–	P
$\pi_{E}$	Electricity price	–	0.06	USD/kWh	P
$c_{\mathrm{cip}}$	Cleaning chemicals (A; B)	–	0.020; 0.030	USD/m^3^	P
$c_{\mathrm{om}}$	Other operation and maintenance (A; B)	–	0.060; 0.090	USD/m^3^	P
$c_{b}$	Concentrate management (A; B)	–	0.00; 0.30	USD/m^3^	P

**Table 4:** Decomposition of the nominal unit treatment cost *B* (Equation (7)) and of the annualized cost $Z_{M}$ at κ=0.5. Every component is a model output from generic literature calibrations and planning assumptions, not a Saudi procurement figure. Components may not add to the totals because of rounding.

Component	Configuration A	Configuration B
Unit treatment cost, USD per m^3^ of product		
Plant capital, $\mathrm{CRF}\left(i,T_{p}\right)c_{\mathrm{cap}}/u$	0.091	0.209
Membrane elements, $\mathrm{CRF}\left(i,T_{m}\right)c_{\mathrm{mem}}/u$	0.034	0.095
Energy, $\pi_{E}\Phi \;\mathrm{SEC}$	0.015	0.061
Cleaning chemicals, $\Phi c_{\mathrm{cip}}$	0.024	0.036
Other operation and maintenance, $c_{\mathrm{om}}$	0.060	0.090
Concentrate, $c_{b}\left(1/R_{\mathrm{RO}}-1\right)$	0.000	0.068
Total *B*	0.224	0.559
Annualized cost at κ=0.5, kUSD/yr		
Treatment, BW	292.9	1294.2
Fixed compliance, monitoring and staff, *F*	300.0	300.0
Reclaimed network, $c_{\mathrm{net}}M_{e}\kappa$	55.7	74.9
Total $Z_{M}$	648.6	1669.1

**Table 5:** Constructed district scenarios. Sector entries are shares of baseline potable demand *D*. The table also gives the declared real-loss level $\lambda_{p}$ and the resulting reuse-eligible demand share $\bar{e}$ at ε=1 for each configuration. Several attributes change together, so this is not a one-factor sector-mix design.

Mix	Resid.	Hosp.	Mosques	Health	Comm.	Landsc.	$\lambda_{p}$	$\bar{e}$ (A)	$\bar{e}$ (B)
Reference mixed district	0.40	0.20	0.08	0.05	0.12	0.15	0.15	0.175	0.309
Residential periphery	0.65	0.05	0.05	0.05	0.10	0.10	0.18	0.148	0.230
Central district, pilgrimage peak	0.25	0.35	0.15	0.04	0.13	0.08	0.12	0.155	0.285
Healthcare and institutional campus	0.10	0.05	0.05	0.40	0.30	0.10	0.10	0.113	0.263
Landscaped district	0.30	0.05	0.05	0.02	0.08	0.50	0.20	0.331	0.587

**Table 6:** Break-even conditions at the nominal parameter set, reference mix, κ=0.5 unless stated. Thresholds are defined by NB=0 (Equations (11)–(13)). The entry n.a. (not attained) means that no coverage in [0,1] is cost-effective, and a dash means that no element life makes the scheme economic. The volumes, the annualized cost $Z_{M}$ and the three unit costs do not depend on *v*. The lower block repeats the main quantities without loss credit ($\eta_{L}=0$).

Quantity	Configuration A (UF)			Configuration B (UF + RO)		
	v=0.5	v=1	v=2	v=0.5	v=1	v=2
Product water *W* (Mm^3^/yr)	1.309	1.309	1.309	2.314	2.314	2.314
Avoided potable input $V_{M}$ (Mm^3^/yr)	1.355	1.355	1.355	2.395	2.395	2.395
Saving $\Delta_{M}$ (pp of system input)	11.52	11.52	11.52	20.36	20.36	20.36
Annualized cost $Z_{M}$ (kUSD/yr)	649	649	649	1669	1669	1669
$u_{W}$, per m^3^ of product (USD/m^3^)	0.50	0.50	0.50	0.72	0.72	0.72
$u_{P}$, per m^3^ placed (USD/m^3^)	0.56	0.56	0.56	0.82	0.82	0.82
$u_{M}$, per m^3^ of potable input avoided (USD/m^3^)	0.48	0.48	0.48	0.70	0.70	0.70
Net benefit (kUSD/yr)	29	706	2061	−471	726	3122
Unit treatment cost *B* (USD/m^3^)	0.224	0.224	0.224	0.559	0.559	0.559
Break-even $B^{*}$ (USD/m^3^)	0.246	0.764	1.799	0.356	0.873	1.909
Break-even element life $T_{m}^{*}$ (yr)	4.41	0.38	0.14	–	1.23	0.34
Break-even coverage $\kappa^{*}$, targeted	43.4%	11.7%	4.9%	n.a.	11.5%	3.3%
Break-even coverage $\kappa^{*}$, untargeted	74.7%	23.0%	9.6%	n.a.	22.8%	6.6%
Optimal coverage $\kappa^{{}^{\circ}}$, targeted	89.1%	96.1%	98.3%	–	94.9%	98.4%
Without loss credit, $\eta_{L}=0$						
Avoided potable input $V_{M}$ (Mm^3^/yr)	1.152	1.152	1.152	2.036	2.036	2.036
$u_{M}$ (USD/m^3^)	0.56	0.56	0.56	0.82	0.82	0.82
Net benefit (kUSD/yr)	−73	503	1655	−651	367	2403
Break-even coverage $\kappa^{*}$, targeted	n.a.	14.9%	5.9%	n.a.	18.2%	4.2%

**Table 7:** Decision metrics for the configuration choice over the 200,000 paired draws at κ=0.5. Regret is the expected shortfall of a choice relative to the best of no scheme, Configuration A, and Configuration B in each draw. EVPI is the expected value of perfect information and EVPPI the expected value of partial perfect information for the stated group of inputs, estimated by nonparametric regression [41]. Membrane-specific unit costs comprise elements, energy, cleaning, and concentrate. Values are properties of the declared ensemble, not probabilities for a city.

Quantity	v=0.75	v=1.0	v=1.25	v=1.5
Share of draws with ${\mathrm{NB}}_{B}>{\mathrm{NB}}_{A}$	3.0%	46.2%	91.3%	99.5%
Share with ${\mathrm{NB}}_{A}>0$	98.9%	>99.9%	>99.9%	>99.9%
Share with ${\mathrm{NB}}_{B}>0$	66.5%	99.7%	>99.9%	>99.9%
Expected NB, A (kUSD/yr)	358	693	1028	1362
Expected NB, B (kUSD/yr)	99	674	1250	1825
Choice maximizing expected NB	A	A	B	B
Expected regret of A (kUSD/yr)	1.8	52.1	229.2	463.4
Expected regret of B (kUSD/yr)	261.5	70.9	7.0	0.3
EVPI (kUSD/yr)	1.8	52.1	7.0	0.3
EVPPI, unit treatment costs $B_{A}$, $B_{B}$	0.4	43.0	2.6	0.0
EVPPI, membrane-specific unit costs	0.0	26.7	0.6	0.0
EVPPI, plant capital	0.0	30.5	0.0	0.0
EVPPI, placement ε, $\rho_{u}$, $\theta_{E}$	0.0	15.5	0.0	0.0
EVPPI, real losses $\lambda_{p}$	0.0	14.3	0.0	0.0
EVPPI, fixed and network cost	0.0	0.0	0.0	0.0

**Table 8:** Sobol indices of annual net benefit within the declared independent scenario design, targeted connection order and $N=2^{15}$ base samples, with 524,288 evaluations per case for Configuration B and 491,520 for Configuration A. Brackets are 2.5th to 97.5th percentile row-bootstrap stability ranges from 1000 resamples, not inferential confidence intervals. Inputs are ordered by the total-effect index of Configuration B at κ=0.5. The final two columns are Configuration A, which has no reverse-osmosis stage.

Input	Configuration B, v=1, κ=0.5				B, $S_{\mathrm{Tj}}$		A, $S_{\mathrm{Tj}}$, κ=0.5	
	$S_{j}$	Bootstrap Range	$S_{\mathrm{Tj}}$	Bootstrap Range	κ=0.5	κ=0.7	v=1	v=0.5
$\lambda_{p}$	0.214	[0.196, 0.234]	0.220	[0.216, 0.224]	0.174	0.254	0.117	0.098
$\theta_{E}$	0.162	[0.146, 0.178]	0.170	[0.167, 0.174]	0.269	0.065	0.260	0.115
$\rho_{u}$	0.164	[0.148, 0.181]	0.168	[0.165, 0.171]	0.133	0.195	0.090	0.075
ε	0.146	[0.131, 0.161]	0.155	[0.152, 0.158]	0.123	0.180	0.380	0.169
$c_{\mathrm{cap}}$	0.098	[0.086, 0.110]	0.100	[0.099, 0.102]	0.079	0.116	0.010	0.034
*F*	0.093	[0.082, 0.104]	0.093	[0.091, 0.094]	0.156	0.074	0.146	0.488
$c_{\mathrm{mem}}$	0.034	[0.027, 0.042]	0.037	[0.036, 0.037]	0.029	0.042	0.002	0.006
$T_{m}$	0.028	[0.021, 0.034]	0.030	[0.029, 0.030]	0.023	0.034	0.001	0.003
$R_{\mathrm{RO}}$	0.025	[0.018, 0.031]	0.026	[0.025, 0.026]	0.020	0.030	–	–
SEC	0.009	[0.005, 0.013]	0.009	[0.009, 0.009]	0.007	0.010	0.000	0.001
$c_{\mathrm{net}}$	0.006	[0.003, 0.009]	0.006	[0.006, 0.006]	0.003	0.009	0.005	0.017
Φ	0.003	[0.001, 0.006]	0.004	[0.004, 0.004]	0.003	0.004	0.000	0.001
ϕ	0.000	[−0.001, 0.001]	0.001	[0.001, 0.001]	0.001	0.001	0.000	0.000
$R_{\mathrm{UF}}$	0.000	[0.000, 0.000]	0.000	[0.000, 0.000]	0.000	0.000	0.000	0.000
Sum of $S_{j}$	0.982				0.979	0.986	0.989	0.992

**Table 9:** Effect of dependent membrane operating and cost parameters at v=1 USD/m^3^. Group indices are multidimensional total-effect Sobol indices at κ=0.5 [40], $N=2^{14}$. The membrane group is $R_{\mathrm{UF}}$, $R_{\mathrm{RO}}$, Φ, SEC and $T_{m}$ (operating), plus $c_{\mathrm{cap}}$ and $c_{\mathrm{mem}}$ (operating and cost), and the placement group is ε, $\rho_{u}$ and $\theta_{E}$. Ensemble statistics use the 200,000 paired draws with rank correlations induced by the method of Iman and Conover [39], and median $\kappa^{*}$ uses 50,000 draws. $\rho_{S}=0$ is the independent baseline.

Coupling		Configuration B: Group $S_{T}$			A: $S_{T}$	B: P(NB<0)		$P({\mathrm{NB}}_{B}>{\mathrm{NB}}_{A})$	B: Median
	$\rho_{S}$	Membrane	Placement	$\lambda_{p}$	Membrane	κ=0.5	κ=0.3	κ=0.5	$\kappa^{*}$
Operating	0.0	0.068	0.488	0.220	0.002	0.29%	3.3%	46.2%	12.2%
	0.3	0.113	0.465	0.209	0.002	0.40%	3.7%	46.7%	12.2%
	0.6	0.154	0.444	0.200	0.003	0.58%	4.1%	47.1%	12.2%
	0.9	0.190	0.426	0.191	0.004	0.76%	4.4%	47.5%	12.2%
Operating and cost	0.0	0.203	0.488	0.220	0.014	0.29%	3.3%	46.2%	12.2%
	0.3	0.357	0.397	0.178	0.023	0.97%	5.0%	47.4%	12.2%
	0.6	0.461	0.336	0.149	0.031	1.9%	6.8%	48.3%	12.2%
	0.9	0.535	0.293	0.129	0.040	3.1%	8.7%	49.0%	12.2%

**Table 10:** Break-even outputs for the five constructed district scenarios at v=1 USD/m^3^ and targeted connection order. Net benefit, cost per cubic meter of potable input avoided $u_{M}$, saving $\Delta_{M}$, and $B^{*}$ refer to κ=0.5. The quantity P(NB<0) is the share of the paired draws with negative net benefit at that coverage, with $\lambda_{p}$ centered on the scenario value. $v^{†}$ is the configuration crossover value of water with the scenario’s $\lambda_{p}$, and $v_{0.15}^{†}$ is the crossover with $\lambda_{p}=0.15$ in every scenario, which isolates the effect of sector composition. Volumes follow the proportional expansion of Equation (4).

Mix	Cfg.	$\kappa^{*}$	$\kappa^{{}^{\circ}}$	NB (kUSD/yr)	$u_{M}$ (USD/m^3^)	$\Delta_{M}$ (pp)	$B^{*}$ (USD/m^3^)	P(NB<0)	$v^{†}$ ($v_{0.15}^{†}$) (USD/m^3^)
Reference mixed district	A	11.7%	96.1%	706	0.48	11.52	0.764	<0.01%	0.981 (0.981)
	B	11.5%	94.9%	726	0.70	20.36	0.873	0.30%	
Residential periphery	A	13.7%	94.3%	568	0.52	9.74	0.737	0.07%	1.099 (1.140)
	B	14.9%	92.8%	502	0.73	15.18	0.850	1.3%	
Central district, pilgrimage peak	A	14.0%	96.5%	557	0.52	10.20	0.704	0.05%	0.976 (0.943)
	B	13.5%	95.1%	580	0.73	18.81	0.831	0.81%	
Healthcare and institutional campus	A	20.4%	96.9%	310	0.62	7.43	0.591	1.6%	0.852 (0.805)
	B	15.6%	95.3%	472	0.75	17.33	0.799	1.7%	
Landscaped district	A	5.5%	97.7%	1806	0.34	21.81	0.953	0.00%	1.386 (1.473)
	B	6.7%	95.9%	1426	0.62	29.69	0.982	<0.01%	

**Table 11:** Volumes of the sequencing frame at the reference mix, each program incremental to the status quo. The membrane configurations refer to κ=0.5 with targeted connection order, and $u_{M}$ is the cost per cubic meter of potable input avoided, with 5th to 95th percentiles over the paired draws. Programs P and T are reported as volumes only: their cost boundaries are not matched with those of the reuse scheme, so no unit-cost ranking is made.

Program	Volume	Saving	$u_{M}$ (USD/m^3^)		Share with
	(Mm^3^/yr)	(pp)	Nominal	Median [5th, 95th]	$u_{M}<1$ USD/m^3^
P: pressure-management expansion	0.388	3.30	–	–	–
T: tariff or fixture program	0.600	5.10	–	–	–
A: ultrafiltration reuse	1.355	11.52	0.48	0.48 [0.36, 0.67]	>99.9%
B: ultrafiltration with reverse osmosis	2.395	20.36	0.70	0.71 [0.57, 0.87]	99.7%
A after T and P	1.226	10.42	0.51	0.52 [0.38, 0.72]	>99.9%
B after T and P	2.168	18.42	0.73	0.75 [0.61, 0.92]	99.1%

**Table 12:** Maximum affordable annual cost $vV_{M}$ (panel A) and net avoided energy (panel B) at the nominal parameter set, κ=0.5 and $D=10\;{\mathrm{Mm}}^{3}$/yr. The nominal annualized costs are 649 kUSD/yr for Configuration A and 1669 kUSD/yr for Configuration B. Panel B subtracts the reclamation plant’s own electricity consumption, 0.24 and 1.02 kWh/m^3^ of product, and reclaimed-network pumping $e_{d}$ per cubic meter of product from the energy of the displaced potable supply. The two panels are independent sweeps.

A. Maximum Affordable Annual Cost (Thousand USD/Year)				
**Water value (USD/m^3^)**	**A**	**B**	**A after T, P**	**B after T, P**
0.5	677	1198	613	1084
1.0	1355	2395	1226	2168
2.0	2710	4791	2452	4335
B. Net avoided energy (GWh/year)				
**Displaced specific energy (kWh/m^3^)**	**A, $e_{d}=0$**	**B, $e_{d}=0$**	**A, $e_{d}=0.3$**	**B, $e_{d}=0.3$**
3.0	3.75	4.82	3.35	4.12
4.5	5.78	8.41	5.39	7.72
6.0	7.81	12.00	7.42	11.31

**Table 13:** Placement, connection, network and scale scenarios at v=1 USD/m^3^. NB and $\kappa^{*}$ are nominal values, with NB at κ=0.5. The quantity P(NB<0) is the share of the 200,000 paired draws with negative net benefit at κ=0.5, and $v^{†}$ is the crossover at κ=0.5. A dash indicates a quantity not computed for that row. Settings are in Appendix D.

Scenario	Configuration A			Configuration B			
	NB (kUSD/yr)	$\kappa^{*}$	P(NB<0)	NB (kUSD/yr)	$\kappa^{*}$	P(NB<0)	$v^{†}$
Baseline	706	11.7%	<0.01%	726	11.5%	0.29%	0.981
*Loss credit*							
No loss credit, $\eta_{L}=0$	503	14.9%	0.09%	367	18.2%	5.8%	1.154
Half loss credit, $\eta_{L}=0.5$	605	13.2%	0.01%	547	14.1%	1.2%	1.060
Eligibility							
Restrictive regulatory scenario	372	18.3%	0.74%	527	14.5%	0.94%	0.846
Permissive regulatory scenario	1086	8.4%	0.00%	924	9.5%	0.11%	1.160
Sector-specific multipliers	–	–	<0.01%	–	–	0.20%	–
Connection order, connection counting and network cost							
Power reach form $\kappa^{\tau }$	556	9.1%	0.01%	571	8.8%	0.65%	–
Half targeted, α=0.5	529	15.8%	0.01%	543	15.5%	0.79%	–
Untargeted, α=0	352	23.0%	–	359	22.8%	–	–
Whole non-residential connections	642	12.4%	0.05%	680	11.9%	0.60%	–
Whole connections in all sectors	310	17.9%	–	348	16.8%	–	–
Trunk main, 50% of network cost fixed	679	13.8%	0.01%	689	14.1%	0.52%	–
Convex network cost, γ=1.5	723	11.4%	<0.01%	748	11.0%	0.19%	–
Concave network cost, γ=0.7	693	12.2%	–	709	12.1%	–	–
Treatment capacity and zone size							
Plant built for full coverage	652	18.1%	–	492	31.7%	–	–
Two treatment trains	652	14.0%	–	492	18.2%	–	–
Four treatment trains	706	12.0%	–	726	12.2%	–	–
Zone of 2.5 Mm^3^/yr, *F* fixed	−48	68.6%	–	−43	66.0%	–	0.981
Zone of 2.5 Mm^3^/yr, $F\propto D^{0.5}$	102	25.4%	–	107	24.8%	–	–
Zone of 40 Mm^3^/yr, *F* fixed	3725	2.8%	–	3805	2.7%	–	0.981
Zone of 40 Mm^3^/yr, $F\propto D^{0.5}$	3425	5.7%	–	3505	5.5%	–	–
Seasonal placement, compliance and pumping							
Monthly balance, no storage	674	12.2%	–	641	12.6%	–	1.033
Monthly balance, one month of storage	797	10.7%	–	859	10.1%	–	0.943
Seasonal ensemble	–	11.6%	<0.01%	–	12.0%	0.19%	1.027
Excursions, 6 of 3 days, 50 kUSD each, diverted	340	–	–	308	–	–	–
Excursions, 12 of 7 days, 50 kUSD each, diverted	−205	–	–	−425	–	–	–
Excursions, 12 of 7 days, 50 kUSD each, retreated	39	–	–	−172	–	–	–
Pumping charged, $e_{d}=0.4$ kWh/m^3^	675	–	–	671	–	–	1.004

**Table 14:** Membrane-process scenarios at v=1 USD/m^3^. Columns as in Table 13. Fouling growth is the annual increase of the fouling penalty as a share of its initial value. The aging rows report the most adverse of 54 salt-passage combinations. $v^{†}$, configuration crossover value of water (USD/m^3^) above which Configuration B has the higher net benefit. UF, ultrafiltration; RO, reverse osmosis.

Scenario	Configuration A			Configuration B			
	NB (kUSD/yr)	$\kappa^{*}$	P(NB<0)	NB (kUSD/yr)	$\kappa^{*}$	P(NB<0)	$v^{†}$
Baseline	706	11.7%	<0.01%	726	11.5%	0.29%	0.981
Concentrate management (Configuration B only)							
$c_{b}=0.00$ USD/m^3^	–	–	–	884	9.9%	0.03%	0.830
$c_{b}=0.60$ USD/m^3^	–	–	–	569	13.7%	2.2%	1.132
$c_{b}=1.00$ USD/m^3^	–	–	–	359	18.4%	13.7%	1.333
$c_{b}=2.00$ USD/m^3^	–	–	–	−165	n.a.	68.9%	1.837
Fouling and element life							
Fouling penalty Φ=1.0 (no double counting)	715	11.6%	<0.01%	765	11.0%	0.17%	–
Fouling penalty Φ=1.5	694	11.9%	–	671	12.1%	–	–
Fouling growth 20%/yr	665	12.3%	–	589	13.3%	–	–
Fouling growth 20%/yr and element life halved	651	12.5%	0.01%	473	15.5%	6.3%	–
Element life at lower support (A 5 yr, B 3 yr)	685	12.0%	–	538	14.2%	–	–
Element life at upper support (A 12 yr, B 10 yr)	718	11.6%	–	798	10.7%	–	–
Ultrafiltration element life 1.8 yr (A)	584	13.5%	–	–	–	–	0.863
Separate UF and RO element terms (B)	–	–	–	752	11.2%	0.15%	–
Feed salinity							
400 mg/L	1503	6.4%	–	744	11.3%	–	A always
1500 mg/L	706	11.7%	–	712	11.6%	–	0.995
2500 mg/L	339	19.3%	–	650	12.4%	–	0.797
3500 mg/L	339	19.3%	–	589	13.3%	–	0.837
Salt-passage aging (Configuration B, most adverse case)							
Replace elements at the class limit	–	–	–	455	–	–	–
Run to declared life, out-of-class product restricted	–	–	–	453	–	–	–

**Table 15:** Monitoring and governance functions assumed in the fixed annual cost *F*, with the measured quantity and the acceptance criterion. Normative requirements are safety constraints, not targets to be optimized. Provisional targets are unvalidated engineering choices that require hazard analysis and authority approval before use as acceptance criteria.

Function	Measured Quantity	Acceptance Criterion
Cross-connection control	Confirmed physical connections between the potable and reclaimed networks	Normative requirement: 0
Product-quality verification	Online turbidity and conductivity, with periodic pathogen and micropollutant assay against the assumed quality class	Normative requirement: no delivery outside the approved class; out-of-class product diverted, stored for retreatment or retreated
Membrane integrity	Pressure-decay or particle-counting test on the ultrafiltration stage; conductivity profiling of reverse-osmosis vessels	Provisional target: integrity test at least daily
Approved end use	Register of connections, their approved uses and the quality class supplied	Normative requirement: no supply to an unapproved use
Fouling and cleaning	Normalized permeate flux, transmembrane pressure, cleaning interval, chemical consumption	Provisional target: cleaning interval consistent with the declared Φ
Element condition	Element age, replacement events, salt passage trend	Provisional target: element life at or above the value used in the business case
Concentrate management	Volume, composition and disposal route of reverse-osmosis concentrate	Normative requirement: disposal within permit
Placement and rebound	Delivered reclaimed volume by sector against the potable volume it replaced	Reported; used to estimate $\rho_{u}$ in operation

**Table 16:** Measurement protocol for a district pilot in an illustrative setting such as Madinah, ordered by the total-effect Sobol index of Configuration B at κ=0.5 under independent inputs. Pilot stages are Stage 0, baseline characterization; Stage 1, bench and pilot membrane testing on local effluent; Stage 2, demonstration supply to a bounded set of approved end uses; Stage 3, multi-district operation.

Symbol	Quantity	How the Pilot Measures It	$S_{\mathrm{Tj}}$	Stage
$\lambda_{p}$, $\eta_{L}$	Potable real losses and the share avoided	Standard water balance [32] with night-flow validation per district metered area, over a full year including the hot season; response of real losses to reduced throughput	0.220	0
$\theta_{E}$	Eligible-demand concentration	Lorenz curve of eligible demand across candidate connections, from metered consumption and an end-use audit, together with the connection order that topology and regulation allow	0.170	0
$\rho_{u}$	Unplaced product share	Monthly reconciliation of product water against metered deliveries to approved uses, over a full irrigation and cooling cycle, with the storage available	0.168	2
ε, $e_{i}$	Reuse eligibility	Regulatory determination of approved end uses per quality class, combined with a sector end-use audit of cooling, irrigation and flushing demand	0.155	0
$c_{\mathrm{cap}}$, $c_{\mathrm{mem}}$	Plant and element capital	Procurement quotations for both trains, itemized for ultrafiltration and reverse-osmosis elements separately from civil and mechanical works	0.100, 0.037	0–1
*F*, $c_{\mathrm{net}}$	Fixed and network cost	Annualized cost ledger for compliance, monitoring and staff; reclaimed network and service connection cost per connection	0.093, 0.006	0
$T_{m}$, Φ	Element life and fouling penalty	Normalized flux decline, transmembrane pressure trend, cleaning interval, recovery of specific flux after cleaning and salt-passage trend, on local secondary effluent	0.030, 0.004	1
$R_{\mathrm{RO}}$, $R_{\mathrm{UF}}$	Stage recoveries	Feed, permeate, backwash and concentrate flow balance on the pilot train at the design flux, over a full fouling cycle	0.026, 0.000	1
SEC	Specific energy	Metered electricity per cubic meter of product across the fouling cycle	0.009	1
ϕ	Effluent return ratio	Metered influent to the district wastewater treatment works, by month, against potable supply to the same district	0.001	0
κ	Coverage (decision)	Connected and approved eligible connection equivalents divided by all eligible connection equivalents	–	0
–	Feed salinity	Total dissolved solids and ionic composition of the secondary effluent over a year, against the salinity limits of the intended uses	–	0
–	Product water quality	Turbidity, conductivity, total dissolved solids, boron, indicator pathogens and a screening panel of micropollutants against the class assumed in Table 1; frequency and duration of out-of-class events	–	1–2
$c_{b}$	Concentrate	Volume, composition, permitted disposal route and cost for the reverse-osmosis concentrate	–	0–1
σ, τ, *r*	Pilot variance	Residual and between-cluster variation and temporal correlation of cluster-period consumption (Appendix F)	–	0

**Table 17:** Staged evaluation path for a polymeric membrane reuse scheme, with the scope, duration and required exit evidence for each stage.

Stage	Duration	Scope	Exit Evidence
0	0–6 months	Governance charter, effluent quantity and quality characterization, sector end-use audit, regulatory determination of approved uses per quality class, water balance, cost ledger and network costing	Measured $\lambda_{p}$, ϕ, $\bar{e}$, $\theta_{E}$, *F*, $c_{\mathrm{net}}$ and an initial position relative to $B^{*}$ and $\kappa^{*}$
1	7–18 months	Bench and pilot membrane testing on local secondary effluent for both configurations, through at least one full fouling and cleaning cycle	Measured *R*, SEC, flux decline and cleaning interval, product quality against the assumed class, concentrate composition and a quoted unit treatment cost *B*
2	19–36 months	Demonstration supply to a bounded set of approved end uses in one district, with metered delivery	Measured $\rho_{u}$ over a full irrigation cycle, realized displacement of potable consumption, element condition trend, and no cross-connection or out-of-class delivery event
3	37+ months	Multi-district operation and staged network extension toward $\kappa^{{}^{\circ}}$	Independent audit, annual quality and safety review, and revised break-even position from operating data

**Table 18:** Polymer membrane properties, the model parameters through which they act, and their computed effect on the decision variables at the nominal set, κ=0.5 and v=1 USD/m^3^. Material evidence is from the cited studies, and the effects are model outputs (Section 4.10), not measurements. $v^{†}$, configuration crossover value of water (USD/m^3^) above which Configuration B has the higher net benefit. PES, polyethersulfone; PVDF, polyvinylidene fluoride; UF, ultrafiltration; RO, reverse osmosis.

Property and Evidence	Material Data	Model Parameter	Computed Effect
Oxidant and alkaline tolerance of the UF polymer [19,20]	PES: permeability 3.8–5.8 times higher and humic-acid retention 59% to 20–37% after 500 g·h/L hypochlorite; PVDF: modeled life 1.8–7 yr under NaOH cleaning	UF element life $T_{m}$ (A; UF elements of B); integrity and excursions	UF life 1.8–7 yr: NB of A 584–701 kUSD/yr, $\kappa^{*}$ 13.5–11.8%, $v^{†}$ 0.86–0.98 USD/m^3^; excursions: Table 13
Reverse-osmosis element lifespan [16]	Often 5–10 yr	RO element life $T_{m}$ (B)	3, 5, 10 yr: NB of B 538, 687, 798 kUSD/yr; $\kappa^{*}$ 14.2, 11.9, 10.7%; $v^{†}$ 1.16, 1.02, 0.91 USD/m^3^
Fouling resistance of the membrane surface [4,15,16]	Fouling raises pressure, energy and cleaning frequency	Fouling penalty Φ and its growth	Φ 1.0–1.5: NB of B 765–671 kUSD/yr; growth 20%/yr with halved life: 473 kUSD/yr, $\kappa^{*}$ 15.5%
Scaling at high RO recovery [17,18,36]	Concentrate 4–10 times feed at 75–90% recovery; calcium phosphate limits recovery in reuse	RO recovery $R_{\mathrm{RO}}$; concentrate ratio	$R_{\mathrm{RO}}$ 0.74–0.88: $v^{†}$ 1.06–0.92 USD/m^3^, $\kappa^{*}$ of B 12.6–10.7%
Salt rejection of polyamide and its loss under oxidants [2,16]	Rejection above 99% for current elements; chlorine attack on amide and aromatic groups reduces rejection	Salt passage and its growth; quality class and $\bar{e}$ of B	Worst of 54 aging cases: NB of B 453–455 kUSD/yr; no case binding
Salt passage of UF (none) relative to irrigation limits [14]	No restriction below 450 mg/L, severe above 2000 mg/L	Eligibility of A by feed salinity	400 mg/L: A dominates; 2500 mg/L: $v^{†}$ 0.80 USD/m^3^, $\kappa^{*}$ of A 19.3%
Polymer fragment release under aggressive cleaning [11]	Release from UF systems under backwash and cleaning	Not represented	Quality question outside the present class framing

**Table 19:** Direction of the bias introduced by each simplification for the incremental net benefit of the reuse scheme, and where this paper bounds it.

Simplification	Effect on Net Benefit	Treatment in This Paper
Real losses that do not scale with system input	Overstates the scheme	Bounded with $\eta_{L}=0$ (Table 6 and Table 13)
Reclaimed-network pumping energy and its cost	Overstates the scheme	Range of $e_{d}$; ranking and sign of energy savings robust (Section 4.9)
Storage or aquifer recharge to absorb seasonal mismatch	Understates the volume; its cost is omitted	Volume effect in Appendix E; cost not modeled
Progressive irreversible fouling within an element’s life	Overstates the scheme	Scenario in Table 14
Loss of salt rejection with element age, and its effect on eligibility	Overstates the scheme	Scenario in Table 14
Dependence among membrane operating and cost parameters	Understates the variance for Configuration B	Group indices in Table 9
Idle capacity where plant construction leads network build-out	Overstates the scheme	Discrete-train scenario in Table 13
Effluent intake expanded in proportion to the reach (Equation (4))	Understates the volume where effluent supply binds	Full-access variant in Section 4.4 and Section 4.8
Quality excursions requiring diversion or retreatment	Overstates the scheme	Scenario in Section 4.10
Feed salinity effects on energy, recovery and eligibility	Either direction	Scenarios in Table 14
Network topology, trunk mains and clustering of large users	Either direction	Alternative cost structures in Table 13
Co-benefits of reuse such as deferred desalination capacity and nutrient value [56]	Understates the scheme	Not modeled
Public acceptance and end-use approval delay [27]	Overstates the scheme	Not modeled

**Figure A1:**
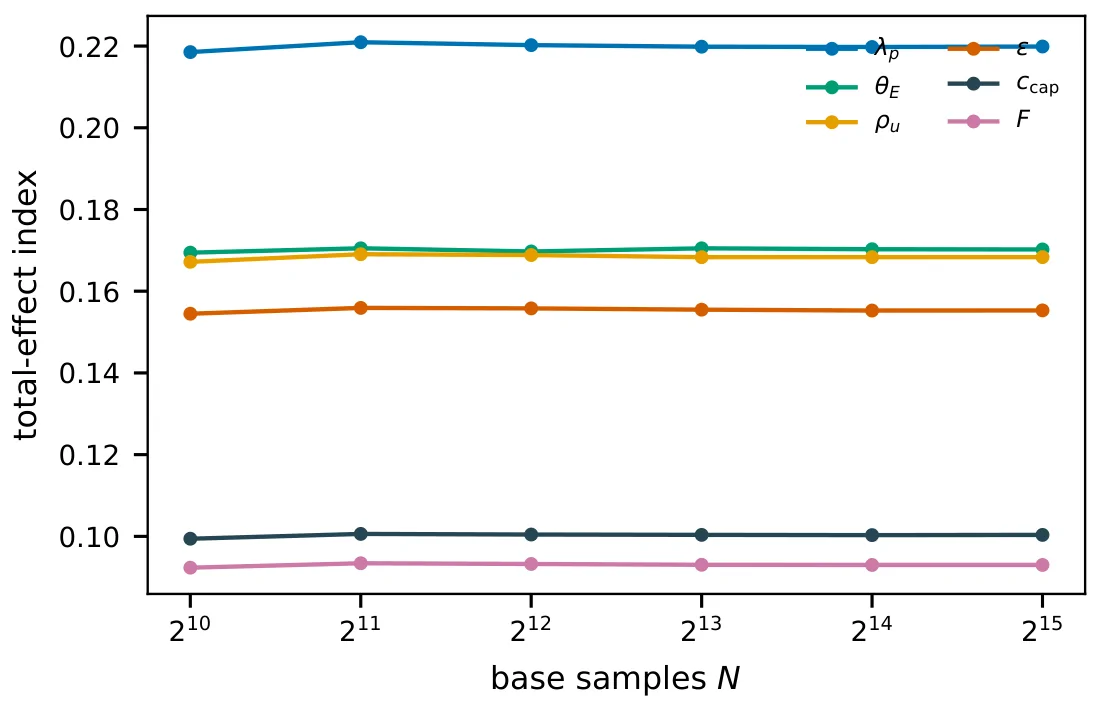
Within-sequence stabilization of total-effect Sobol indices of annual net benefit for Configuration B (κ=0.5, v=1 USD/m^3^, targeted order) over prefixes of one scrambled design, for the six most influential inputs.

**Figure A2:**
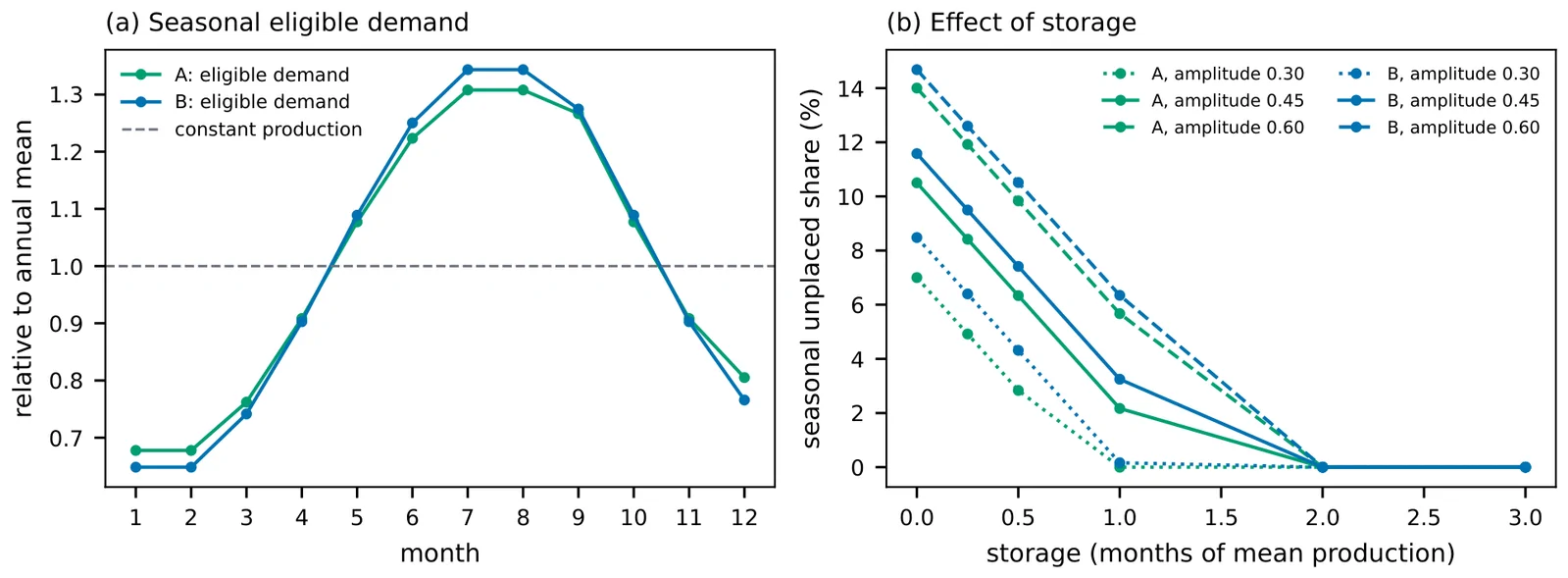
(**a**) Monthly eligible demand relative to its annual mean at the reference mix, with the pilgrimage months in September and December, against constant production. (**b**) Seasonal unplaced share against storage for three irrigation amplitudes, averaged over the twelve pilgrimage positions. Declared profiles, not local data.

## Data Availability

Every reported number, table entry, and figure of the analysis can be regenerated with analysis.py (Supplementary File S1), which writes results.json (Supplementary File S2), in the environment pinned in requirements-lock.txt (Supplementary File S4). The script verify_results.py (Supplementary File S3) re-derives the closed-form results independently. No third-party dataset was used, and all inputs are declared in the manuscript. Further inquiries can be directed to the corresponding author.

## Supplementary Materials

The following supporting information can be downloaded at: https://www.mdpi.com/article/10.3390/polym18212773/s1, Supplementary File S1: analysis.py, which regenerates the reported analysis. Supplementary File S2: results.json, which contains the numerical output and the software versions found at runtime. Supplementary File S3: verify_results.py, an independent re-derivation of the closed-form results from the stored nominal inputs. Supplementary File S4: requirements-lock.txt, the exact software environment.

## Abbreviations

The following abbreviations are used in this manuscript:

CRF	Capital Recovery Factor
CV	Coefficient of Variation
EVPI	Expected Value of Perfect Information
EVPPI	Expected Value of Partial Perfect Information
MDE	Minimum Detectable Effect
NB	Net Benefit
PES	Polyethersulfone
pp	Percentage Points
PVDF	Polyvinylidene Fluoride
RO	Reverse Osmosis
SEC	Specific Electrical Energy
TDS	Total Dissolved Solids
TN	Truncated Normal
UF	Ultrafiltration
USD	United States Dollar

## Appendix A. Derivation of the Break-Even Conditions

Write $A_{v}=v\left(1-\rho_{u}\right)\;m_{L}$ for the value of a cubic meter of product water, in USD/m^3^, with the loss multiplier $m_{L}$ of Equation (5), so that every result below holds for any loss credit $\eta_{L}\in \left[0,1\right]$ (item (x)). By Equations (4)–(6) and (8), the net benefit takes the form of Equation (A1):

(A1) $$\mathrm{NB}\left(\kappa \right)=\left(A_{v}-B\right)\;V^{\max}\;\psi \left(\kappa ;\theta_{E}\right)-F-c_{\mathrm{net}}M_{e}\kappa =K\;\psi \left(\kappa ;\theta_{E}\right)-F-c_{\mathrm{net}}M_{e}\kappa ,$$

with $K=\left(A_{v}-B\right)V^{\max}$.

(i) At fixed κ, Equation (A1) is affine in *B*. Solving NB=0 for *B* gives Equation (11). It is also affine in ${\bar{e}}_{\varepsilon }$ while demand binds in Equation (2), which is why the break-even contour of Figure 3 is a straight line.

(ii) Only the term $\mathrm{CRF}\left(i,T_{m}\right)c_{\mathrm{mem}}/u$ of Equation (7) depends on $T_{m}$, and it is strictly decreasing in $T_{m}$. Setting $B=B^{*}$ and solving for that term gives the first equality of Equation (12). Inverting $\mathrm{CRF}\left(i,T\right)=i{\left(1+i\right)}^{T}/\left[{\left(1+i\right)}^{T}-1\right]$ for *T* with $x={\left(1+i\right)}^{T}$ gives x=CRF/(CRF−i) and hence the second equality, which requires $\mathrm{CRF}(i,T_{m}^{*})>i$. If the required capital recovery factor does not exceed *i*, then even an infinitely long element life leaves $B>B^{*}$, and no element life makes the scheme economic. If the scheme is economic at $T_{m}\to 0^{+}$, it is economic for every $T_{m}$, and the threshold is reported as not binding.

(iii) For $\theta_{E}=1$, ψ=κ and $\mathrm{NB}\left(\kappa \right)=\kappa \left(K-c_{\mathrm{net}}M_{e}\right)-F$. Its root is Equation (13), and the root lies in (0,1] if and only if $K-c_{\mathrm{net}}M_{e}\geq F$.

(iv) For $\theta_{E}>1$, $\psi^{\prime \prime }\left(\kappa ;\theta_{E}\right)=-\theta_{E}\left(\theta_{E}-1\right){\left(1-\kappa \right)}^{\theta_{E}-2}<0$ on (0,1). If K>0, then NB is strictly concave, so it has at most two roots and a unique maximizer on [0,1]. Since NB(0)=−F<0, the lower root, if it exists, is the break-even coverage $\kappa^{*}$. Setting ${\mathrm{NB}}^{\prime }\left(\kappa \right)=0$ gives Equation (14). If K≤0, then NB is non-increasing and negative throughout, and no coverage is cost-effective.

(v) With Programs T and P expanded first, the eligible demand and the effluent stream both scale by (1−r), so $V^{\max}$ scales by (1−r), and the remaining potable loss share is $\lambda_{p}\left(1-\beta_{r}\right)$. Substituting gives Equation (9) and the corresponding thresholds. No volume is credited twice, because the reuse scheme is credited only with displaced potable demand and the losses avoided in supplying it, while pressure management is credited only with losses avoided in the potable network that remains in service.

(vi) Every factor of $V_{M}$ is non-negative, so $V_{M}\geq 0$ for every admissible parameter set and the scheme never reduces supply within the model. Its economic evaluation is carried out entirely by the comparison of $vV_{M}$ with $Z_{M}$.

(vii) $V_{M}$ and $Z_{M}$ do not depend on *v*, so ${\mathrm{NB}}_{B}-{\mathrm{NB}}_{A}=v\left(V_{M,B}-V_{M,A}\right)-\left(Z_{M,B}-Z_{M,A}\right)$ is affine in *v*. When $V_{M,B}>V_{M,A}$, its unique root is Equation (15), and Configuration B has the higher net benefit exactly when $v>v^{†}$. Applied to each paired draw, the same expression gives the distribution of the crossover over the ensemble.

(viii) For a given configuration, NB(κ)≥0 if and only if $v\geq Z_{M}\left(\kappa \right)/V_{M}\left(\kappa \right)=u_{M}\left(\kappa \right)$. Some coverage is therefore cost-effective if and only if $v\geq \min_{\kappa }u_{M}\left(\kappa \right)=v^{*}$, and the probability of cost-effective deployment at value *v* over the ensemble is $P(v^{*}\leq v)$. We evaluate the minimum on a grid of 500 coverages.

(ix) By Equation (2), the supply term binds if and only if $R\phi D<{\bar{e}}_{\varepsilon }D$, that is, $R\phi <{\bar{e}}_{\varepsilon }$. Since ${\bar{e}}_{\varepsilon }$ of Equation (1) is non-decreasing in ε and bounded by $\sum_{i}w_{i}\min\left\{1,\varepsilon e_{i}\right\}$, the smallest eligibility scale at which supply binds solves ${\bar{e}}_{\varepsilon }=R\phi$. For the reference mix and nominal recoveries, it is 4.47 for Configuration A and 1.89 for Configuration B.

(x) The loss credit $\eta_{L}$ enters only through $m_{L}$ in $A_{v}$, and through $m_{L|U}$ in Equation (9). With full credit, $m_{L}=1/\left(1-\lambda_{p}\right)$. With $\eta_{L}=0$, $m_{L}=1$ and the avoided input equals the placed volume.

(xi) Full effluent access from the first phase (Section 3.4) gives the product water $W^{\mathrm{full}}\left(\kappa \right)=\min\left\{R\phi D,\;\psi \left(\kappa ;\theta_{E}\right)\;{\bar{e}}_{\varepsilon }D\right\}$. It equals $\psi V^{\max}$ of Equation (4) whenever ${\bar{e}}_{\varepsilon }\leq R\phi$. When ${\bar{e}}_{\varepsilon }>R\phi$, let $\kappa_{s}$ solve $\psi \left(\kappa_{s};\theta_{E}\right)=R\phi /{\bar{e}}_{\varepsilon }$. For $\kappa \leq \kappa_{s}$, $\mathrm{NB}=K^{\prime }\psi \left(\kappa ;\theta_{E}\right)-F-c_{\mathrm{net}}M_{e}\kappa$ with $K^{\prime }=\left(A_{v}-B\right)\;{\bar{e}}_{\varepsilon }D$, so Equations (11), (13) and (14) apply with $V^{\max}$ replaced by ${\bar{e}}_{\varepsilon }D$. For $\kappa >\kappa_{s}$, $\mathrm{NB}=\left(A_{v}-B\right)R\phi D-F-c_{\mathrm{net}}M_{e}\kappa$ decreases in κ. For $A_{v}>B$, net benefit therefore remains concave, and the optimal coverage is the smaller of $\kappa_{s}$ and the interior solution of Equation (14) with $K^{\prime }$.

## Appendix B. Numerical Stabilization of the Sobol Indices

Figure A1 shows the total-effect indices of net benefit for Configuration B at κ=0.5 recomputed on the first $2^{10},\ldots ,2^{15}$ rows of one scrambled Sobol design. Across these prefixes, the ranking of the six most influential inputs is stable from $N=2^{10}$, and no index of those inputs changes by more than 0.003 across the prefixes. This is a within-sequence stabilization check. It does not measure variability across independent scrambles and does not justify inferential confidence intervals.

## Appendix C. Evidence Basis and Translation of the Declared Parameters

Table A1 states, for each parameter of Table 3, the evidence consulted, how it was translated into the model quantity, and the resulting range. Where the evidence column reads “none specific”, the parameter is an author-declared planning choice and should be read as such throughout the paper.

**Table A1:** Evidence basis and translation of the declared parameters, with the literature consulted, the translation assumptions applied, and the resulting declared distributions.

Symbol	Evidence Consulted	Translation to the Model Quantity	Resulting Range
$\lambda_{p}$	Non-revenue water of the order of 30% of system input worldwide [33]; water-balance components [32]	Declared assumption that real losses are about half of non-revenue water for a mid-performing utility. Inferred indirectly, not from a local water balance; the share credited as avoided, $\eta_{L}$, is varied from 0 to 1	Mean 0.15, support 0.02–0.28
ϕ	None specific	Declared share of potable demand returning as collected sewage in an arid city with high outdoor use and partial sewer coverage	Mean 0.60, support 0.40–0.80
ε, $e_{i}$	Approved-use constraints for non-desalted and desalted reclaimed water; salinity limits on irrigation reuse in Saudi settings [13]; pathogen and resistance-gene carry-over in conventionally treated Saudi effluent [12]; national reuse assessment [26]	The weights in Table 2 encode an assumed regulatory determination of approved end uses per quality class, decomposed into end uses in Table A2. They are not measured shares, and no regulatory document, end-use survey, or elicitation record available to this study reports them	$\bar{e}=0.175$ (A) and 0.309 (B) at ε=1; ε mean 1.00, support 0.70–1.30
$\rho_{u}$	None specific	Declared share of product water not placed, covering seasonal mismatch between a continuous effluent stream and a seasonal irrigation load, reclaimed-network losses and periods when a connected use is not approved. A monthly balance with declared profiles (Appendix E) gives configuration-specific values used as a scenario	Mean 0.12, support 0.02–0.22
$\theta_{E}$	None specific	Declared concentration of eligible demand among connections. At the nominal value the first 20% of eligible connections hold 36% of eligible demand	Mean 2.0, support 1.0–3.0
Φ	Fouling raises transmembrane pressure and cleaning frequency in reuse service [15]; pretreatment selection is the principal control [22]	Declared multiplier on energy and cleaning cost, relative to the reference operating state of the cited specific energies and cleaning costs (Φ=1), which already includes their routine fouling and cleaning; Φ>1 is a margin for a less favorable local feed. No source reports a single equivalent multiplier	Mean 1.20, support 1.00–1.50
$R_{\mathrm{UF}}$, $R_{\mathrm{RO}}$	Overall recovery of microfiltration or ultrafiltration with reverse osmosis in municipal reuse service [5,25]; ultrafiltration recovery in tertiary service [7]; scaling as the key barrier to high reverse-osmosis recovery [36]	Ultrafiltration-stage recovery, product divided by feed, including backwash and cleaning losses, which return to the works; reverse-osmosis stage recovery declared so that the overall recovery $R_{\mathrm{UF}}R_{\mathrm{RO}}$ of Configuration B spans the literature range of 0.60–0.88	$R_{\mathrm{UF}}$: mean 0.92, support 0.86–0.97. $R_{\mathrm{RO}}$: mean 0.815, support 0.70–0.90; overall B: 0.60–0.87
SEC	0.84 kWh/m^3^ for a reverse-osmosis-based municipal reuse train and 0.30 for a train without desalting [5]; 0.16 kWh/m^3^ for tertiary ultrafiltration on municipal wastewater [7]	Electrical energy per cubic meter of product for the membrane train only, excluding reclaimed-network distribution, at the reference operating state of the cited trains. Support widened around the reported central values	A: mean 0.20, support 0.10–0.35. B: mean 0.85, support 0.45–1.25
$T_{m}$	Element service life and end-of-life handling in reverse-osmosis service [21]; reverse-osmosis lifespans often limited to 5–10 years [16]; fouling as the principal determinant of replacement interval [15]; accelerated aging of PVDF ultrafiltration under alkaline cleaning [20]	Declared service life consistent with the cited range for an adequately pretreated train; ultrafiltration life set longer than reverse-osmosis life. For Configuration B, a single effective life of the ultrafiltration and reverse-osmosis elements; separate lives are a scenario	A: mean 8.0, support 5.0–12.0 yr. B: mean 6.0, support 3.0–10.0 yr
$c_{\mathrm{cap}}$, $c_{\mathrm{mem}}$	Levelized cost of 0.54 USD/m^3^ for a reverse-osmosis-based reuse train and 0.40 for one without desalting [5]; unit cost against scale for reuse processes [23]	Declared split of overnight capital into plant and element components on an installed annual-capacity basis, set so that the nominal *B* of Equation (7) for Configuration B is close to the cited reverse-osmosis-train cost and that of Configuration A lies below the cited cost of a carbon-based train without desalting. Generic literature calibration, not Saudi procurement	A: $c_{\mathrm{cap}}$ 0.70–1.40, $c_{\mathrm{mem}}$ 0.12–0.26. B: $c_{\mathrm{cap}}$ 1.60–3.20, $c_{\mathrm{mem}}$ 0.25–0.60 USD per m^3^/yr
$c_{b}$	Inland reverse-osmosis concentrate has no low-cost sink and requires advanced treatment or disposal [6]; adding zero liquid discharge to a facility that discharges concentrate to surface water would approximately double the levelized cost of water [5]	Declared planning cost per cubic meter of reverse-osmosis concentrate for an inland site, not evidenced by a local permit or quotation; zero for Configuration A, whose ultrafiltration backwash returns to the works	0.30 USD/m^3^ nominal, swept over 0–3.0
$c_{\mathrm{cip}}$, $c_{\mathrm{om}}$, $c_{\mathrm{net}}$, *F*	None specific	Declared planning values for cleaning chemicals, other operation and maintenance, reclaimed distribution per connection, and the standing compliance, monitoring and staffing cost of Table 15	See Table 3
*r*	Price elasticities well below unity in magnitude [28,29]	Declared response based on elasticities of −0.1 to −0.5 combined with a real price increase of 10–40%, or an assumed equivalent fixture response	Mean 0.06,support 0–0.20
$\beta_{r}$	Pressure management among the more cost-effective leakage measures [30]; network application results [31]	Declared network-wide real-loss reduction for an expanded program, used for the sequencing volumes only	Mean 0.22, support 0.07–0.37
*v*	Desalination production costs [42] and conveyance energy [43,45]	Planning scenarios for the marginal value of potable water; production cost is not necessarily the marginal value of avoided supply, so thresholds on *v* are scenario thresholds	0.5, 1.0, 2.0 USD/m^3^

## Appendix D. Declared Settings of the Scenario, Structural and Decision Analyses

This appendix lists every setting of the analyses in Section 3.9 and Section 4.10 that is not a baseline input of Table 3. All are declared values chosen to bracket plausible conditions, and none is a measurement. Table A2 decomposes the eligibility weights into the end uses that each quality class is assumed to serve; Table A3 gives the discrete regulatory scenarios; Table A4 the remaining settings; and Table A5 the full one-at-a-time conditions analysis of Section 4.3.

**Table A2:** End-use decomposition of the eligibility weights of Table 2, as shares of each sector’s potable demand. Flat uses are toilet flushing, cleaning, and washdown. Declared values.

Sector	Configuration A			Configuration B		
	Irrigation	Cooling	Flat	Irrigation	Cooling	Flat
Residential	0.06	0.00	0.04	0.06	0.00	0.04
Hospitality	0.06	0.00	0.04	0.06	0.15	0.04
Mosques	0.10	0.00	0.15	0.15	0.00	0.15
Healthcare	0.00	0.00	0.00	0.00	0.05	0.00
Commerce and institutions	0.00	0.00	0.10	0.00	0.25	0.10
Landscaping	0.55	0.00	0.00	1.00	0.00	0.00

**Table A3:** Discrete regulatory scenarios for the eligibility weights (declared). Restrictive: only irrigation, washdown, and cooling uses are approved, with no indoor toilet flushing. Permissive: flushing in dual-plumbed buildings and a broader set of irrigation uses are approved for the ultrafiltered product. Results are in Section 4.10 and Table 13.

Scenario	Cfg.	Resid.	Hosp.	Mosques	Health	Comm.	Landsc.
Restrictive	A	0.05	0.05	0.15	0.00	0.05	0.45
	B	0.05	0.20	0.15	0.05	0.30	0.90
Reference	A	0.10	0.10	0.25	0.00	0.10	0.55
	B	0.10	0.25	0.30	0.05	0.35	1.00
Permissive	A	0.15	0.15	0.35	0.00	0.15	0.70
	B	0.15	0.35	0.40	0.10	0.45	1.00

**Table A4:** Declared settings of the scenario, structural and decision analyses of Section 3.9 and Section 4.10, listed by analysis. All settings are author-declared values, not measurements. UF, ultrafiltration; RO, reverse osmosis; TDS, total dissolved solids.

Analysis	Declared Settings
Pairing	One draw of every input of the first block of Table 3 and of *r*, $\beta_{r}$ per realization, shared by both configurations; configuration-specific inputs from separate seeded streams
Conditions analysis	Each distributed input at its 5th and 95th percentiles; $\pi_{E}$ 0.03 and 0.12 USD/kWh; $c_{b}$ 0 and 0.60 USD/m^3^; *i* 0.03 and 0.10; *u* 0.75 and 0.95; $c_{\mathrm{om}}$ multiplied by 0.5 and 1.5; *D* 5 and 20 Mm^3^/yr with *F* fixed
Dependent inputs	Adverse-feed direction: $R_{\mathrm{UF}}$, $R_{\mathrm{RO}}$, $T_{m}$ decreasing; Φ, SEC increasing; the operating-and-cost variant adds $c_{\mathrm{cap}}$ and $c_{\mathrm{mem}}$ increasing. Pairwise Spearman correlation $\pm \rho_{S}$ with $\rho_{S}=0.3$, 0.6, 0.9
Decision metrics	Water values 0.40–2.50 USD/m^3^ in steps of 0.05; EVPPI groups: $\lambda_{p}$; $\left(\varepsilon ,\rho_{u},\theta_{E}\right)$; unit treatment costs $\left(B_{A},B_{B}\right)$; membrane-specific unit costs; plant capital; $\left(F,c_{\mathrm{net}}\right)$; tensor-product cubic splines with 24, 12 and 7 basis functions per dimension for one, two and three inputs
Sector-specific eligibility	$\varepsilon_{i}$ drawn independently for each sector from the distribution of ε, common to both configurations
Connection order	Partial targeting α=1, 0.75, 0.5, 0.25, 0; power form $\kappa^{\tau }$ matched at κ=0.2
Connection counting	Equivalents (baseline); every non-residential user in a sector with $e_{i}>0$ as a whole connection; every user as a whole connection
Network cost	Linear (baseline); trunk main costing 25% or 50% of the full-coverage network cost, incurred at the first connection; $c_{\mathrm{net}}M_{e}\kappa^{\gamma }$ with γ=0.7 or 1.5
Treatment capacity	Full-coverage capacity in 1, 2, 4 or 8 equal trains; capital charged on installed trains, at least one
Effluent access	Proportional expansion, Equation (4) (baseline); whole collected effluent available from the first phase, $W=\min\{R\phi D,\psi {\bar{e}}_{\varepsilon }D\}$; reference mix, landscaped district and permissive scenario
Zone size	D=2.5, 5, 10, 20, 40 Mm^3^/yr with *F* fixed, $F\propto D^{0.5}$, or $F\propto D^{0.5}$ and $c_{\mathrm{cap}}\propto D^{-0.25}$
Loss credit	$\eta_{L}=0$, 0.25, 0.5, 0.75, 1
Concentrate	$c_{b}=0$, 0.15, 0.30, 0.60, 1.0, 1.5, 2.0, 3.0 USD per m^3^ of concentrate; zero-liquid-discharge anchor: the $c_{b}$ that doubles *B* of Configuration B relative to $c_{b}=0$
Progressive fouling	Fouling penalty rising linearly with element age at k=0, 5, 10, 20% of its initial value per year, life-averaged $\Phi (1+kT_{m}/2)$; element life reduced by 0, 25 or 50%
Separate element terms (B)	UF elements of B: cost $c_{\mathrm{mem}}$ of A divided by $R_{\mathrm{RO}}$, life $T_{m}$ of A; RO elements: Cost U[0.08, 0.30] USD per m^3^/yr, life $T_{m}$ of B
Feed salinity	400, 1000 (reference), 1500, 2500, 3500 mg/L. RO specific energy $+\pi^{\prime }\Delta \mathrm{TDS}/\left[\left(1-R_{\mathrm{RO}}\right)R_{\mathrm{RO}}\eta \right]$ with $\pi^{\prime }=0.75$ bar per g/L and η=0.80; $R_{\mathrm{RO}}$ reduced by 0.035 per g/L above 1500 mg/L; eligibility of A equal to that of B at or below 450 mg/L, reference between 450 and 2000 mg/L, irrigation uses halved above 2000 mg/L [14]
Salt-passage aging (B)	Permeate TDS $={\mathrm{TDS}}_{\mathrm{feed}}\;SP_{0}{\left(1+g\right)}^{t}$; $SP_{0}=1$, 2, 3%; g=7, 15, 30%/yr; class limits 450 and 200 mg/L; feed 1000, 2500, 3500 mg/L. Policies: replace elements at the class limit, or run to the declared life with out-of-class product restricted to the uses of Configuration A
Seasonal balance	Appendix E
Compliance failures	2, 6 or 12 events per year of 1, 3 or 7 days; response cost 10 or 50 kUSD per event; out-of-class product diverted, or retreated at *B* per m^3^
Pumping	$e_{d}=0$–0.6 kWh per m^3^ of product distributed; cost $\pi_{E}e_{d}$
Pilot power	Appendix F

**Table A5:** One-at-a-time conditions at κ=0.5 and v=1 USD/m^3^: crossover value $v^{†}$ and break-even coverage $\kappa^{*}$ with each input at its low and high setting, all others nominal. Nominal: $v^{†}=0.981$ USD/m^3^, $\kappa^{*}=11.7\%$ (A) and 11.5% (B). Inputs are ordered by the swing of $v^{†}$.

Input	Applies to	Low; High Setting	$v^{†}$ Low; High	$\kappa_{A}^{*}$ Low; High	$\kappa_{B}^{*}$ Low; High
$c_{b}$	B	0; 0.6	0.830; 1.132	11.7%; 11.7%	9.9%; 13.7%
$c_{\mathrm{cap}}$	B	1.68; 3.12	0.842; 1.120	11.7%; 11.7%	10.0%; 13.5%
*i*	A and B	0.03; 0.1	0.873; 1.144	11.3%; 12.5%	9.9%; 14.9%
$\lambda_{p}$	A and B	0.0697; 0.23	1.073; 0.888	13.4%; 10.2%	14.5%; 9.1%
$\pi_{E}$	A and B	0.03; 0.12	0.922; 1.099	11.6%; 12.0%	10.7%; 13.4%
$\rho_{u}$	A and B	0.0464; 0.194	0.905; 1.070	10.5%; 13.3%	9.5%; 14.4%
$c_{\mathrm{mem}}$	B	0.268; 0.583	0.902; 1.059	11.7%; 11.7%	10.6%; 12.5%
$T_{m}$	B	3.8; 8.43	1.086; 0.931	11.7%; 11.7%	12.9%; 10.9%
$R_{\mathrm{RO}}$	B	0.74; 0.884	1.064; 0.918	11.7%; 11.7%	12.6%; 10.7%
*u*	A and B	0.75; 0.95	1.084; 0.954	12.2%; 11.6%	13.4%; 11.0%
$c_{\mathrm{om}}$ factor	A and B	0.5; 1.5	0.918; 1.043	11.3%; 12.2%	10.4%; 12.8%
ε	A and B	0.779; 1.22	0.986; 1.088	15.6%; 9.4%	15.3%; 10.2%
SEC	B	0.573; 1.13	0.936; 1.025	11.7%; 11.7%	10.9%; 12.0%
$c_{\mathrm{cap}}$	A	0.735; 1.36	1.015; 0.946	11.3%; 12.2%	11.5%; 11.5%
Φ	A and B	1.05; 1.36	0.960; 1.003	11.7%; 11.8%	11.1%; 11.8%
$c_{\mathrm{mem}}$	A	0.127; 0.253	0.995; 0.967	11.6%; 11.9%	11.5%; 11.5%
$T_{m}$	A	5.8; 10.4	0.968; 0.988	11.9%; 11.6%	11.5%; 11.5%
$c_{\mathrm{net}}$	A and B	66; 174	0.973; 0.989	11.5%; 12.0%	11.2%; 11.8%
SEC	A	0.127; 0.282	0.988; 0.974	11.7%; 11.8%	11.5%; 11.5%
$\theta_{E}$	A and B	1.26; 2.74	0.986; 0.979	18.4%; 8.6%	18.1%; 8.4%
ϕ	A and B	0.473; 0.727	0.981; 0.981	11.7%; 11.7%	11.5%; 11.5%
*F*	A and B	165 kUSD/yr; 435 kUSD/yr	0.981; 0.981	6.3%; 17.6%	6.1%; 17.2%
$R_{\mathrm{UF}}$	A and B	0.887; 0.952	0.981; 0.981	11.7%; 11.7%	11.5%; 11.5%
*D*	A and B	5 Mm^3^/yr; 20 Mm^3^/yr	0.981; 0.981	25.4%; 5.7%	24.8%; 5.5%

## Appendix E. Monthly Balance of Product Water and Eligible Demand

The annual model compresses seasonal mismatch into $\rho_{u}$. The monthly balance makes it explicit with declared profiles. Monthly eligible demand of configuration *c* is the sum over sectors of the end-use volumes of Table A2 at the reference mix, each multiplied by a monthly index with annual mean 1. Irrigation follows $1+a_{\mathrm{irr}}\cos\left[2\pi \left(m-6.5\right)/12\right]$ and cooling-tower makeup $1+a_{\mathrm{cool}}\cos\left[2\pi \left(m-6.5\right)/12\right]$, with m=0 for January, so that both peak between July and August. Flat uses are constant, except that flat uses in hospitality and mosques rise by 40% in two pilgrimage months three months apart, Ramadan and Dhu al-Hijjah, renormalized to an annual mean of 1. Because the Islamic calendar moves about 11 days earlier each solar year, the pilgrimage months move through the seasons, and all twelve monthly positions are evaluated. The declared amplitudes are $a_{\mathrm{irr}}=0.45$, with 0.30 and 0.60 as alternatives, and $a_{\mathrm{cool}}=0.35$. No local monthly demand data were available.

The plant produces at a constant rate equal to the annual mean of the demand it serves, which is the baseline assumption that capacity follows the demand reached. Storage of *s* months of mean production absorbs the surplus of low-demand months, and product that can be neither delivered nor stored is unplaced. The seasonal unplaced share is the spillover a year after two years of warm-up. The total unplaced share combines it with a non-seasonal component $\rho_{\mathrm{other}}$ for reclaimed-network losses and unapproved periods, declared as 0.04: $\rho_{u}=1-\left(1-\rho_{\mathrm{season}}\right)\left(1-\rho_{\mathrm{other}}\right)$. An alternative design follows demand with capacity sized to the peak month, which removes seasonal spill at a capital cost multiplied by the peak-to-mean ratio. In the ensemble variant, $a_{\mathrm{irr}}∼U\left(0.30,0.60\right)$, $a_{\mathrm{cool}}∼U\left(0.20,0.50\right)$, the pilgrimage position is uniform over the twelve months, s∼U(0,1) month and $\rho_{\mathrm{other}}∼U\left(0.01,0.08\right)$, drawn once per realization and shared by both configurations.

Without storage, the seasonal unplaced share is 10.5% for Configuration A and 11.6% for Configuration B, ranging over the pilgrimage positions from 10.1% to 10.9% and from 11.4% to 11.8%. At amplitudes of 0.30 and 0.60, it is 7.0% and 14.0% for Configuration A and 8.5% and 14.7% for Configuration B (Figure A2). One month of storage reduces it to 2.2% and 3.2%, and two months remove it. The peak-month demand is 1.32 and 1.35 times the annual mean. A plant sized to it has net benefits of 777,000 and 697,000 USD per year at 50% coverage, against 674,000 and 641,000 USD for constant production without storage. In the ensemble variant, the configuration-specific unplaced shares have medians of 10.6% and 11.6%, with 5th to 95th percentiles of 4.6–16.4% and 5.8–17.2%, close to the declared baseline distribution of $\rho_{u}$. Monthly effluent availability is not modeled. Because the plant produces at the mean rate of the demand it serves, effluent would constrain production only if the monthly effluent stream fell below 32% of its annual mean for Configuration A or 69% for Configuration B, at full coverage and nominal recoveries. For a plant sized to the peak month, the thresholds are 42% and 93%, so a seasonal dip in effluent could constrain a peak-sized Configuration B.

## Appendix F. Pilot Power Under Less Favorable Variance Structures

The minimum detectable effect of Section 6.3 assumes the exchangeable correlation of the Hussey and Hughes model. To test that assumption, we model the cluster-period mean of normalized log consumption as $y_{it}=\mu_{t}+\delta X_{it}+C_{it}+e_{it}$, with period fixed effects $\mu_{t}$ and treatment indicator $X_{it}$. The cluster-period effects $C_{it}$ have variance $\tau^{2}$ and correlation $r^{\left|t-s\right|}$ between periods *t* and *s*, the exponential decay of Kasza et al. [55], and r=1 recovers the exchangeable model. The residuals $e_{it}$ have variance $\sigma^{2}/m_{i}+\sigma_{cp}^{2}$, where $m_{i}$ is the relative size of cluster *i* and $\sigma_{cp}$ an additional cluster-period standard deviation representing uneven visitor loads. The variance of the generalized least-squares estimator of δ is the last diagonal element of ${\left(\sum_{i}Z_{i}^{⊤}V_{i}^{-1}Z_{i}\right)}^{-1}$, with $Z_{i}=\left[I_{T},\;X_{i}\right]$ and $V_{i}$ the covariance of cluster *i*. Cluster sizes follow gamma quantiles with coefficient of variation (CV) 0, 0.5 or 1, assigned to steps in ten random orders whose variances are averaged. With r=1, equal sizes and $\sigma_{cp}=0$, the computation reproduces Equation (17) to within $10^{-15}$ in relative terms. The grid covers σ=2.5, 5, 10, 15%, τ=5, 10, 20%, r=1, 0.8, 0.5, the three size distributions and $\sigma_{cp}=0$ or 5%, for four-step stepped wedges of 4 to 100 clusters in steps of four.

**Table A6:** Minimum detectable effect (MDE) for eight and twelve clusters and the number of clusters needed to detect the nominal substitution effects of Configurations A (11.5%) and B (20.4%) and an effect of 10%, four-step stepped wedge, two-sided α=0.05, power 0.8. A dash means more than 100 clusters. CV, coefficient of variation of cluster sizes.

Variance Structure	MDE, 8 Clusters	MDE, 12 Clusters	Clusters, B	Clusters, A	Clusters, 10%
Planning values (σ=5%, τ=10%)	8.0%	6.5%	4	4	8
σ=2.5%	4.0%	3.3%	4	4	4
σ=10%	15.3%	12.5%	8	16	20
σ=15%	22.1%	18.0%	12	32	40
τ=20%	8.1%	6.6%	4	4	8
Correlation decay r=0.8	11.2%	9.1%	4	8	12
Correlation decay r=0.5	13.9%	11.4%	4	12	16
Unequal sizes, CV 1	8.5%	6.7%	4	8	8
Visitor-load variance $\sigma_{cp}=5\%$	11.1%	9.1%	4	8	12
σ=10%, τ=20%, r=0.5, CV 1, $\sigma_{cp}=5\%$	31.8%	26.0%	20	64	84
σ=15%, τ=20%, r=0.5, CV 1, $\sigma_{cp}=5\%$	36.7%	29.9%	28	80	–
